# Gapless genome assembly and epigenetic profiles reveal gene regulation of whole-genome triplication in lettuce

**DOI:** 10.1093/gigascience/giae043

**Published:** 2024-07-11

**Authors:** Shuai Cao, Nunchanoke Sawettalake, Lisha Shen

**Affiliations:** Temasek Life Sciences Laboratory, 1 Research Link, National University of Singapore, Singapore 117604, Singapore; Temasek Life Sciences Laboratory, 1 Research Link, National University of Singapore, Singapore 117604, Singapore; Temasek Life Sciences Laboratory, 1 Research Link, National University of Singapore, Singapore 117604, Singapore; Department of Biological Sciences, Faculty of Science, National University of Singapore, Singapore 117543, Singapore

**Keywords:** gapless genome, lettuce, whole-genome triplication, structural variations, DNA methylation, m^6^A, regeneration

## Abstract

**Background:**

Lettuce, an important member of the Asteraceae family, is a globally cultivated cash vegetable crop. With a highly complex genome (∼2.5 Gb; 2n = 18) rich in repeat sequences, current lettuce reference genomes exhibit thousands of gaps, impeding a comprehensive understanding of the lettuce genome.

**Findings:**

Here, we present a near-complete gapless reference genome for cutting lettuce with high transformability, using long-read PacBio HiFi and Nanopore sequencing data. In comparison to stem lettuce genome, we identify 127,681 structural variations (SVs, present in 0.41 Gb of sequence), reflecting the divergence of leafy and stem lettuce. Interestingly, these SVs are related to transposons and DNA methylation states. Furthermore, we identify 4,612 whole-genome triplication genes exhibiting high expression levels associated with low DNA methylation levels and high *N*^6^-methyladenosine RNA modifications. DNA methylation changes are also associated with activation of genes involved in callus formation.

**Conclusions:**

Our gapless lettuce genome assembly, an unprecedented achievement in the Asteraceae family, establishes a solid foundation for functional genomics, epigenomics, and crop breeding and sheds new light on understanding the complexity of gene regulation associated with the dynamics of DNA and RNA epigenetics in genome evolution.

## Background

Lettuce (*Lactuca sativa L*. NCBI:txid4236), an important member of the highly diverse and successful Asteraceae (also known as Compositae) family of flowering plants, is an economically important vegetable crop cultivated worldwide. It ranks among the most cultivated and consumed vegetables and serves as a prominent natural source of phytonutrients for humans [1]. Cultivated lettuce exhibits diverse morphological variations and can be categorized into several horticultural types, including crisp, cutting (also known as looseleaf), butterhead, cos (also known as romaine), latin, stem (also known as stalk), and oilseed lettuce [2]. It is believed that different types of cultivated lettuce originated from a single domestication event involving their wild progenitor, prickly lettuce (*Lactuca serriola*), near the Caucasus in the Middle East of Asia at approximately 4000 BC [3, 4]. Cutting lettuce, one of the major modern cultivated lettuce, exhibits the capacity to quickly and vigorously produce fresh leaves after being harvested at a few inches above the ground—a characteristic often referred to as “cut-and-come-again” [5], highlighting its regrowth capability. However, it remains largely unknown whether cutting lettuce possesses high potential to be transformed due to its strong regenerative capacity.

A complete and accurate reference genome assembly is instrumental for functional genomic research and plant breeding. Lettuce is diploid with 2n = 2x = 18 chromosomes and has a highly complex genome with an estimated size of ∼2.5 Gb and abundant repeat sequences [6–8]. The first version of the crisp lettuce (cultivar “Salinas”) genome was released in 2017, which was assembled using whole-genome shotgun Illumina reads plus *in vitro* proximity ligation data [6]. In addition to this genome of crisp lettuce, a *de novo* chromosome-scale genome assembly of stem lettuce (cultivar “Yanling”) was generated through a combined approach of single-nucleotide real-time sequencing, optical mapping, chromosome conformation capture (Hi-C) sequencing, and Illumina reads [9]. Both lettuce genomes have presented evidence for a whole-genome triplication event basal to the Asteraceae family and facilitated the study of lettuce gene function and regulation [6, 9–11]. Nevertheless, there are still thousands of gaps in these lettuce genomes, hindering the progress of lettuce genomes, functional genomics, and epigenomics research.

Genome evolution could be profoundly influenced by epigenetic modifications that play essential roles in numerous cellular and biological processes and occur in DNA, histones, and RNA [12, 13]. As a conserved and pervasive epigenetic mark in most eukaryotes, DNA methylation at the C-5 position of cytosine underlies gene regulation and modulates diverse biological processes [14]. In plants, DNA methylation occurs in CG, CHG (H = A, T, or C), and CHH contexts and is not only present on repeat sequences to repress transposon activity for genome stability but also related to chromatin states and structural variations (SVs) [14–17]. CG and CHG methylations are relatively stable across different tissues, while CHH methylation exhibits developmental- and tissue-specific as well as stress-responsive variations in plants [18–24]. In addition to DNA methylation, epigenetic modifications also occur in RNAs. RNA methylation at the N-6 position of adenosine found in many eukaryotes, known as *N*^6^-methyladenosine (m^6^A), represents the most prevalent internal modification in messenger RNAs (mRNAs) and has emerged as an indispensable posttranscriptional regulatory mechanism affecting various mRNA metabolism processes, such as splicing, stability, and translation [25–27]. m^6^A modifications exhibit dynamic changes in different tissues and upon stress stimulation in plants, and they modulate multiple aspects of plant development and stress responses [28–30]. It is a reversible modification deposited by a conserved methyltransferase complex (writers) and removed by demethylases (erasers) [31]. Although DNA methylation and m^6^A RNA modification have been extensively profiled in different plant species, their distribution and roles in gene regulation remain largely unexplored in lettuce.

Herein, we present a near-complete telomere-to-telomere (T2T) genome for cutting lettuce (cultivar “Black Seeded Simpson”) with high transformability, generated through *de novo* assembly based on PacBio HiFi long reads, Hi-C data, and ultra-long reads from Oxford Nanopore Technologies (ONT) sequencing. Using the RNA sequencing (RNA-seq)–based transcriptomics data, whole-genome DNA methylation data, and Nanopore long-read direct RNA sequencing data, we construct genome annotations, detect SVs between cutting and stem lettuce, and explore the genomic and epigenetic features of SVs and whole-genome triplication genes after diploidization. Furthermore, we profile alterations of DNA methylation and transcriptome in lettuce callus. Our study provides the first gapless reference genome for lettuce, serving as a cornerstone in functional genomics and breeding, and signifies a major step forward in understanding the complexity of transcriptional and posttranscriptional regulations associated with the dynamics of DNA and RNA epigenetics during genome evolution.

## Results

### Gapless genome assembly for cutting lettuce with high transformability

**Figure 1:**
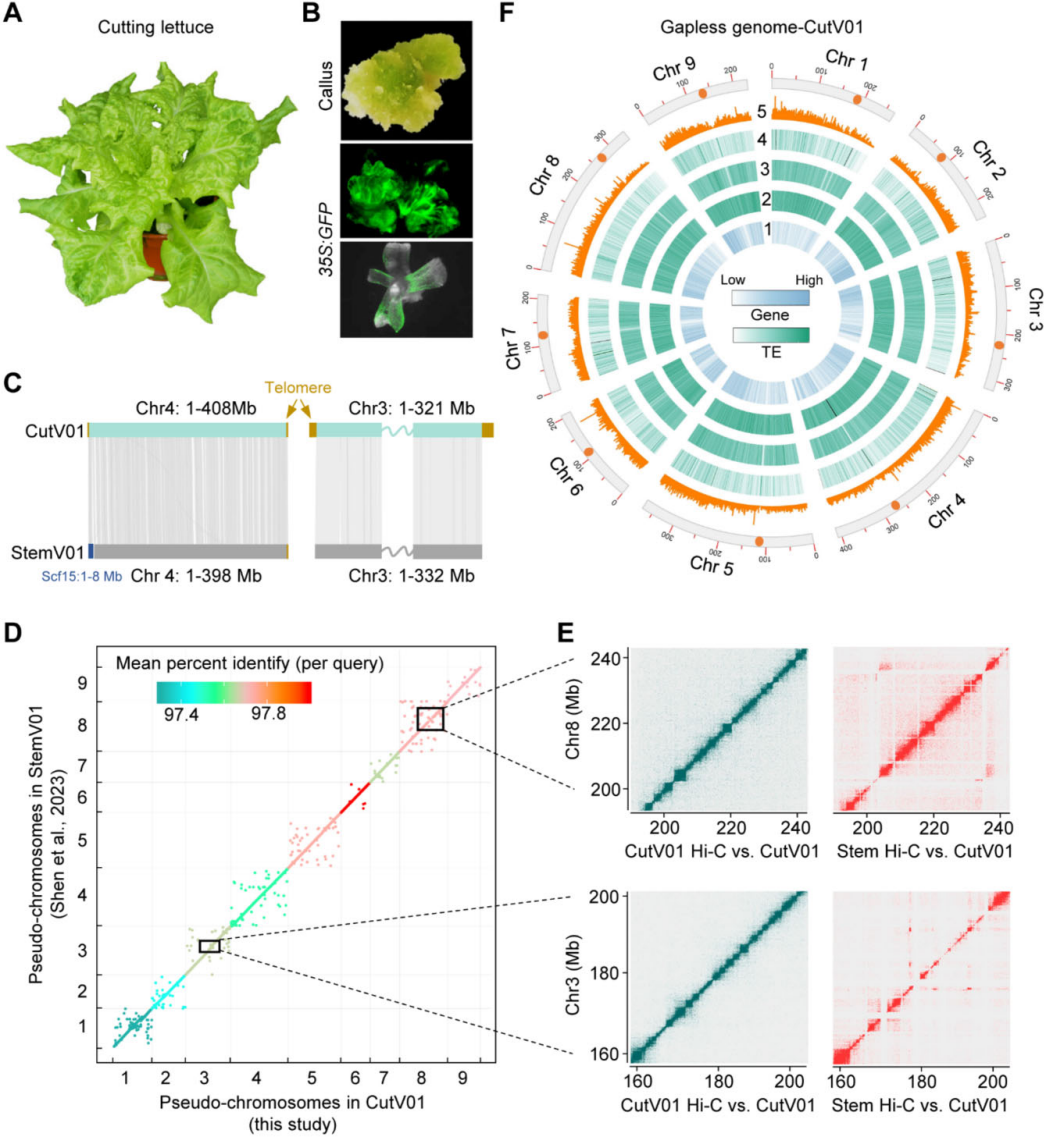
Gapless genome assembly of the cutting lettuce with high transformability. (A) A 6-week-old cutting lettuce cultivar “Black Seeded Simpson.” (B) Regeneration and transformation of the cutting lettuce. Callus (upper panel) was induced from the excised cotyledon, and GFP fluorescence was exhibited by regenerated callus (middle panel) and shoot (low panel) transformed with *35S:GFP*. (C) The collinearity of the longest chromosome, Chr4 (left), and peritelomeric regions of Chr3 (right) between cutting lettuce (CutV01) and stem lettuce (StemV01). Left peritelomeric regions of Chr3: 1–1,024,348 of CutV01 and Chr3: 1–1,027,513 of StemV01 are shown, while right peritelomeric regions of Chr3: 319,140,633–320,995,264 of CutV01 and Chr3: 330,587,218–332,303,623 of StemV01 are illustrated. (D) Dot plots of nucleotide alignment comparing the collinearity and similarity between the genomes of StemV01 and CutV01. Minimum nucleotide alignment length = 1 kb. Boxed regions represent inversions and rearrangements assessed using Hi-C data shown in (E). (E) The chromatin contact Hi-C maps validating the 2 large inversions (20–30 Mb) in Chr8 and Chr3 present between the genomes of CutV01 and StemV01. (F) Circos plot depicting the features of chromosomes in CutV01 assembly: 1, gene density per Mb; 2, Gypsy density per Mb; 3, Copia density per Mb; 4; density of LINEs per Mb; 5, density of DNA Tes. All tracks are intensity-coded, with the color intensity indicating the frequency of each element. Pericentromere, represented by orange color, are depicted on the outmost track of chromosomes, with numbers indicating coordinates in Mb.

Being one of the major modern horticultural types of cultivated lettuce, cutting lettuce exhibits the capacity to quickly and vigorously regrow after being harvested [5], indicating its high regeneration potential. To assess the transformability of cutting lettuce, we first induced callus formation using excised cotyledons from the popular commercial variety “Black Seeded Simpson” (Fig. 1A, B), commonly used in lettuce research [32–34]. We then further optimized an *Agrobacterium tumefaciens*–mediated transformation approach for this cutting lettuce, as demonstrated by the successfully regenerated callus and shoots harboring the *35S:GFP* transgene and exhibiting green fluorescence (Fig. 1B). We achieved a transformation rate (numbers of transgenic shoots versus initial explants) of approximately 30% for this cutting lettuce, indicating that it is easily transformed.

We generated a near-complete T2T genome assembly of this cutting lettuce accession (CutV01), with a total size of 2.58 Gb and contig N50 over 320 Mb, through integrating PacBio HiFi reads, Hi-C reads, and ONT ultra-long reads (Table 1; Supplementary Table S1). *De novo* genome assembly of cutting lettuce was first conducted using PacBio HiFi reads to generate a draft assembly (PacBio V1) of 2.58 Gb. Notably, this initial HiFi assembly PacBio V1 consists of 471 contigs with a contig N50 of 21.54 Mb (Table 1), which is 4- and 12-fold longer than the previously published genomes of the stem lettuce (referred to as StemV01 with a contig N50 of 4.98 Mb) [9] and crisp lettuce (referred to as CrispV08 with a contig N50 of 1.77 Mb) [6]. This HiFi assembly was then scaffolded into pseudo-chromosomes using Hi-C data to yield PacBio V2. The Hi-C data exhibited remarkable consistency across all chromosomes, demonstrating high accuracy of their ordering and orientation (Supplementary Fig. S1A, B). After iteratively polishing using ONT and Hi-C reads, we generated the final near-complete T2T CutV01 genome, encompassing nearly complete telomeres (17 of 18). CutV01 comprises 7 complete T2T pseudo-chromosomes and 2 near-complete chromosomes 4 and 8 with 1 gap in the Chr4 and deficiencies in the long-arm telomere of Chr8 (Supplementary Table S2), representing the highest quality of lettuce genomes reported thus far.

**Table 1:** Assembly statistics of the gapless genome of cutting lettuce CutV01

Assembly metrics	PacBio-V1	PacBio-V2	CutV01
Technology	PacBio	PacBio+Hi-C	PacBio+ONT+Hi-C
Contig number	471	471	10
Contig sequence (bp)	2,597,591,538	2,597,591,538	2,580,598,114
Contig N/L50 (bp)	21,537,486	21,537,486	320,995,264
Contig N/L90 (bp)	8,533,704	8,533,704	230,643,374
Max contig length (bp)	73,015,125	73,015,125	408,427,630
Scaffold number (bp)	471	214	9
Scaffold sequence (bp)	2,597,591,538	2,597,720,038	2,580,598,614
Scaffold N/L50 (bp)	21,537,486	320,952,210	320,995,264
Scaffold N/L90 (bp)	8,533,704	230,490,360	230,643,374
Max scaffold length (bp)	73,015,125	408,496,717	408,463,181
Complete BUSCOs (C)	2,275	2,275	2,275
Complete and single-copy BUSCOs (S)	2,189	2,189	2,189
Complete and duplicated BUSCOs (D)	86	86	86
Fragmented BUSCOs (F)	13	13	13
Missing BUSCOs (M)	38	38	38

CutV01 assembly of the cutting lettuce genome shows significant improvements in drafting lettuce genome. Notably, we identified both telomeres of Chr3, which are absent in the stem lettuce genome (StemV01), and corrected the incompleteness of the longest chromosome in lettuce, Chr4, in StemV01 by identifying Chr4 with more than 400 Mb (Fig. 1C). The CutV01 genome was aligned colinearly with StemV01 and CrispV08 [6, 9], except for 2 large inversions on chromosomes 3 and 8 that were confirmed through Hi-C contact matrix analyses (Fig. 1D, E; Supplementary Fig. S1C, D). To estimate base accuracy of CutV01, we aligned ∼10 Gb Illumina resequencing reads to CutV01 and achieved a high mapping rate of 99.33%. Using the KAD pipeline [35], we obtained an estimated accuracy of approximately 99.93% for genic sequences and 97.80% of all potential errors on transposons or other repetitive sequences. These analyses suggest a high base accuracy of our CutV01 genome. Furthermore, we evaluated the completeness of our CutV01 assembly using BUSCO [36] and found that it contains 97.81% (2275/2326) completeness using the eudicotyledons_odb10 database (Table 1; Supplementary Fig. S2), similar to 97.72% (2273/2326) in CrispV08 and 97.76% (2274/2326) in StemV01, further supporting the high-quality of the CutV01 assembly.

A total of 88.39% of the CutV01 genome sequence is annotated as repetitive elements [37] (Supplementary Fig. S3A; Supplementary Table S3). The most prevalent repetitive elements are the long terminal repeat (LTR) retrotransposons Gypsy and Copia, comprising 41.62% and 30.26% of repeat sequences, respectively (Fig. 1F; Supplementary Fig. S3A; Supplementary Table S4). A small proportion of repetitive elements was annotated as DNA transposable elements (TEs), including hobo-Activator and Tourist/Harbinger transposable repeats, accounting for approximately 1.72% of the CutV01 genome (Fig. 1F; Supplementary Fig. S3A; Supplementary Table S3). Moreover, gene annotation of CutV01 was performed using a combined approaches of *ab initio* gene prediction, homology-based gene prediction, and RNA-seq–based transcriptomics data. In total, we identified 42,406 high-confidence gene models with 67,123 transcripts in the final annotation of CutV01a01 (Fig. 1F; Table 2), which are present in 97.93% (2,228/2,275) of conserved genes evaluated by BUSCO, suggesting high effectiveness in gene annotation (Supplementary Table S4). In addition, more than 72.33% (30,672/42,406) of these genes were annotated with information from Gene Ontology (GO) and KEGG (Supplementary Table S4).

**Table 2:** Summary of CutV01 assembly and annotation

Chromosome	Length (bp)	No. of protein-coding genes	No. of transcripts	Length of repeats
Chr1	252,054,568	4,176	6,885	236,866,928
Chr2	238,860,586	4,292	7,143	208,225,176
Chr3	320,995,264	4,578	7,165	287,668,740
Chr4	408,463,181	5,937	9,238	363,351,330
Chr5	372,789,172	5,594	9,102	330,269,961
Chr6	206,590,668	3,637	5,852	180,287,259
Chr7	208,637,244	4,033	6,061	182,159,041
Chr8	341,564,557	5,591	8,573	303,186,170
Chr9	230,643,374	4,568	7,104	200,914,520
**Total**	2,580,598,614	42,406	67,123	2,292,929,125

### Distribution of structural variations in lettuce

**Figure 2:**
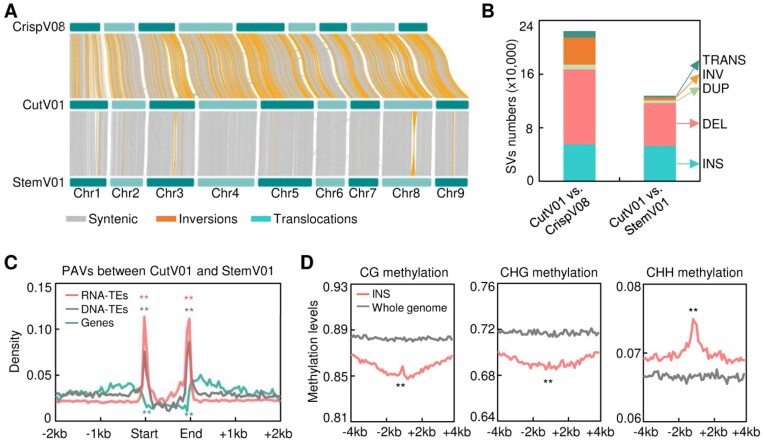
SVs in lettuce genome associated with DNA methylation. (A) Collinearity between the genomes of cutting lettuce (CutV01), crisp lettuce (CrispV08), and stem lettuce (StemV01). The yellow, blue, and gray linking blocks indicate inversions, translocations, and syntenic regions, respectively. (B) SV identification in CutV01 in comparisons to CrispV08 and StemV01. The identified SVs include insertion (INS), deletion (DEL), duplication (DUP), inversion (INV), and translocation (TRANS). (C) Density of PAVs including INS and DEL between CutV01 and StemV01 in the gene regions, retrotransposons (RNA-TEs), and DNA-TEs. Asterisks indicate significance differences (**P < 0.01, Wilcoxon signed-rank test) between the boundaries and flanking regions. (D) Average methylation levels of CG (left), CHG (middle), and CHH (right) around insertions (INS) as compared to the whole genome. Asterisks indicate significance differences (**P < 0.01, Wilcoxon signed-rank test).

Genomic landscapes are shaped by various forms of SVs, including presence/absence variations (PAVs; e.g., insertion and deletions), copy number variations (CNVs), inversions, and translocations [38]. To detect SVs in lettuce, we compared the gapless genome CutV01 of cutting lettuce with the 2 previously reported genomes of crisp lettuce CrispV08 and stem lettuce StemV01 [6, 9]. Surprisingly, genomic collinearity and synteny analysis revealed that CutV01 has 1.74-fold more inversions in comparison to CrispV08 than to StemV01 (Fig. 2A). To further confirm the genomic collinearity, we identified SVs among the 3 genomes. In total, 224,344 SVs were identified in CutV01 compared to CrispV08, containing 55,244 insertions (INS), 111,757 deletions (DEL), 7,161 duplications (DUP), 40,580 inversions (INV), and 9,602 translocations (TRANS) (Fig. 2B), while 127,681 SVs were found in the comparison of CutV01 and StemV01, including 52,741 insertions, 64,147 deletions, 4,164 duplications, 4,302 inversions, and 2,327 translocations (Fig. 2B). Obviously, the number of SVs identified in CutV01 versus CrispV08 was approximately 1.8-fold greater that in CutV01 versus StemV01. Additionally, the total SV length of CutV01 versus CrispV08 (1.43 Gb) exceeded 3 times that of CutV01 versus StemV01 (0.41 Gb), with the length of inversions exhibiting an almost 10-fold difference (Supplementary Fig. S3B) consistent with the genomic collinearity analysis (Fig. 2A). These observations surprisingly contrast with the closer evolutionary relationship between 2 leafy lettuces (cutting and crisp) in comparison to stem lettuce [4], likely due to inevitable errors associated with CrispV08 arisen from the limitations of immature sequencing technology, assembly software, or arithmetic pipelines employed in earlier years. Thus, we focused on the further analysis of SVs identified in CutV01 compared to StemV01.

PAVs, including INS and DEL, accounted for 91.55% of SVs present in CutV01 compared to StemV01 (Fig. 2B). We observed that PAVs were distant from genic regions (Fig. 2C) but enriched in both left and right boundaries of repeat regions, irrespective of TEs of DNA class or RNA class (Fig. 2C). This observation implies a possible association between SV events and TE activity [39].

While the relationship between SVs driven by TEs and DNA methylation has been examined in rice and maize [40, 41], there has been no comprehensive global survey to profile DNA methylation status in relation to nearby SV breakpoints. To explore it, we generated a single-base resolution DNA methylome of cutting lettuce and calculated DNA methylation states across the flanking regions (8 kb) of PAVs identified in the comparison of CutV01 and StemV01. We observed significantly lower DNA methylation levels of CG and CHG across insertion breakpoints in CutV01 compared to the whole genome (Fig. 2D). Although their flanking regions (8 kb) in CutV01 displayed slightly lower CG DNA methylation, deletion breakpoints showed higher CG DNA methylation levels (Supplementary Fig. S3C, D). Remarkably, breakpoints of both insertions and deletions exhibited exceptionally high CHH DNA methylation levels (Fig. 2D; Supplementary Fig. S3E), implying a possible role of CHH methylation in genomic rearrangements during the divergent evolution of leafy and stem lettuce, 2 distinct horticultural types.

### Whole-genome triplication genes retained during lettuce evolution

**Figure 3:**
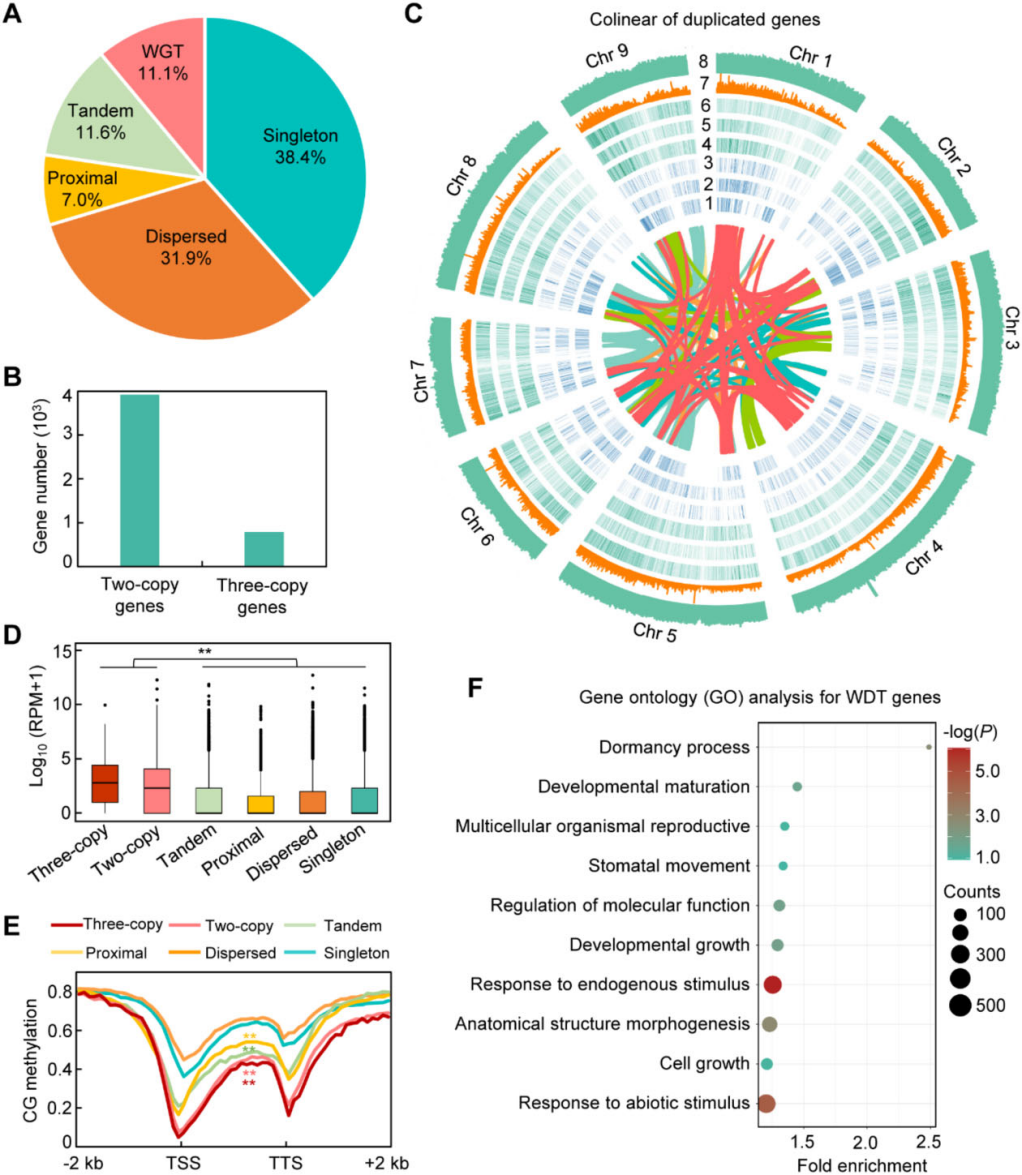
Features of whole-genome duplicated genes. (A) Percentage of WGT, tandem, proximal, dispersed, and single-copy (singleton) genes in annotated genes in CutV01a01. (B) The number of WGT genes retained with 2 copies and 3 copies from whole-genome triplication. (C) Collinearity of duplicated genes cross 9 chromosomes: 1, 3-copy gene density per Mb; 2, 2-copy gene density per Mb; 3, tandem gene density per Mb; 4, proximal genes density per Mb; 5, dispersed genes density per Mb; 6, density of total genes; 7, density of DNA TEs; and 8, density of RNA TEs. (D) Expression levels of 3-copy orthologs, 2-copy orthologs, and tandem, proximal, dispersed, and singleton genes. The asterisk indicates a significant difference (**P < 0.01, Wilcoxon signed-rank test). (E) Average CG methylation levels around 3-copy genes, 2-copy genes, small-scale duplicated (tandem, proximal, and dispersed) genes, and single-copy genes. TSS, transcription start site; TTS, transcription termination site. Asterisks indicate significance differences between DNA methylation of indicated duplicated genes and singleton genes (**P < 0.01, Wilcoxon signed-rank test). (F) GO enrichment of WGT genes. The plot shows the 10 top-scoring biological processes.

While modern lettuce is diploid, recent genome analyses suggest that lettuce underwent whole-genome triplication (WGT) through a paleopolyploidization event proposed and shared by subfamilies near the crown node of the Asteraceae family [6, 9–11, 42]. Unlike the relatively slow process of postpolyploid diploidization in soybean, lettuce genome rapidly lost abundant gene copies of WGT during diploidization, a phenomenon also observed in maize [43]. To examine the features of WGT in lettuce, we performed a comprehensive genomic and epigenomic comparison between duplicated genes in lettuce. Following the previously described classifications of repeat genes in soybean [44], we identified 16,312 single-copy genes, 4,612 WGT genes, and 21,473 small-scale duplicated genes, including 4,921 tandem, 2,966 proximal, and 13,586 dispersed duplicated genes (Fig. 3A). Notably, 17.06% (787/4,612) of these WGT genes retained 3 copies, designated as 3-copy genes [45], while the remaining WGT genes with only 2 copies were termed 2-copy genes (Fig. 3B). Interestingly, analysis of the intragenomic collinearity revealed that most 3-copy genes tended to cluster in chromosomal arms (Fig. 3C), which is consistent with previous observation showing a higher frequency of retained WGT genes in euchromatin regions than pericentromeric regions [43, 46].

To investigate the potential role of epigenetics in gene expression during diploidization, we further analyzed gene expression and local CG DNA methylation patterns. We found that the WGT genes, especially the 3-copy genes, exhibited significantly higher expression levels than small-scale duplicated genes and single-copy genes (Fig. 3D). Interestingly, we observed low CG DNA methylation around genic regions, particularly near transcriptional start sites (TSS), in WGT genes compared to single-copy genes (Fig. 3E), implying that genes with low DNA methylation levels tend to be maintained during the diploidization process in lettuce evolution. Moreover, GO analysis showed that most WGT genes were enriched in response to abiotic stimulus, response to endogenous stimulus, cell growth, anatomical structure morphogenesis, and dormancy process (Fig. 3F), suggesting the possible roles of WGT genes in stress resistance and plant growth during the evolution of cultivated lettuce.

### Shaping the expression of whole-genome duplicated genes by m^6^A modification

It is noteworthy that transcriptional and posttranscriptional regulations orchestrate the balanced expression of genes related to stress resistance and plant growth to ensure the overall health and vitality of organisms [47–49]. To gain deeper insights to the regulation of WGT genes, we deciphered the landscape of m^6^A RNA methylation in lettuce, considering that m^6^A mediates almost all aspects of mRNA metabolism from synthesis to decay and underlies multifaceted developmental processes and stress responses [26, 27]. To this end, we preformed Nanopore long-read direct RNA sequencing to quantitatively locate m^6^A methylation at single-nucleotide resolution in poly(A)-tailed mRNAs. In total, we generated 3.2 million high-quality reads (Q-score > 7) from lettuce seedlings with 3 biological replicates (Supplementary Table S5). Most of these reads displayed high quality with the Q-score of around 11 and had an average read length of 979 to 1,013 nt for each library (Supplementary Table S5) comparable to the typical range of 900 to 1,000 nt observed in *Arabidopsis* mRNA [50, 51]. These observations indicate high integrity of our Nanopore reads that can be used for subsequent analyses.

**Figure 4:**
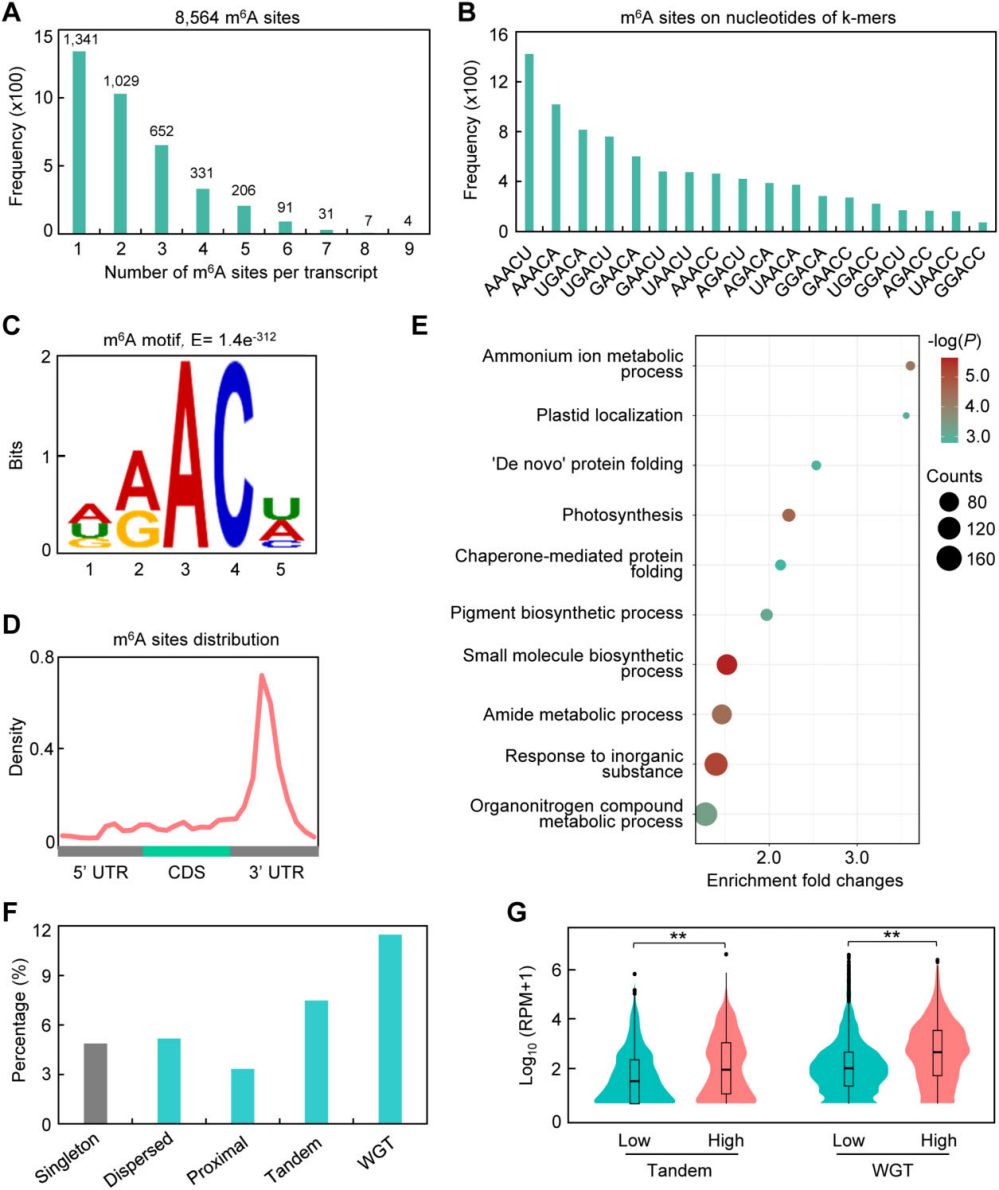
The expression of WGT genes modulated by m^6^A modifications. (A) Frequency of numbers of m^6^A sites per transcript. (B) Frequency of the top 5-bp *k*-mers at the positions with m^6^A sites. (C) Sequence logo representing the consensus motif (DRACH) found in the m^6^A sites. (D) Density of m^6^A sites along the genic region, 5ʹ UTR, and 3ʹ UTR of transcripts. (E) GO enrichment of genes containing m^6^A sites. The plot shows the 10 top-scoring biological processes. (F) Percentage of genes containing m^6^A sites in each repeat type. (G) Expression levels of the tandem and WGT genes with low m^6^A levels (Low) relative to all homoeologous genes with high m^6^A (High). Asterisks indicate significance differences (**P < 0.01, Wilcoxon signed-rank test).

We mapped the Nanopore reads to our annotated transcriptome, CutV01a01, using Minimap2 [52] and observed a mapping rate of >98.73%, supporting a well-annotated CutV01a01 transcriptome (Supplementary Table S5). After calling the signal segmentations using the mapped reads by the Nanopolish software [53], we applied the m6anet algorithm [54] to identify positions of m^6^A modifications for all individual mRNAs. In total, we identified 8,564 high-confidence m^6^A sites that were consistently detected in all 3 biological replicates in 2,505 transcripts in lettuce (Fig. 4A). The top 3 *k*-mers in the positions with m^6^A were AAm^6^ACU, AAm^6^ACA, and UGm^6^ACA, which all contained the sequence of m^6^AC (Fig. 4B). Furthermore, we identified the DRm^6^ACH (D = A/U/G; R = A/G; H = C/A/U) sequence as the most enriched motif among the hypomethylated sites using the HOMER program [55] (Fig. 4C). This motif resembled the conserved RRACH motif enriched in m^6^A sites in various plant species [26]. We further analyzed the distribution of these m^6^A sites along transcripts relative to landmarks in their architecture and found the majority (67.41%) of m^6^A sites were enriched in the 3′ untranslated regions (UTRs) with a clear peak (Fig. 4D), a distribution topology similar to that observed in many eukaryotes [31, 56]. Moreover, the m^6^A-modified transcripts (Supplementary Table S6) were enriched in biological processes such as photosynthesis and a few metabolic processes (Fig. 4E), which could be associated with regulation of growth vigor in lettuce.

To examine whether m^6^A is involved in shaping the expression of WGT genes, we determined m^6^A levels on transcripts of genes categorized into single-copy genes, WGT genes, and small-scale duplicated genes (tandem, proximal, and dispersed duplicated genes) (Fig. 3A). We found that approximately 11.52% of transcripts from WGT genes were modified by m^6^A modifications, ranking highest among different gene types, which was over 2-fold higher than that observed in single-copy genes (Fig. 4F). Moreover, we observed that homoeologous genes with high m^6^A modification levels tended to have higher gene expression compared to those with low m^6^A levels (Fig. 4G). Together, these observations imply a likely role of m^6^A in modulating the expression levels of WGT genes.

### DNA methylation changes during callus induction

**Figure 5:**
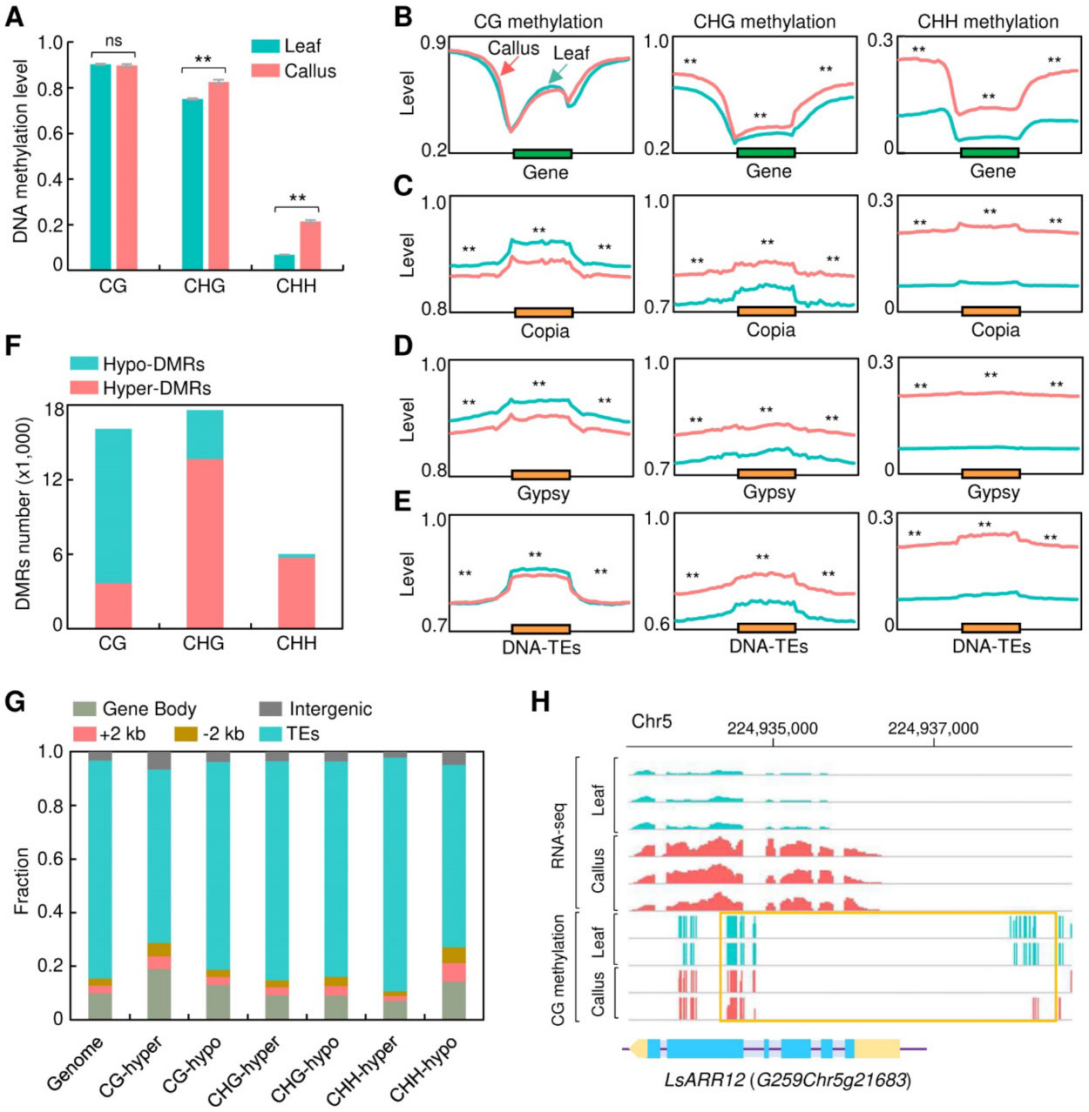
DNA methylation changes during callus formation. (A) Average DNA methylation level of CG, CHG, and CHH in lettuce calli and seedlings. Asterisks and ns indicate significance differences (**P < 0.01, Student’s *t*-test) and no statistical differences (*P* ≥ 0.05, Student’s *t*-test), respectively. (B–E) DNA methylation levels of CG, CHG, and CHH on different genomic features including gene regions (B), Copia (C), Gypsy (D), and DNA-TEs (E). Asterisks indicate significance differences (**P < 0.01, Wilcoxon signed-rank test). (F) Number of hyper- and hypo-DMRs of CG, CHG, and CHH in calli compared to seedlings. (G) Distribution of DMRs in different genomic regions divided into gene body, +2 kb flanking region (2 kb upstream of TSS), −2 kb flanking region (2 kb downstream of TTS), TEs, and intergenic regions excluding TEs. (H) An example showing tissue culture–induced low methylation states of the loci on Chr5: 224,934,400–224,938,500 (yellow box, upper panel) associated with changes in the expression of *LsARR12* (G259Chr5g21683). The gene structure of *LsARR12* is shown below, in which blue and yellow boxes indicate exons and untranslated regions, respectively, and the purple line indicates introns and other genomic regions.

Our results have demonstrated a high-quality gapless genome of the cutting lettuce with high regeneration and transformability, and we thus reasoned that this cutting lettuce could serve as a model system for lettuce functional genomics research and breeding. We then proceeded to examine the epigenetic changes and gene expression in lettuce tissue culture exposed to osmotic pressure and hormone stress during callus induction [57]. We generated a single-base resolution DNA methylome of lettuce calli and observed significantly elevated DNA methylation levels in the CHG and CHH contexts, but not in the CG context, compared to lettuce seedlings (Fig. 5A). Notably, the average methylation level of CHH in calli was approximately 3.5-fold higher than that in seedlings (Fig. 5A). To further understand the distribution of methylation changes in different regions of protein-coding genes and TEs, we calculated the average methylation levels for every 100-bp interval of each gene and TE, encompassing 2-kb upstream and downstream flank regions. Consistently, methylation levels in the CHG and CHH contexts were greatly increased in the 5′ and 3′ regions and gene bodies in calli compared to seedlings (Fig. 5B), while CG methylation levels remained unchanged in all gene regions (Fig. 5B). CHG and CHH methylation levels were much higher across the whole TE regions in calli compared to seedling (Fig. 5C–E). In contrast, TE regions exhibited slightly decreased CG methylation levels in calli, especially in the retrotransposons of Copia and Gypsy (Fig. 5C–E).

To further explore the role of DNA methylation in callus formation, we determined the differentially methylated regions (DMRs) between calli and seedlings. We identified 3,652 hyper- and 12,451 hypo-DMRs of CG methylation, 13,665 hyper- and 3,934 hypo-DMRs of CHG methylation, and 5,731 hyper- and 289 hypo-DMRs of CHH methylation in calli (Fig. 5F). Notably, the number of CG hyper-DMRs was only one-third of that of CG hypo-DMRs, whereas the counts of hyper-DMRs were approximately 3.5-fold and 25-fold higher than those of hypo-DMRs for CHG methylation and CHH methylation, respectively (Fig. 5F). These results were in line with the observed global increases of CHG and CHH methylations in lettuce calli compared to seedlings (Fig. 5A). We then analyzed the distribution of DMRs across genomic features and revealed that CG hyper-DMRs and CHH hypo-DMRs were more prevalent in intergenic and genic regions including 5′ and 3′ flanking regions of coding sequences compared to their average distributions across the whole genome (Fig. 5G). In contrast, most of the CG hypo-DMRs were enriched in gene bodies (Fig. 5G).

To investigate whether DNA methylation changes influence gene expression, we identified 2,496 genes associated with CG DMRs, 1,596 genes associated with CHG DMRs, and 420 genes associated with CHH DMRs, with DMRs located within the 2 kb flanking sequences. Genes associated with these CG DMRs were enriched into biological processes involved in callus formation, such as cell fate specification, specification of axis polarity, and endoderm development (Supplementary Fig. S4A), and notably, these CG DMRs significantly induced expression changes of their associated genes (Supplementary Fig. S4B). In addition to CG DMRs, CHG-DMR–associated genes were overrepresented in the toxin catabolic process, auxin homeostasis, and so on (Supplementary Fig. S5A), while CHH-DMR–associated genes were enriched in the biological processes such as response to toxic substance and cell development (Supplementary Fig. S6A). Hypo-DMRs of CHG and CHH were associated with expression changes of their associated genes (Supplementary Figs. S5B and S6B).

We further identified differently expressed genes in callus compared to seedlings and found significantly increased expression of genes involved in callus formation (Supplementary Fig. S7), such as lettuce homologs of *WUSCHEL* (*WUS*), *ARABIDOPSIS RESPONSE REGULATOR 12* (*ARR12*), *WUSCHEL RELATED HOMEOBOX 13* (*WOX13*), *WRKY23, BABY BOOM* (*BBM*), and *PLETHORA 1* (*PLT1*). Interestingly, we observed decreased CG methylation states in the genic region of *LsARR12* (G259Chr5g21683) associated with its increased expression (Fig. 5H). Notably, ARR12 could directly activate the transcription of *WUS* [58]. Together, these data suggest a transcriptional reprogramming associated with changes in DNA methylation in lettuce callus formation.

## Discussion

With a substantial genome size of approximately 2.6 Gb, the genome of cultivated lettuce, *L. sativa*, is characteristic of many species in the Asteraceae family. In this study, we have generated a gapless genome assembly (CutV01) for cutting lettuce with high transformability. Our near-complete lettuce genome spans a total size of 2.58 Gb and includes 7 T2T and 2 near-complete pseudo-chromosomes, representing an unprecedented high-quality genome in the Asteraceae family.

Using the gapless genome CutV01 and its whole-genome annotations, we have interrogated genomic and epigenomic contributions to SVs, gene duplication, and callus formation in tissue culture. We have identified abundant SVs in the genomes between cutting and stem lettuce [9]. The identified SVs were enriched on the boundaries of repetitive sequences. These boundaries tend to have higher CHH methylation but slightly lower CG and CHG methylations, which could be associated with the activities of transposons [59]. Several studies have identified the widespread presence of SVs, some of which strongly impact the function and expression of genes linked to trait variation [60–62] and environmental stress responses [63, 64]. Due to the strong phenotypic alterations induced by SVs, most SVs may be not maintained during the selective sweep of evolution, especially within genic regions [65, 66]. Consistently, the SVs identified in our study also tend to eschew the gene and its flanking region (Fig. 2C), a pattern reminiscent of T-DNA insertions in rice mutants [15]. It has been suggested that lettuce underwent a WGT event basal to the Asteraceae family [6, 9, 10]. Interestingly, we have identified 4,612 retained WGT genes, which are more than 3,013 genes reported in a previous study based on expressed sequence tag data [11]. These WGT genes also display high expression levels, which is associated with both low DNA methylation levels and high m^6^A RNA modifications.

Our near-complete lettuce genome is assembled using the cutting lettuce cultivar “Black Seeded Simpson” with high regeneration capacity and transformability. Our transformation system for this lettuce requires extensive tissue culture work for callus formation and root regeneration. During plant tissue culture, callus formation involves a process of cell reprogramming of plant somatic cells, which undergo dedifferentiation to somatic embryogenesis [57]. Consistently, transcriptomic analysis in our study reveals that some of genes directly regulating somatic embryogenesis are activated (Supplementary Fig. S7). We have also observed a correlation between DNA methylation and gene activation in callus formation. As an epigenetic mark sensitive to environmental conditions [24], vast DNA methylation changes triggered by tissue culture have also been observed in maize and rice [67, 68]. Interestingly, in lettuce, CG methylation changes influence the expression of genes involved in callus formation, including cell fate specification and specification of axis polarity (Supplementary Fig. S4). In addition, these DNA methylation changes could potentially be transgenerational inheritable [68] and might play a role in priming rapid and strong activation of these genes during callus formation in new tissue culture processes for regenerated lettuce, a phenomenon that remains to be examined.

### Potential implications

Overall, our study reports a near-complete gapless genome of cutting lettuce, containing 7 T2T and 2 near-complete chromosomes, representing the highest completeness and assembly quality for a plant species in the Asteraceae family to date. Comparing with the stem lettuce genome, we identify abundant SVs reflecting the divergence of leafy and stem lettuce. Intriguingly, these SVs are related to transposable elements and DNA methylation states. We further show that retained WGT genes display high expression levels, possibly associated with both low DNA methylation levels and high m^6^A RNA modifications. Moreover, cutting lettuce exhibits high regeneration potential and is easily transformed, and we demonstrate a correlation between DNA methylation and the activation of genes involved in callus formation. Considering the rapidly cycling nature of cutting lettuce, the high-quality reference genome and transformation system for cutting lettuce presented in our study position it as a potential model system for functional genomics research in the Asteraceae family. Taken together, our study provides the first gapless reference genome for lettuce, serving as a cornerstone in functional genomics, epigenomics, and breeding, and signifies a major step forward in understanding the complexity of transcriptional and posttranscriptional regulation associated with the dynamics of DNA and RNA epigenetics during genome evolution.

## Methods

### Plant materials and sampling

Seeds of a commercial cutting lettuce variety “Black Seeded Simpson” were surface sterilized with 10% sodium hypochlorite and grown on soil in a growth chamber with 16 hours of light/8 hours of dark at 24°C (day)/22°C (night). The third pair of leaves were harvested at 30 days after planting (DAP) and immediately frozen in liquid nitrogen for further experiment.

### Genome sequencing by PacBio HiFi and ONT

Frozen leaves were ground into fine powder in liquid nitrogen and transferred to nuclei isolation buffer (40% glycerol, 0.25 M sucrose, 20 mM HEPES, 1 mM MgCl_2_, 5 mM KCl, 0.25% TritonX-100, 0.1 mM PMSF, 1× Protease Inhibitor Cocktail [Roche], and 0.1% 2-mercaptoethanol). After mixing thoroughly, the slurry was kept on ice for 30 minutes and filtered by a 70-μm strainer followed by centrifugation at 3,000 × *g* for 5 minutes. The nuclei pellet was subsequently lysed in 500 μL nuclei lysis buffer (50 mM Tris-HCl, 1% SDS, 10 mM EDTA) supplemented with 10 μg Proteinase K (Roche), from which the genomic DNA was isolated with the DNeasy Plant Mini Kit (Qiagen) following the manufacturer’s protocol. The isolated genomic DNA was used for library constructions for both PacBio HiFi sequencing and ONT ultra-long sequencing. The resulting PacBio and ONT sequencing libraries were run on the PacBio Sequel IIe platform (RRID:SCR_017990) and Nanopore PromethION sequencer (RRID:SCR_017987), respectively, and generated 75 Gb (∼30× genome equivalent) of HiFi reads data and 76 Gb (∼30×) of raw ONT ultra-long-read data.

### Hi-C library construction

Hi-C library was constructed as described previously [44]. Briefly, ∼0.5 g of fresh leaves at 30 DAP was harvested and crosslinked with 1% formaldehyde. The nuclei were extracted using the nuclei isolation buffer as described above. Chromatin in the isolated nuclei was digested by DpnII (NEB), and the digested fragments were filled by biotin-14-dCTP and subsequently proximally ligated with T4 DNA Ligase (NEB). Ligated chromatins were reverse crosslinked and DNA was purified with the QIAquick PCR Purification Kit (Qiagen). Next, the purified DNA was sonicated to produce 300- to 500-bp-long fragments. The sonicated fragments were pulled down by Dynabeads MyOne Streptavidin T1 beads (Invitrogen), end-repaired, and 3′-end adenylated followed by ligation of the adapter (AITbiotech) according to the protocol of NEBNext® Ultra™ II DNA Library Prep Kit for Illumina® (NEB). These adapter-ligated DNA fragments were subsequently amplified by 6-cycle PCR amplification with Q5^®^ HiFi Hot Start DNA Polymerase (NEB). After purification with the VAHTSTM DNA Clean Beads (Vazyme), the Hi-C libraries were sequenced on a NovaSeq platform (Illumina) (RRID:SCR_024569), generating 150-bp paired-end reads.

### Genome assembly

PacBio HiFi reads were used for initial whole-genome assembly by Hifiasm (v0.19.5) (RRID:SCR_021069) [69] with the default parameters. Final contigs of the initial whole-genome assembly were mapped by Hi-C sequencing data consisting of 89 million effective read pairs by Juicer (v.1.6.2) (RRID:SCR_017226) [70] with default parameters and scaffolded to the chromosome-scale assembly by a 3-dimensional *de novo* DNA assembly (3D DNA) pipeline (v.180114) (RRID:SCR_017227) [71] with parameters (-r 3 -m diploid). Finally, we manually modified the assembly error using Juicebox (v.1.8.8) (RRID:SCR_021172) [72] and generated the ultimate scaffolds, of which the largest 9 scaffolds represented 9 chromosomes. The ONT ultra-long-read data were polished by PacBio HiFi data using NextPolish (v1.1.0) (RRID:SCR_025,232) [53] with recommended parameters setting “task = best rewrite = yes rerun = 3” in the parameter config file, and then were used for initial ONT assembly by flye (v2.9.2) (RRID:SCR_017016) [73] with the default parameters. The gaps in the draft scaffold genome-based PacBio HiFi data were filled by polished ultra-long-read data, contigs of the initial ONT assembly by TGS-GapCloser (v1.2.1) (RRID:SCR_017633) [74] and quarTeT (v1.1.4) (RRID:SCR_025252) [75] with default parameters. Pericentromeres were identified by centromics (v0.3) (RRID:SCR_025253) with default parameters.

### RNA-seq library construction and analysis

Total RNA were extracted from various tissues of lettuce, including leaves, roots, stems, and flowers (Supplementary Table S7), using the Trizol reagent (Invitrogen). mRNA was then purified from total RNA using the Dynabeads mRNA purification kit (Invitrogen). Strand-specific mRNA-seq libraries were constructed using the VAHTS Universal V8 RNA-seq library Prep Kit (Vazyme) and sequenced on the NovaSeq platform (Illumina) (RRID:SCR_024569) to generate 150-bp paired-end reads. After filtering the raw reads with fastp (RRID:SCR_016962) [76], clean RNA-seq data were mapped by HISAT2 (v2.1.0) (RRID:SCR_015530) [77] with the parameter (-dta -rnastrandness RF). Next, potential PCR duplicates were removed, and uniquely mapped reads were used to calculate the expression level (FPKM: Reads Per Kilobase of exon model per Million mapped reads) of each gene by StringTie (v.1.3.3b) (RRID:SCR_016323) [78] with parameters (-B -A -rf).

### Analyses of repetitive sequences and TEs

Repeats were *de novo* annotated and classified as a repeat consensus database using RepeatModeler (v2.0.3) (RRID:SCR_015027), and intact LTR retrotransposons were *de novo* annotated using LTR-FINDER (v.1.0.9) (RRID:SCR_015247) [79] and LTR_retriever (v.2.9.5) (RRID:SCR_017623) [80] with default parameters. The final repeat database was used for identifying repeats from the intact LTR masked assembly by RepeatMasker (v. 4.1.2) (RRID:SCR_012954) with parameters (-cutoff 250). We estimated the insertion times of the intact LTR retrotransposons based on nucleotide substitution rate of 7 × 10^−9^ per site per generation (assumed to equal 1 year) by LTR_retriever (v.2.9.5) (RRID:SCR_017623) [80].

### Gene annotation and GO analysis

Gene annotation was conducted by integrating RNA-seq data from multiple tissues, *ab initio* gene prediction, and homology-based gene prediction. Clean RNA-seq reads were mapped onto the gapless genome assembly CutV01 using HISAT2 (v.2.1.0) (RRID:SCR_015530) [77], and transcripts were reconstructed by StringTie (v.1.3.3b) (RRID:SCR_016323) [78]. Simultaneously, Trinity (v.2.1.1) (RRID:SCR_013048) [81] was used to perform genome-guided *de novo* assembly of transcripts with the RNA-seq data. and PASA pipeline (v.2.3.3) (RRID:SCR_014656) [82] was depolyed to predict the gene models with the parameters (–MAX_INTRON_LENGTH 20,000 –transcribed_is_aligned_orient –stringent_alignment_overlap 30.0). Based on the transcript sequences generated by both StringTie and Trinity, candidate coding regions were identified by TransDecoder (v.5.3.0) (RRID:SCR_017647). These gene sets were employed for model training of the *ab initio* gene prediction program AUGUSTUS (v.3.2.2) (RRID:SCR_008417) [83]. AUGUSTUS was then applied for *ab initio* gene prediction based on the repeat-masked genome generated by RepeatMasker. For the homology-based approach, homologous proteins from the *Arabidopsis thaliana, Helianthus annuus, Glycine max, Solanum lycopersicum, Zea mays, Oryza sativa*, and *Setaria italica* genomes were downloaded (Phytozome 13) for the homology-based prediction via Exonerate (v.2.2.0) (RRID:SCR_016088) [84]. Finally, EVidenceModeler (v.1.1.1) (RRID:SCR_014659) [85], with parameters (–segment size 500,000 –overlapSize 10,000), was employed to build a combined gene annotation set (CutV01a01) from these 3 strategies. GO annotations were retrieved by mapping protein sequences to the eggNOG database (RRID:SCR_002456) [86], using DIAMOND (v2.1.5) (RRID:SCR_009457) [87].

### BUSCO assessment

BUSCO (RRID:SCR_015008) was used to assess genome assembly and gene annotation completeness based on the database of eudicotyledons_odb10 [36] with the “genome” and “transcriptome” modes, respectively.

### SV identificaion

To detect PAVs and CNVs, the genomes of CrispV08 and StemV01 were divided into 10-kb windows with 100-bp steps (100× depth of genome) using a previous approach [88] and then mapped onto the gapless genome of CutV01 using minimap2 (v2.18-r1015) (RRID:SCR_018550) with default parameters [52]. Mapped results were sorted by Samtools to call SVs using cuteSV (v1.0.11) (RRID:SCR_025233) with options “-s 10 –r 500 -l 50 -sl 50” [89]. To detect inversion and translocation events, the genomes of CrispV08 and StemV01 were aligned to CutV01 using NUCmer (–c 1000–maxgap=500) (RRID:SCR_018171) [90]. The alignment blocks filtered by the one-to-one alignment mode were then used for the identification of inversion and translocation events by SyRI (v.1.6.3) (RRID:SCR_023008) [91].

### Tissue culture and plant transformation

Seeds of Black Seeded Simpson were sterilized with 70% ethanol for 1 minute followed by 7.5% sodium hypochlorite for 10 minutes. After 3 washes in sterile distilled water, seeds were germinated on half-strength Murashige and Skoog (MS) media at 22°C in a growth chamber. At 5 DAP, cotyledons were excised from seedlings and cut into small sections (1.0–1.5 mm in size) using a sterile blade. These cotyledon sections were cultured on MS medium supplemented with 0.25 mg/L 6-benzylaminopurine (6-BA) and 0.15 mg/L 1-naphthlcetic acid (NAA) under long-day conditions (16 hours of light/8 hours of dark) at 22°C. Subculture was conducted every 14 days. Callus samples at 30 days were harvested and immediately frozen in liquid nitrogen for RNA-seq and MethylC-seq analyses.

To generate *35S:GFP* transgenic lettuce, the entry vector of pENTR-35S-GFP [92] was introduced into the binary vector pHGW to generate pHGW-35S-GFP through the Gateway LR recombination reaction (Invitrogen). For plant transformation, cotyledons excised from seedlings 5 DAP were cut into small sections and immersed in a suspension of the *A. tumefaciens* strain GV3101 carrying the pHGW-35S-GFP vector for 30 minutes, followed by co-cultivation on the MS medium supplemented with 0.25 mg/L 6-BA and 0.15 mg/L NAA in the dark for 2 days. The transformed cotyledons were then transferred to a selection and callus/shoot induction media (MS, 0.25 mg/L 6-BA, 0.15 mg/L NAA, 10 mg/L hygromycin B) and grown under long-day conditions (16 hours of light/8 hours of dark) at 22°C. Subculture was conducted every 14 days. The emerging young shoots were excised for GFP signal observation under a Leica fluorescence stereoscope.

### Identification of m^6^A sites with Nanopore direct RNA sequencing

Nanopore dierct RNA sequencing of lettuce was performed as described previously [93]. Total RNA was extracted from leaves of seedlings at 30 DAP using Trizol reagent (Invitrogen). mRNA was subsequently isolated using the Dynabeads mRNA purification kit (Invitrogen) and assessed by an Agilent Bioanalyzer system. Around 750 ng mRNA was used for library prepartion with the Nanopore direct RNA sequencing kit (SQK-RNA002, ONT). The prepared libraries were loaded onto FLO-MIN106 flow cells and sequenced with the GridION sequencer.

The raw fast5 data were basecalled by Guppy (v4.2.3) (RRID:SCR_022353) with the high-accuracy mode to generate FASTQ files. The FASTQ reads were mapped to the reference transcriptome CutV01a01 of the gapless genome CutV01 using Minimap2 (RRID:SCR_018550) [52]. Alignment was converted to a BAM file by Samtools (RRID:SCR_002105) [94], which was then used for calling signal segmentations by Nanopolish Eventalign (v0.13.2) (RRID:SCR_016157) [95]. The obtained signal segmentations were processed with m6anet (v2.1.0) (RRID:SCR_025234) [54] to detect m^6^A modification sites. The detected m^6^A modification sites were annotated onto the reference annotation dataset CutV01a01 using Perl scripts.

### MethylC-seq library construction and analysis

Genomic DNA was isolated using the cetyltrimethylammonium bromide (CTAB) method [96]. After removing RNA with RNase A (NEB), genomic DNA (around 3 µg) was produced into 300- to 500-bp-long, end-repaired, and 3′-end adenylated fragments followed by ligation of the methylated adapter (AITbiotech) according to the protocol of the NEBNext® Ultra™ II DNA Library Prep Kit for Illumina® (NEB). Subsequently, around 1 μg adapter-ligated DNA fragments was treated with bisulfite using the Zymo EZ DNA Methylation-Gold^™^ kit (Zymo Research), followed by a 10-cycle PCR amplification with Q5U^®^ HiFi Hot Start DNA Polymerase (NEB). After purification with VAHTSTM DNA Clean Beads (Vazyme), the MethylC-seq libraries were sequenced on a NovaSeq platform (Illumina) (RRID:SCR_024569), generating 150-bp paired-end reads.

MethylC-seq reads were subjected to quality control by fastp (RRID:SCR_016962) [76], and the clean reads then were mapped onto the gapless genome CutV01 using Bismark (v0.15.0) (RRID:SCR_005604) with options (-score_min L,0,-0.2 -X 1000) [97]. DMRs were identified using 200-bp sliding windows. The mean methylation level was calculated for each window. Within these candidate regions, DMRs were determined for each comparison by applying cutoff values for average methylation level differences (≥0.5 for CG and CHG and ≥0.1 for CHH) along with a corrected false discovery rate (FDR < 0.05). The FDR was calculated by adjusting *P* values (obtained from analysis of variance tests) of pairwise comparisons using the Benjamini–Hochberg method.

## Supplementary Material

giae043_GIGA-D-24-00037_Original_Submission

giae043_GIGA-D-24-00037_Revision_1

giae043_Response_to_Reviewer_Comments_Original_Submission

giae043_Reviewer_1_Report_Original_Submission Eric Schranz -- 3/31/2024 Reviewed

giae043_Reviewer_2_Report_Original_Submission Yang Dong, Ph.D -- 4/3/2024 Reviewed

giae043_Supplemental_File

## Data Availability

All high-throughput sequencing data of genome, transcriptomes, DNA methylomes and sequence assembly (Accession number: CP145959-CP145967) in this study are available in the Short Read Archive (SRA) under NCBI BioProject accession number PRJNA1077738. These raw data and genome assembly have also been deposited in Genome Sequence Archive (GSA) and Genome Warehouse (GWH) in the BIG Data Center under accession numbers PRJCA021111 and WGS086709. All additional supporting data are available in the *GigaScience* repository, GigaDB [98].

## Additional Files

**Supplementary Fig. S1**. Genome assembly for cutting lettuce. (A, B) Heatmap displaying Hi-C interactions of pseudomolecules of all scaffolds (A) and scaffold 7 (B) in cutting lettuce. (C) Dot plots of nucleotide alignment comparing the collinearity and similarity between the genomes of CrispV08 and CutV01. Minimum nucleotide alignment length = 1 kb. Boxed regions represent inversions and rearrangements assessed using Hi-C data. (D) Chromatin contact Hi-C maps showing 2 large (20–30 Mb) inversions in Chr8 and Chr3 present in genomes of CutV01 compared to CrispV08.

**Supplementary Fig. S2**. BUSCO assessments for different versions of assembled genomes of cutting lettuce. BUSCO assessments reveal the percentages of genes classified into 4 categories: complete and single-copy, complete and duplicated, fragmented, and missing categories.

**Supplementary Fig. S3**. Genomic features of the cutting lettuce genome CutV01. (A) Organization of repetitive sequences in CutV01, inducing RNA-TEs such as LTR retrotransposons (Copia, Gypsy, and others), other RNA TEs, DNA TEs, unclassified TEs (UC TEs), and other repeats. (B) The length of SVs identified in CutV01 in comparison to CrispV08 and StemV01. SVs include insertion (INS), deletion (DEL), duplication (DUP), inversion (INV), and translocation (TRANS). (C–E) Average methylation levels of CG (C), CHG (D), and CHH (E) around deletions (DEL) compared to the whole genome. Asterisks indicate significance differences (***P* < 0.01, Wilcoxon signed-rank test).

**Supplementary Fig. S4**. Characterization of CG DMR-associated genes. (A) GO enrichment of genes associated with CG DMRs. The plot shows the 10 top-scoring biological processes. (B) Increased gene expression changes between calli and seedlings in CG hyper-DMR- and CG hypo-DMR-associated genes compared to all genes. Asterisks indicate significant differences (***P* < 0.01, Wilcoxon signed-rank test).

**Supplementary Fig. S5**. Characterization of CHG DMR-associated genes. (A) GO enrichment of genes associated with CHG DMRs. The plot shows the 10 top-scoring biological processes. (B) Increased gene expression changes between calli and seedlings in CHG hypo-DMR-associated genes compared to all genes. An asterisk indicates a significant difference (***P* < 0.01, Wilcoxon signed-rank test), and ns indicates no significant difference (*P* ≥ 0.05, Wilcoxon signed-rank test).

**Supplementary Fig. S6**. Characterization of CHH DMR-associated genes. (A) GO enrichment of genes associated with CHH DMRs. The plot shows the 10 top-scoring biological processes. (B) Increased gene expression changes between calli and seedlings in CHH hypo-DMR-associated genes compared to all genes. The asterisk indicates a significant difference (***P* < 0.01, Wilcoxon signed-rank test), and ns indicates no significant difference (*P* ≥ 0.05, Wilcoxon signed-rank test).

**Supplementary Fig. S7**. Expression level of genes involved in callus formation. Asterisks indicate significance differences (**P* < 0.05, ***P* < 0.01, 2-tailed paired Student’s *t*-test).

**Supplementary Table S1**. Summary of sequencing data used for genome assembly.

**Supplementary Table S2**. Lettuce genome sequence assembly organized into pseudochromosomes.

**Supplementary Table S3**. Organization of repetitive sequences in the lettuce genome CutV01.

**Supplementary Table S4**. Statistics of protein-coding genes.

**Supplementary Table S5**. Nanopore direct RNA sequencing reads for cutting lettuce.

**Supplementary Table S6**. Gene expression of m^6^A-modified genes.

**Supplementary Table S7**. Transcriptomic data used for genome annotation.

## Abbreviations

6-BA: 6-benzylaminopurine; BUSCO: Benchmarking Universal Single-Copy Orthologs; CNV: copy number variation; CTAB: cetyltrimethylammonium bromide; DAP: days after planting; DEL: deletions; DMR: differentially methylated region; DUP: duplications; FDR: false discovery rate; Gb: gigabases; GO: Gene Ontology; INS: insertions; INV: inversions; KEGG: Kyoto Encyclopedia of Genes and Genomes; LTR: long terminal repeat; m^6^A: N6-methyladenosine; mRNA: messenger RNA; MS: Murashige and Skoog; NAA: 1-naphthlcetic acid; ONT: Oxford Nanopore Technologies; PAV: presence/absence variation; RNA-seq: RNA sequencing; SV: structural variations; T2T: telomere-to-telomere; TE: transposable element; TRANS: translocations; TSS: transcriptional start site; UTR: untranslated region; WGT: whole-genome triplication.
