# Supplementary material for: Gapless genome assembly and epigenetic profiles reveal gene regulation of whole-genome triplication in lettuce
Source: Gigascience. 2024 Jul 11;13:giae043. doi: 10.1093/gigascience/giae043 (PMC11238431; doi:10.1093/gigascience/giae043)
Supplement: giae043_GIGA-D-24-00037_Revision_1 [file giae043_giga-d-24-00037_revision_1.pdf]

GigaScience

Gapless genome assembly and epigenetic profiles reveal gene regulation of whole-genome triplication in lettuce

--Manuscript Draft--

**Manuscript Number:**	GIGA-D-24-00037R1
**Full Title:**	Gapless genome assembly and epigenetic profiles reveal gene regulation of whole-genome triplication in lettuce
**Article Type:**	Research
**Funding Information:**	National Research Foundation Competitive Research Programme (NRF-CRP22-2019-0001) — Dr LISHA SHEN
**Abstract:**	Background: Lettuce, an important member of the Asteraceae family, is a globally cultivated cash vegetable crop. With a highly complex genome (~2.5 Gb; 2n = 18) rich in repeat sequences, current lettuce reference genomes exhibit thousands of gaps, impeding a comprehensive understanding of the lettuce genome.Findings: Here, we present a near-complete gapless reference genome for cutting lettuce with high transformability, using long-read PacBio HiFi and Nanopore sequencing data. In comparison to stem lettuce genome, we identify 127,681 structural variations (SVs, present in 0.41 Gb of sequence), reflecting the divergence of leafy and stem lettuce. Interestingly, these SVs are related to transposons and DNA methylation states. Furthermore, we identify 4,612 whole-genome triplication genes exhibiting high expression levels associated with low DNA methylation levels and high N6-methyladenosine (m6A) RNA modifications. DNA methylation changes are also associated with activation of genes involved in callus formation.Conclusions: Our gapless lettuce genome assembly, an unprecedented achievement in the Asteraceae family, establish a solid foundation for functional genomics, epigenomics, and crop breeding, and shed new light on understanding the complexity of gene regulation associated with the dynamics of DNA and RNA epigenetics in genome evolution.
**Corresponding Author:**	LISHA SHEN<br>Temasek Life Sciences Laboratory Ltd<br>Singapore, SINGAPORE
**Corresponding Author Secondary Information:**	
**Corresponding Author's Institution:**	Temasek Life Sciences Laboratory Ltd
**Corresponding Author's Secondary Institution:**	
**First Author:**	Shuai Cao
**First Author Secondary Information:**	
**Order of Authors:**	Shuai Cao
	Nunchanoke Sawettalake
	LISHA SHEN
**Order of Authors Secondary Information:**	
**Response to Reviewers:**	MS No.: GIGA-D-24-00037<br>MS Title: Gapless genome assembly and epigenetic profiles reveal gene regulation of whole-genome triplication in lettuce<br><br>We would like to thank the Editor and two reviewers for the time committed to reviewing this manuscript, and for the detailed suggestions on improving this manuscript. We have revised the manuscript to fully address Reviewer 1's comments as follows.<br><br>Reviewer #1

Powered by Editorial Manager® and ProduXion Manager® from Aries Systems Corporation

This works provides a high-quality and nearly telomere to telomere assembly of stem lettuce with is highly transformable. This is a valuable resource for the lettuce community. In particular, the analysis of CNVs and methylation (for example of transposons) is very informative.
>Author:
We appreciate the positive comments from the reviewer and suggestions on improving this manuscript.

A few points to consider for revision.

Major suggestion:
>Reviewer:
1. The comparisons to Salinas are all done to V08. However, on Genbank V11 is available. It would be extremely valuable to make some comparisons (such as synteny) to this V11 reference since it is already known that V08 has many errors and thus is no longer seen as the reference for lettuce.
>Author:
We thank the reviewer for this suggestion. We have used CrispV11 for SVs calling. As expected, we observed fewer SVs compared to those identified using CrispV08 in our current manuscript (see Figure R1 in the attached Author Response file). However, given the unpublished status of the paper reporting the CrispV11 genome, we are hesitant to incorporate the CrispV11 results into our manuscript. Nevertheless, to address the reviewer’s comment, we have included the collinearity between the genomes of CrispV11 and CutV01 in the attached Author Response file (Figure R1).

>Reviewer:
2. The telomere analysis is convincing, however less is said about pericentromeres. Potentially considering this and its completeness would be interesting.
>Author:
We thank the reviewer for this suggestion. In the revised manuscript, we have identified the pericentromere regions of Cutv01 (the last two columns of new Supplementary Table S2) and found that these pericentromere regions (orange dots depicted on the outmost track of chromosomes; new Figure 1F) displayed a low density of genes. New Supplementary Table S2 and Figure 1F have been included in the revised manuscript.

>Reviewer:
3. For transposon analysis, consider looking at reports of repeat content and types in recent paper on Lactuca virosa genome.
>Author:
As suggested, we have analyzed transposons based on the repeat content and types as recently reported in the Lactuca virosa genome (Xiong et al., 2023), and we have also included the representative LTR elements of Copia and Gypsy (new Supplementary Table S3). New Supplementary Table S3 has been included in the revised manuscript.

Relevant reference
Xiong W, van Workum DM, Berke L, Bakker LV, Schijlen E, Becker FFM, et al. Genome assembly and analysis of Lactuca virosa: implications for lettuce breeding. G3. 2023;13 11 doi:10.1093/g3journal/jkad204.

>Reviewer:
4. Line 208: Genes are said to be "4,706 whole-genome duplicated (WGD)". However, the genome is triplicated (as said). Thus, use Whole genome triplication (WGT) and break this category down into those in two copies or those in three. Perhaps looking at Brassica diploids (rapa or oleracea) also with WGT would be helpful. You do try to do this, but confusing to call WGD-T...this is not typical.
>Author:
We thank the reviewer for this suggestion. As suggested, in the revised text and new Figures 3 and 4, we have used WGT to replace WGD, and changed WGD-D/WGD-T to two-copy/three-copy genes as reported in the Brassica rapa genome (Wang et al., 2011).

Powered by Editorial Manager® and ProduXion Manager® from Aries Systems Corporation

Relevant reference
Wang X, Wang H, Wang J, Sun R, Wu J, Liu S, et al. The genome of the mesopolyploid crop species Brassica rapa. Nat Genet. 2011;43 10:1035-9.

>Reviewer:
5. For the analysis of WGT and duplicated genes (one copy lost) please also compare to earlier analyses (such as reference 11)
>Author:
As suggested, we have compared our analysis of WGT genes with the previous publication (ref 11: Barker et al., 2008). Barker et al. identified a total of 3,013 duplicated genes probably with two or three copies (namely WGT genes in our study) in 27,907 unigenes of lettuce, while in our study, we have identified 4,612 WGT genes in 42,406 annotated genes in the Cutv01 genome. Both numbers of WGT genes and total annotated genes are more than those reported in Barker et al. This is likely due to different gene annotation approaches. For gene annotation, we merged the annotation results from three approaches (ab initio gene predication, homology-based gene predication, and RNA-seq-based transcriptomics data), but Barker et al., only used expressed sequence tag (EST) data. However, since the names or sequences of WGT genes are not provided in Baker et al., 2008, we are unable to further compare the identities of WGT genes in these two studies. We have added the relevant information in the Discussion part as follows: "Interestingly, we have identified 4,612 retained WGT genes, which are more than 3,013 genes reported in a previous study based on expressed sequence tag data [11].".

Relevant reference
Barker MS, Kane NC, Matvienko M, Kozik A, Michelmore RW, Knapp SJ, et al. Multiple paleopolyploidizations during the evolution of the Compositae reveal parallel patterns of duplicate gene retention after millions of years. Mol Biol Evol 2008;25(11):2445-55.

>Reviewer:
6. Many of the callus related genes also link to apomixis related factors, potentially check if Taraxacum Par gene is also unregulated?
>Author:
We thank the reviewer for this suggestion. PAR is a single-copy gene in lettuce (Ls_8X112340, Lssex) (Underwood et al., 2022). We performed a BLAST analysis using the protein sequence of Lssex in CrispV08 (no Lssex annotated in CrispV11) and identified Lssex encoded by G259Chr8g36267 in Cutv01 with an identical protein sequence (see Figure R2 in the attached Author Response file). However, G259Chr8g36267 is not detected in callus or leaf in our RNA-seq data.

Relevant reference
Underwood CJ, Vijverberg K, Rigola D, Okamoto S, Oplaat C, Camp R, et al. A PARTHENOGENESIS allele from apomictic dandelion can induce egg cell division without fertilization in lettuce. Nat Genet. 2022;54 1:84-93.

Minor comments:
>Reviewer:
7. On lines 163 -164 it is said "Surprisingly, genomic collinearity and synteny analysis revealed that CutV01 has significantly more inversions in comparison to CrispV08 than to StemV0" But no statistical support or underlying numbers for this are given. Can this be improved?
>Author:
We thank the reviewer for this suggestion. To our knowledge, there is no appropriate statistical approach for this test. Therefore, in the revised manuscript, we have changed this sentence to "Surprisingly, genomic collinearity and synteny analysis revealed that CutV01 has 1.74-fold more inversions in comparison to CrispV08 than to StemV01 (Fig. 2A).".

>Reviewer:
8. The number of significant digits (such as for % is sometimes one and sometimes two). Please choose one and be consistent
>Author:
As suggested, we have changed the significant digits of the results to consistently include two numbers after the decimal point in the revised manuscript.

Powered by Editorial Manager® and ProduXion Manager® from Aries Systems Corporation

	Reviewer #2<br>This study aims to generate a new genome assembly for cutting lettuce. A near-complete telomere-to-telomere (T2T) genome assembly was generated for cutting lettuce (cultivar 'Black Seeded Simpson') using PacBio HiFi long reads, Hi-C data, and Oxford Nanopore ultra-long reads. The assembly spans 2.6 Gb and includes 7 complete T2T pseudo-chromosomes and 2 near-complete chromosomes, representing the highest quality lettuce genome assembly to date. This is one of the highest quality studies of complex plant genomes that I know of. The data analysis in this study also demonstrates the advantages of the new version of the genome. Therefore, I believe that in terms of the importance of the species, data accessibility, and data quality, this study deserves sufficient attention.<br>>Author:<br>We appreciate the supportive comments from this reviewer.
**Additional Information:**	
**Question**	**Response**
Are you submitting this manuscript to a special series or article collection?	No
**Experimental design and statistics**<br><br>Full details of the experimental design and statistical methods used should be given in the Methods section, as detailed in our Minimum Standards Reporting Checklist. Information essential to interpreting the data presented should be made available in the figure legends.<br><br>Have you included all the information requested in your manuscript?	Yes
**Resources**<br><br>A description of all resources used, including antibodies, cell lines, animals and software tools, with enough information to allow them to be uniquely identified, should be included in the Methods section. Authors are strongly encouraged to cite Research Resource Identifiers (RRIDs) for antibodies, model organisms and tools, where possible.<br><br>Have you included the information requested as detailed in our Minimum Standards Reporting Checklist?	Yes

**Availability of data and materials**<br><br>All datasets and code on which the conclusions of the paper rely must be either included in your submission or deposited in publicly available repositories (where available and ethically appropriate), referencing such data using a unique identifier in the references and in the "Availability of Data and Materials" section of your manuscript.<br><br>Have you have met the above requirement as detailed in our Minimum Standards Reporting Checklist?	Yes

Powered by Editorial Manager® and ProduXion Manager® from Aries Systems Corporation

Manuscript
Click here to access/download;Manuscript;Lettuce gapless genome-MS-June 20.docx

Gapless genome assembly and epigenetic profiles reveal gene regulation of whole-genome triplication in lettuce

Shuai Cao[1], Nunchanoke Sawettalake[1], and Lisha Shen[1,2*]

[1]Temasek Life Sciences Laboratory, 1 Research Link, National University of Singapore, Singapore, 117604, Singapore.
[2]Department of Biological Sciences, Faculty of Science, National University of Singapore, 117543, Singapore.
[*]Correspondence address. Lisha Shen, Temasek Life Sciences Laboratory, 1 Research Link, National University of Singapore, Singapore.
E-mail: lisha@tll.org.sg
Shuai Cao [0000-0001-5237-3827]; Nunchanoke Sawettalake [0000-0001-6787-6769]; Lisha Shen [0000-0002-1808-6370]

Abstract

Background: Lettuce, an important member of the Asteraceae family, is a globally cultivated cash vegetable crop. With a highly complex genome (~2.5 Gb; 2n = 18) rich in repeat sequences, current lettuce reference genomes exhibit thousands of gaps, impeding a comprehensive understanding of the lettuce genome.

Findings: Here, we present a near-complete gapless reference genome for cutting lettuce with high transformability, using long-read PacBio HiFi and Nanopore sequencing data. In comparison to stem lettuce genome, we identify 127,681 structural variations (SVs, present in 0.41 Gb of sequence), reflecting the divergence of leafy and stem lettuce. Interestingly, these SVs are related to transposons and DNA methylation states. Furthermore, we identify 4,612 whole-genome triplication genes exhibiting high expression levels associated with low DNA methylation levels and high N^6-methyladenosine (m^6A) RNA modifications. DNA methylation changes are also associated with activation of genes involved in callus formation.

Conclusions: Our gapless lettuce genome assembly, an unprecedented achievement in the Asteraceae family, establish a solid foundation for functional genomics, epigenomics, and crop breeding, and shed new light on understanding the complexity of gene regulation associated with the dynamics of DNA and RNA epigenetics in genome evolution.

Key words: gapless genome, lettuce, whole-genome triplication, structural variations, DNA methylation, m^6A, regeneration

Background

Lettuce (*Lactuca sativa L.* NCBI:txid4236), an important member of the highly diverse and successful Asteraceae (also known as Compositae) family of flowering plants, is an economically important vegetable crop cultivated worldwide. It ranks among the most cultivated and consumed vegetables and serves as a prominent natural source of phytonutrients for humans [1]. Cultivated lettuce exhibits diverse morphological variations and can be categorized into several horticultural types, including crisp, cutting (also known as looseleaf), butterhead, cos (also known as romaine), latin, stem (also known as stalk), and oilseed lettuce [2]. It is believed that different types of cultivated lettuce originated from a single domestication event involving their wild progenitor, prickly lettuce (*L. serriola*), near the Caucasus in the Middle East of Asia at approximately 4,000 BC [3, 4]. Cutting lettuce, one of the major modern cultivated lettuce, exhibits the capacity to quickly and vigorously produce fresh leaves after being harvested at a few inches above the ground – a characteristic often referred to as "cut-and-come-again" [5], highlighting its regrowth capability. However, it remains largely unknown whether cutting lettuce possesses high potential to be transformed due to its strong regenerative capacity.

A complete and accurate reference genome assembly is instrumental for functional genomic research and plant breeding. Lettuce is diploid with 2n = 2x = 18 chromosomes and has a highly complex genome with an estimated size of ~2.5 Gb and abundant repeat sequences [6-8]. The first version of the crisp lettuce (cultivar 'Salinas') genome was released in 2017, which was assembled using whole-genome shotgun Illumina reads plus in vitro proximity ligation data [6]. In addition to this genome of crisp lettuce, a de novo chromosome-scale genome assembly of stem lettuce (cultivar 'Yanling') was generated through a combined approaches of single-nucleotide real-time sequencing, optical mapping, chromosome conformation capture (Hi-C) sequencing, and Illumina reads [9]. Both lettuce

genomes have presented evidence for a whole-genome triplication event basal to the Asteraceae family and facilitated the study of lettuce gene function and regulation [6, 9-11]. Nevertheless, there are still thousands of gaps in these lettuce genomes, hindering the progress of lettuce genomes, functional genomics, and epigenomics research.

Genome evolution could be profoundly influenced by epigenetic modifications that play essential roles in numerous cellular and biological processes and occur in DNA, histones, and RNA [12, 13]. As a conserved and pervasive epigenetic mark in most eukaryotes, DNA methylation at the C-5 position of cytosine underlies gene regulation and modulates diverse biological processes [14]. In plants, DNA methylation occurs in CG, CHG (H = A, T, or C), and CHH contexts and is not only present on repeat sequences to repress transposon activity for genome stability but also related to chromatin states and structural variations (SVs) [14-17]. CG and CHG methylations are relatively stable across different tissues, while CHH methylation exhibits developmental- and tissue-specific as well as stress-responsive variations in plants [18-24]. In addition to DNA methylation, epigenetic modifications also occur in RNAs. RNA methylation at the N-6 position of adenosine found in many eukaryotes, known as N^6-methyladenosine (m^6A), represents the most prevalent internal modification in messenger RNAs (mRNAs) and has emerged as an indispensable posttranscriptional regulatory mechanism affecting various mRNA metabolism processes, such as splicing, stability, and translation [25-27]. m^6A modifications exhibit dynamic changes in different tissues and upon stress stimulation in plants, and modulate multiple aspects of plant development and stress responses [28-30]. It is a reversible modification deposited by a conserved methyltransferase complex (writers) and removed by demethylases (erasers) [31]. Although DNA methylation and m^6A RNA modification have been extensively profiled in different plant species, their distribution and roles in gene regulation remain largely unexplored in lettuce.

Herein, we present a near-complete telomere-to-telomere (T2T) genome for cutting lettuce (cultivar ‘Black Seeded Simpson’) with high transformability, generated through de novo assembly based on PacBio HiFi long reads, Hi-C data, and ultralong reads from Oxford Nanopore Technologies (ONT) sequencing. Using the RNA-seq-based transcriptomics data, whole-genome DNA methylation data, and Nanopore long-read direct RNA sequencing data, we construct genome annotations, detect SVs between cutting and stem lettuce, and explore the genomic and epigenetic features of SVs and whole-genome triplication genes after diploidization. Furthermore, we profile alterations of DNA methylation and transcriptome in lettuce callus. Our study provides the first gapless reference genome for lettuce, serving as a cornerstone in functional genomics and breeding, and signifies a major step forward in understanding the complexity of transcriptional and post-transcriptional regulations associated with the dynamics of DNA and RNA epigenetics during genome evolution.

Results

Gapless genome assembly for cutting lettuce with high transformability

Being one of the major modern horticultural types of cultivated lettuce, cutting lettuce exhibits the capacity to quickly and vigorously regrow after being harvested [5], indicating its high regeneration potential. To assess the transformability of cutting lettuce, we first induced callus formation using excised cotyledons from the popular commercial variety ‘Black Seeded Simpson’ (Fig. 1A, B), commonly used in lettuce research [32-34]. We then further optimized an *Agrobacterium tumefaciens*-mediated transformation approach for this cutting lettuce, as demonstrated by the successfully regenerated callus and shoots harboring the *35S:GFP* transgene and exhibiting green fluorescence (Fig. 1B). We achieved a transformation rate (numbers of transgenic shoots versus initial explants) of approximately 30% for this cutting lettuce, indicating that it is easily transformed.

We generated a near-complete telomere-to-telomere (T2T) genome assembly of this cutting lettuce accession (CutV01), with a total size of 2.58 Gb and contig N50 over 320 Mb, through integrating PacBio HiFi reads, Hi-C reads, and Oxford Nanopore Technology (ONT) ultra-long reads (Table 1; Supplementary Table S1). De novo genome assembly of cutting lettuce was first conducted using PacBio HiFi reads to generate a draft assembly (PacBio V1) of 2.58 Gb. Notably, this initial HiFi assembly PacBio V1 consists of 471 contigs with a contig N50 of 21.54 Mb (Table 1), which is 4- and 12-fold longer than the previously published genomes of the stem lettuce (referred as StemV01 with a contig N50 of 4.98 Mb) [9] and crisp lettuce (referred as CrispV08 with a contig N50 of 1.77 Mb) [6]. This HiFi assembly was then scaffolded into pseudo-chromosomes using Hi-C data to yield PacBio V2. The Hi-C data exhibited remarkable consistency across all chromosomes, demonstrating high accuracy of their ordering and orientation (Supplementary Fig. S1A and S1B). After iteratively polishing using ONT and Hi-C reads, we generated the final near-complete T2T CutV01 genome, encompassing nearly complete telomeres (17 out of 18). CutV01 comprises 7 complete T2T pseudo-chromosomes and 2 near-complete chromosomes 4 and 8 with one gap in the Chr4 and deficiencies in the long-arm telomere of Chr8 (Supplementary Table S2), representing the highest quality of lettuce genomes reported thus far.

CutV01 assembly of the cutting lettuce genome shows significant improvements in drafting lettuce genome. Notably, we identified both telomeres of Chr3, which are absent in the stem lettuce genome (StemV01), and corrected the incompleteness of the longest chromosome in lettuce, Chr4, in StemV01 by identifying Chr4 with more than 400 Mb (Fig. 1C). The CutV01 genome was aligned colinearly with StemV01 and CrispV08 [6, 9], except for two large inversions on chromosomes 3 and 8 that were confirmed through Hi-C contact matrix analyses (Fig. 1D and 1E; Supplementary Fig. S1C and S1D). To estimate base accuracy of CutV01, we aligned ~10 Gb Illumina resequencing reads to CutV01 and achieved

a high mapping rate of 99.33%. Using the KAD pipeline [35], we obtained an estimated accuracy of approximately 99.93% for genic sequences and 97.80% of all potential errors on transposons or other repetitive sequences. These analyses suggest a high base accuracy of our CutV01 genome. Furthermore, we evaluated the completeness of our CutV01 assembly using BUSCO [36] and found that it contains 97.81% (2275/2326) completeness using the eudicotyledons_odb10 database (Table 1; Supplementary Fig. S2), similar to 97.72% (2273/2326) in CrispV08 and 97.76% (2274/2326) in StemV01, further supporting the high-quality of the CutV01 assembly.

A total of 88.39% of the CutV01 genome sequence is annotated as repetitive elements [37] (Supplementary Fig. S3A; Supplementary Table S3). The most prevalent repetitive elements are the long terminal repeat (LTR) retrotransposons Gypsy and Copia, comprising 41.62% and 30.26% of repeat sequences, respectively (Fig. 1F; Supplementary Fig. S3A; Supplementary Table S4). A small proportion of repetitive elements was annotated as DNA transposable elements (TEs), including hobo-Activator and Tourist/Harbinger transposable repeats, accounting for approximately 1.72% of the CutV01 genome (Fig. 1F; Supplementary Fig. S3A; Supplementary Table S3). Moreover, gene annotation of CutV01 was performed using a combined approaches of ab initio gene prediction, homology-based gene prediction, and RNA sequencing (RNA-seq)-based transcriptomics data. In total, we identified 42,406 high-confidence gene models with 67,123 transcripts in the final annotation of CutV01a01 (Fig. 1F; Table 2), which are present in 97.93% (2,228/2,275) of conserved genes evaluated by BUSCO, suggesting high effectiveness in gene annotation (Supplementary Table S4). In addition, more than 72.33% (30,672/42,406) of these genes were annotated with information from Gene Ontology (GO) and Kyoto Encyclopedia of Genes and Genomes (KEGG) (Supplementary Table S4).

Distribution of structural variations in lettuce

Genomic landscapes are shaped by various forms of SVs including presence/absence variations (PAVs; e.g. insertion and deletions), copy number variations (CNVs), inversions, and translocations [38]. To detect SVs in lettuce, we compared the gapless genome CutV01 of cutting lettuce with the two previously reported genomes of crisp lettuce CrispV08 and stem lettuce StemV01 [6, 9]. Surprisingly, genomic collinearity and synteny analysis revealed that CutV01 has 1.74-fold more inversions in comparison to CrispV08 than to StemV01 (Fig. 2A). To further confirm the genomic collinearity, we identified SVs among the three genomes. 224,344 SVs were identified in CutV01 compared to CrispV08, containing 55,244 insertions (INS), 111,757 deletions (DEL), 7,161 duplications (DUP), 40,580 inversions (INV), and 9,602 translocations (TRANS) (Fig. 2B), while 127,681 SVs were found in the comparison of CutV01 and StemV01, including 52,741 insertions, 64,147 deletions, 4,164 duplications, 4,302 inversions, and 2,327 translocations (Fig. 2B). Obviously, the number of SVs identified in CutV01 vs. CrispV08 was approximately 1.8-fold greater that in CutV01 vs. StemV01. Additionally, the total SV length of CutV01 vs. CrispV08 (1.43 Gb) exceeded 3 times that of CutV01 vs. StemV01 (0.41 Gb), with the length of inversions exhibiting an almost 10-fold difference (Supplementary Fig. S3B) consistent with the genomic collinearity analysis (Fig. 2A). These observations surprisingly contrast with the closer evolutionary relationship between two leafy lettuces (cutting and crisp) in comparison to stem lettuce [4], likely due to inevitable errors associated with CrispV08 arisen from the limitations of immature sequencing technology, assembly software, or arithmetic pipelines employed in earlier years. Thus, we focused on the further analysis of SVs identified in CutV01 compared to StemV01.

PAVs, including insertion (INS) and deletion (DEL), accounted for 91.55% of SVs present in CutV01 compared to StemV01 (Fig. 2B). We observed that PAVs were distant from genic regions (Fig. 2C), but enriched in both left and right boundaries of repeat regions,

irrespective of TEs of DNA class or RNA class (Fig. 2C). This observation implies a possible association between SV events and TE activity [39].

While the relationship between SVs driven by TEs and DNA methylation has been examined in rice and maize [40, 41], there has been no comprehensive global survey to profile DNA methylation status in relation to nearby SV breakpoints. To explore it, we generated a single-base resolution DNA methylome of cutting lettuce and calculated DNA methylation states across the flanking regions (8 kb) of PAVs identified in the comparison of CutV01 and StemV01. We observed significantly lower DNA methylation levels of CG and CHG across insertion breakpoints in CutV01 compared to the whole genome (Fig. 2D). Although their flanking regions (8 kb) in CutV01 displayed slightly lower CG DNA methylation, deletion breakpoints showed higher CG DNA methylation levels (Supplementary Fig. S3C and S3D). Remarkably, breakpoints of both insertions and deletions exhibited exceptionally high CHH DNA methylation levels (Fig. 2D; Supplementary Fig. S3E), implying a possible role of CHH methylation in genomic rearrangements during the divergent evolution of leafy and stem lettuce, two distinct horticultural types.

Whole-genome triplication genes retained during lettuce evolution

While modern lettuce is diploid, recent genome analyses suggest that lettuce underwent whole-genome triplication (WGT) through a paleopolyploidization event proposed and shared by subfamilies near the crown node of the Asteraceae family [6, 9-11, 42]. Unlike the relatively slow process of post-polyploid diploidization in soybean, lettuce genome rapidly lost abundant gene copies of WGT during diploidization, a phenomenon also observed in maize [43]. To examine the features of WGT in lettuce, we performed a comprehensive genomic and epigenomic comparison between duplicated genes in lettuce. Following the previously described classifications of repeat genes in soybean [44], we identified 16,312

single-copy genes, 4,612 whole-genome triplicated (WGT) genes, and 21,473 small-scale duplicated genes including 4,921 tandem, 2,966 proximal, and 13,586 dispersed duplicated genes (Fig. 3A). Notably, 17.06% (787/4,612) of these WGT genes retained three copies, designated as three-copy genes [45], while the remaining WGT genes with only two copies were termed two-copy genes (Fig. 3B). Interestingly, analysis of the intragenomic collinearity revealed that most three-copy genes tended to cluster in chromosomal arms (Fig. 3C), which is consistent with previous observation showing a higher frequency of retained WGT genes in euchromatin regions than pericentromeric regions [43, 46].

To investigate the potential role of epigenetics in gene expression during diploidization, we further analyzed genes expression and local CG DNA methylation patterns. We found that the WGT genes, especially the three-copy genes, exhibited significantly higher expression levels than small-scale duplicated genes and single-copy genes (Fig. 3D). Interestingly, we observed low CG DNA methylation around genic regions, particularly near transcriptional start sites (TSS), in WGT genes compared to single-copy genes (Fig. 3E), implying that genes with low DNA methylation levels tend to be maintained during the diploidization process in lettuce evolution. Moreover, gene ontology (GO) analysis showed that most WGT genes were enriched in response to abiotic stimulus, response to endogenous stimulus, cell growth, anatomical structure morphogenesis, and dormancy process (Fig. 3F), suggesting the possible roles of WGT genes in stress resistance and plant growth during the evolution of cultivated lettuce.

Shaping the expression of whole-genome duplicated genes by m^6A modifications

It is noteworthy that transcriptional and post-transcriptional regulations orchestrate the balanced expression of genes related to stress resistance and plant growth to ensure the overall health and vitality of organisms [47-49]. To gain deeper insights to the regulation of WGT

genes, we deciphered the landscape of m^6A RNA methylation in lettuce, considering that m^6A mediates almost all aspects of mRNA metabolism from synthesis to decay and underlies multifaced developmental processes and stress responses [26, 27]. To this end, we preformed Nanopore long-read direct RNA sequencing to quantitatively locate m^6A methylation at single-nucleotide resolution in poly(A)-tailed mRNAs. In total, we generated 3.2 million of high-quality reads (Q-score > 7) from lettuce seedlings with three biological replicates (Supplementary Table S5). Most of these reads displayed high-quality with the Q-score of around 11 and had an average read length of 979-1,013 nt for each library (Supplementary Table S5) comparable to the typical range of 900-1,000 nt observed in *Arabidopsis* mRNA [50, 51]. These observations indicate high integrity of our nanopore reads that can be used for subsequent analyses.

We mapped the Nanopore reads to our annotated transcriptome, CutV01a01, using Minimap2 [52], and observed a mapping rate of > 98.73%, supporting a well-annotated CutV01a01 transcriptome (Supplementary Table S5). After calling the signal segmentations using the mapped reads by the Nanopolish software [53], we applied the m6anet algorithm [54] to identify positions of m^6A modifications for all individual mRNAs. In total, we identified 8,564 high-confidence m^6A sites that were consistently detected in all three biological replicates in 2,505 transcripts in lettuce (Fig. 4A). The top three k-mers in the positions with m^6A were AAm^6ACU, AAm^6ACA, and UGm^6ACA, which all contained the sequence of m^6AC (Fig. 4B). Furthermore, we identified the DRm^6ACH (D = A/U/G; R = A/G; H = C/A/U) sequence as the most enriched motif among the hypomethylated sites using the HOMER program [55] (Fig. 4C). This motif resembled the conserved RRACH motif enriched in m^6A sites in various plant species [26]. We further analyzed the distribution of these m^6A sites along transcripts relative to landmarks in their architecture and found the majority (67.41%) of m^6A sites were enriched in the 3’ untranslated regions (UTRs) with a

clear peak (Fig. 4D), a distribution topology similar to that observed in many eukaryotes [31, 56], Moreover, the m^{6}A-modified transcripts (Supplementary Table S6) were enriched in biological processes such as photosynthesis and a few metabolic processes (Fig. 4E), which could be associated with regulation of growth vigor in lettuce.

To examine whether m^{6}A is involved in shaping the expression of WGT genes, we determined m^{6}A levels on transcripts of genes categorized into single-copy genes, WGT genes, and small-scale duplicated genes (tandem, proximal, and dispersed duplicated genes) (Fig. 3A). We found that approximately 11.52% of transcripts from WGT genes were modified by m^{6}A modifications, ranking highest among different gene types, which was over 2-fold higher than that observed in single-copy genes (Fig. 4F). Moreover, we observed that homoeologous genes with high m^{6}A modification levels tended to have higher gene expression compared to those with low m^{6}A levels (Fig. 4G). Together, these observations imply a likely role of m^{6}A in modulating the expression levels of WGT genes.

DNA methylation changes during callus induction

Our results have demonstrated a high-quality gapless genome of the cutting lettuce with high regeneration and transformability, we thus reasoned that this cutting lettuce could serve as model system for lettuce functional genomics research and breeding. We thus proceeded to examine the epigenetic changes and gene expression in lettuce tissue culture exposed to osmotic pressure and hormone stress during callus induction [57]. We generated a single-base resolution DNA methylome of lettuce calli and observed significantly elevated DNA methylation levels of in the CHG and CHH contexts, but not in the CG context, compared to lettuce seedlings (Fig. 5A). Notably, the average methylation level of CHH in calli was approximately 3.5-folds higher than that in seedlings (Fig. 5A). To further understand the distribution of methylation changes in different regions of protein-coding genes and TEs, we

calculated the average methylation levels for every 100-bp interval of each gene and TE, encompassing 2-kb upstream and downstream flank regions. Consistently, methylation levels in the CHG and CHH contexts were greatly increased in the 5' and 3' regions and gene bodies in calli compared to seedlings (Fig. 5B), while CG methylation levels remained unchanged in all gene regions (Fig. 5B). CHG and CHH methylation levels were much higher across the whole TE regions in calli compared to seedling (Fig. 5C-5E). In contrast, TE regions exhibited slightly decreased CG methylation levels in calli, especially in the retrotransposons of Copia and Gypsy (Fig. 5C-5E).

To further explore the role of DNA methylation in callus formation, we determined the differentially methylated regions (DMRs) between calli and seedlings. We identified 3,652 hyper- and 12,451 hypo-DMRs of CG methylation, 13,665 hyper- and 3,934 hypo-DMRs of CHG methylation, 5,731 hyper- and 289 hypo-DMRs of CHH methylation in calli (Fig. 5F). Notably, the number of CG hyper-DMRs was only one third of that of CG hypo-DMRs, whereas the counts of hyper-DMRs were approximately 3.5-folds and 25-folds higher than those of hypo-DMRs for CHG methylation and CHH methylation, respectively (Fig. 5F). These results were in line with the observed global increases of CHG and CHH methylations in lettuce calli compared to seedlings (Fig. 5A). We then analyzed the distribution of DMRs across genomic features and revealed that CG hyper-DMRs and CHH hypo-DMRs were more prevalent in intergenic regions and genic region including 5' and 3' flanking regions of coding sequences compared to their average distributions across the whole genome (Fig. 5G). In contrast, most of the CG hypo-DMRs were enriched in gene bodies (Fig. 5G).

To investigate whether DNA methylation changes influence gene expression, we identified 2,496 genes associated with CG DMRs, 1,596 genes associated with CHG DMRs, and 420 genes associated with CHH DMRs, with DMRs located within the 2 kb flanking sequences. Genes associated with these CG DMRs were enriched into biological processes

involved in callus formation, such as cell fate specification, specification of axis polarity, and endoderm development (Supplementary Fig. S4A), and notably, these CG DMRs significantly induced expression changes of their associated genes (Supplementary Fig. S4B). In addition to CG DMRs, CHG-DMRs-associated genes were overrepresented in the toxin catabolic process, auxin homeostasis etc. (Supplementary Fig. S5A), while CHH-DMRs-associated genes were enriched in the biological processes such as response to toxic substance and cell development (Supplementary Fig. S6A). Hypo-DMRs of CHG and CHH were associated with expression changes of their associated genes (Supplementary Fig. 5B and 6B).

We further identified differently expressed genes in callus compared to seedlings and found significantly increased expression of genes involved in callus formation (Supplementary Fig. S7), such as lettuce homologs of *WUSCHEL* (*WUS*), *ARABIDOPSIS RESPONSE REGULATOR 12* (*ARR12*), *WUSCHEL RELATED HOMEOBOX 13* (*WOX13*), *WRKY23*, *BABY BOOM* (*BBM*), and *PLETHORA 1* (*PLT1*). Interestingly, we observed decreased CG methylation states in the genic region of *LsARR12* (G259Chr5g21683) associated with its increased expression (Fig. 5H). Notably, ARR12 could directly activate the transcription of *WUS* [58]. Together, these data suggest a transcriptional reprogramming associated with changes in DNA methylation in lettuce callus formation.

Discussion

With a substantial genome size of approximately 2.6 Gb, the genome of cultivated lettuce, *L. sativa*, is characteristic of many species in the Asteraceae family. In this study, we have generated a gapless genome assembly (CutV01) for cutting lettuce with high transformability. Our near-complete lettuce genome spans a total size of 2.58 Gb and includes seven T2T and two near-complete pseudo-chromosomes, representing an unprecedented high-quality genome in the Asteraceae family.

Using the gapless genome CutV01 and its whole genome annotations, we have interrogated genomic and epigenomic contributions to SVs, gene duplication, and callus formation in tissue culture. We have identified abundant SVs in the genomes between cutting and stem lettuce [9]. The identified SVs were enriched on the boundaries of repetitive sequences. These boundaries tend to have higher CHH methylation but slightly lower CG and CHG methylations, which could be associated the activities of transposons [59]. Several studies have identified the widespread presence of SVs, some of which strongly impact the function and expression of genes linked to traits variation [60-62] and environmental stress responses [63, 64]. Due to the strong phenotypic alterations induced by SVs, most SVs may be not maintained during the selective sweep of evolution, especially within genic regions [65, 66]. Consistently, the SVs identified in our study also tend to eschew the gene and its flanking region (Fig. 2C), a pattern reminiscent of T-DNA insertions in rice mutants [15]. It has been suggested that lettuce underwent a WGT event basal to the Asteraceae family [6, 9, 10]. Interestingly, we have identified 4,612 retained WGT genes, which are more than 3,013 genes reported in a previous study based on expressed sequence tag data [11]. These WGT genes also display high expression levels, which is associated with both low DNA methylation levels and high m6A RNA modifications.

Our near-complete lettuce genome is assembled using the cutting lettuce cultivar ‘Black Seeded Simpson’ with high regeneration capacity and transformability. Our transformation system for this lettuce requires extensive tissue culture work for callus formation and root regeneration. During plant tissue culture, callus formation involves a process of cell reprogramming of plant somatic cells, which undergo dedifferentiation to somatic embryogenesis [57]. Consistently, transcriptomic analysis in our study reveals that some of genes directly regulating somatic embryogenesis are activated (Supplementary Fig. S7). We have also observed a correlation between DNA methylation and gene activation in callus

formation. As an epigenetic mark sensitive to environmental conditions [24], vast DNA methylation changes triggered by tissue culture have also been observed in maize and rice [67, 68]. Interestingly, in lettuce, CG methylation changes influence the expression of genes involved in callus formation, including cell fate specification and specification of axis polarity (Supplementary Fig. S4). In addition, these DNA methylation changes could potential be transgenerational inheritable [68] and might play a role in priming rapid and strong activation of these genes during callus formation in new tissue culture processes for regenerated lettuce, a phenomenon that remains to be examined.

Potential implications

Overall, our study reports a near-complete gapless genome of cutting lettuce, containing seven T2T and two near-complete chromosomes, representing the highest completeness and assembly quality for a plant species in the Asteraceae family to date. Comparing with the stem lettuce genome, we identify abundant SVs reflecting the divergence of leafy and stem lettuce. Intriguingly, these SVs are related to transposable elements and DNA methylation states. We further show that retained WGT genes display high expression levels, possibly associated with both low DNA methylation levels and high m6A RNA modifications. Moreover, cutting lettuce exhibits high regeneration potential and is easily transformed, and we demonstrate a correlation between DNA methylation and the activation of genes involved in callus formation. Considering the rapidly cycling nature of cutting lettuce, the high-quality reference genome and transformation system for cutting lettuce presented in our study position it as a potential model system for functional genomics research in the Asteraceae family. Taken together, our study provides the first gapless reference genome for lettuce, serving as a cornerstone in functional genomics, epigenomics, and breeding, and signifies a major step forward in understanding the complexity of transcriptional and post-transcriptional regulation

associated with the dynamics of DNA and RNA epigenetics during genome evolution.

Methods

Plant materials and sampling

Seeds of a commercial cutting lettuce variety 'Black Seeded Simpson' were surface sterilized with 10% sodium hypochlorite and grown on soil in a growth chamber with 16 h light / 8 h dark at 24°C (day) / 22°C (night). The third pair of leaves were harvested at 30 days after planting (30 DAP) and immediately frozen in liquid nitrogen for further experiment.

Genome sequencing by PacBio HiFi and Oxford Nanopore technology

Frozen leaves were ground into fine powder in liquid nitrogen and transferred to nuclei isolation buffer [40% glycerol, 0.25 M sucrose, 20 mM HEPES, 1 mM $MgCl_2$, 5 mM KCl, 0.25% TritonX-100, 0.1 mM PMSF, 1× Protease Inhibitor Cocktail (Roche), and 0.1% 2-mercaptoethanol]. After mixing thoroughly, the slurry was kept on ice for 30 min and filtered by a 70 μm strainer followed by centrifugation at 3,000 g for 5 min. The nuclei pellet was subsequently lysed in 500 μL of nuclei lysis buffer (50 mM Tris-HCl, 1% SDS, 10 mM EDTA) supplemented with 10 μg of Proteinase K (Roche), from which the genomic DNA was isolated with the DNeasy Plant Mini Kit (Qiagen) following the manufacturer's protocol. The isolated genomic DNA was used for library constructions for both PacBio HiFi sequencing and Oxford Nanopore Technology (ONT) ultralong sequencing. The resulting PacBio and ONT sequencing libraries were run on the PacBio Sequel IIe platform (RRID:SCR_017990) and Nanopore PromethION sequencer (RRID:SCR_017987), respectively, and generated 75 gigabases (Gb) (~30× genome equivalent) of HiFi reads data and 76 Gb (~30×) of raw ONT ultralong-reads data.

Hi-C library construction

Hi-C library was constructed as described previously [44]. Briefly, ~ 0.5 g of fresh leaves at 30 DAP were harvested and crosslinked with 1% formaldehyde. The nuclei were extracted using the nuclei isolation buffer as described above. Chromatin in the isolated nuclei was digested by DpnII (NEB), and the digested fragments were filled by biotin-14-dCTP and subsequently proximally ligated with T4 DNA Ligase (NEB). Ligated chromatins were reverse crosslinked and DNA was purified with the QIAquick PCR Purification Kit (Qiagen). Next, the purifid DNA was sonicated to produce 300-500 bp long fragments. The sonicated fragments were pulled down by Dynabeads MyOne Streptavidin T1 beads (Invitrogen), end-repaired, and 3’-end adenylated followed by ligation of the adapter (AITbiotech) according to the protocol of NEBNext® Ultra™ II DNA Library Prep Kit for Illumina® (NEB). These adapter-ligated DNA fragments were subsequently amplified by a 6-cycle of PCR amplification with Q5® HiFi Hot Start DNA Polymerase (NEB). After purification with the VAHTSTM DNA Clean Beads (Vazyme), the Hi-C libraries were sequenced on a NovaSeq platform (Illumina) (RRID:SCR_024569), generating 150 bp paired-end reads.

Genome assembly

PacBio HiFi reads were used for initial whole-genome assembly by Hifiasm (v0.19.5) (RRID:SCR_021069) [69] with the default parameters. Final contigs of the initial whole-genome assembly were mapped by Hi-C sequencing data consisting of 89 million of effective read pairs by Juicer (v.1.6.2) (RRID:SCR_017226) [70] with default parameters and scaffolded to the chromosome-scale assembly by a three-dimensional de novo DNA assembly (3D DNA) pipeline (v.180114) (RRID:SCR_017227) [71] with parameters (-r 3 -m diploid). Finally, we manually modified the assembly error using Juicebox (v.1.8.8) (RRID:SCR_021172) [72] and generated the ultimate scaffolds, of which largest 9 scaffolds

represented 9 chromosomes. The ONT ultralong-reads data were polished by PacBio HiFi data using NextPolish (v1.1.0) (RRID: SCR_025232) [53] with recommended parameters setting 'task = best rewrite = yes rerun = 3' in the parameter config file, and then were used for initial ONT assembly by flye (v2.9.2) (RRID:SCR_017016) [73] with the default parameters. The gaps in the draft scaffold genome based PacBio HiFi data were filled by polished ultralong-reads data, contigs of initial ONT assembly by TGS-GapCloser (v1.2.1) (RRID:SCR_017633) [74] and quarTeT (v1.1.4) (RRID:SCR_025252) [75] with default parameters. Pericentromeres were identified by centromics (v0.3) (RRID:SCR_025253) with default parameters.

RNA-seq library construction and analysis

Total RNA were extracted from various tissues of lettuce, including leaves, roots, stems, and flowers (Supplementary Table S7), using the Trizol reagent (Invitrogen). mRNA was then purfied from total RNA using the Dynabeads mRNA purification kit (Invitrogen). Strand-specific mRNA-seq libraries were constructed using the VAHTS Universal V8 RNA-seq library Prep Kit (Vazyme) and sequenced on the NovaSeq platform (Illumina) (RRID:SCR_024569) to generate 150-bp paired-end reads. After filtering the raw reads with fastp (RRID:SCR_016962) [76], clean RNA-seq data were mapped by HISAT2 (v2.1.0) (RRID:SCR_015530) [77] with the parameter (-dta -rnastrandness RF). Next, potential PCR duplicates were removed and uniquely mapped reads were used to calculate the expression level (FPKM) of each gene by StringTie (v.1.3.3b) (RRID:SCR_016323) [78] with parameters (-B -A -rf).

Analyses of repetitive sequences and TEs

Repeats were de novo annotated and classified as repeat consensus database using

RepeatModeler (v2.0.3) (RRID:SCR_015027) and intact LTR retrotransposons were de novo annotated using LTR-FINDER (v.1.0.9) (RRID:SCR_015247) [79] and LTR_retriever (v.2.9.5) (RRID:SCR_017623) [80] with default parameters. The final repeat database was used for identifying repeats from the intact LTR-maskedassembly by RepeatMasker (v. 4.1.2) (RRID:SCR_012954) with parameters (-cutoff 250). We estimated the insertion times of the intact LTR retrotransposons based on nucleotidesubstitution rate of 7×10^{-9} per site per generation (assumed to equal one year) by LTR_retriever (v.2.9.5) (RRID:SCR_017623) [80].

Gene annotation and GO analysis

Gene annotation was conducted by integrating RNA-seq data from multiple tissues, ab initio gene prediction, and homology-based gene prediction. Clean RNA-seq reads were mapped onto the gapless genome assembly CutV01 using HISAT2 (v.2.1.0) (RRID:SCR_015530) [77] and transcripts were reconstructed by StringTie (v.1.3.3b) (RRID:SCR_016323) [78]. Simultaneously, Trinity (v.2.1.1) (RRID:SCR_013048) [81] was used to perform genome-guided de novo assembly of transcripts with the RNA-seq data and PASA pipeline (v.2.3.3) (RRID:SCR_014656) [82] was depolyed to predict the gene models with the parameters (--MAX_INTRON_LENGTH 20000 --transcribed_is_aligned_orient --stringent_alignment_overlap 30.0). Based on the transcript sequences generated by both StringTie and Trinity, candidate coding regions were identified by TransDecoder (v.5.3.0) (RRID:SCR_017647). These gene sets were employed for model training of the ab initio gene prediction program AUGUSTUS (v.3.2.2) (RRID:SCR_008417) [83]. AUGUSTUS was then applied for ab initio gene prediction based on the repeat-masked genome generated by RepeatMasker. For the homology-based approach, homologous proteins from the *Arabidopsis thaliana*, *Helianthus annuus*, *Glycine max*, *Solanum lycopersicum*, *Zea mays*, *Oryza sativa*, and *Setaria italica* genomes were downloaded (Phytozome 13,) for the homology-based

prediction via Exonerate (v.2.2.0) (RRID:SCR_016088) [84]. Finally, EVidenceModeler (v.1.1.1) (RRID:SCR_014659) [85], with parameters (--segment size 500000 --overlapSize 10000), was employed to build a combined gene annotation set (CutV01a01) from these three strategies. GO annotations were retrieved by mapping protein sequences to the eggNOG database (RRID:SCR_002456) [86], using DIAMOND (v2.1.5) (RRID:SCR_009457) [87].

BUSCO assessment

BUSCO (RRID:SCR_015008) was used to assess of genome assembly and gene annotation completeness based on the database of eudicotyledons_odb10 [36] with the "genome" and "transcriptome" modes, respectively.

SV identificaion

To detect PAVs and CNVs, the genomes of CrispV08 and StemV01 were divided into 10-kb windows with 100-bp steps (100× depth of genome) using previous approach [88], and then mapped onto the gapless genome of CutV01 using minimap2 (v2.18-r1015) (RRID:SCR_018550) with default parameters [52]. Mapped results were sorted by Samtools to call SV using cuteSV (v1.0.11) (RRID:SCR_025233) with options "-s 10 –r 500 -l 50 -sl 50" [89]. To detect inversion and translocation events, the genomes of CrispV08 and StemV01 were aligned to CutV01 using NUCmer (--c 1000--maxgap=500) (RRID:SCR_018171) [90]. The alignment blocks filtered by the one-to-one alignment mode were then used for the identification of inversion and translocation events by SyRI (v.1.6.3) (RRID:SCR_023008) [91].

Tissue culture and plant transformation

Seeds of Black Seeded Simpson were sterilized with 70% ethanol for 1 min followed by 7.5%

sodium hypochlorite for 10 min. After three washes in sterile distilled water, seeds were germinated on half-strength Murashige and Skoog (MS) media at 22°C in a growth chamber. At 5 DAP, cotyledons were excised from seedlings and cut into small sections (1.0 - 1.5 mm in size) using a sterile blade. These cotyledon sections were cultured on MS medium supplemented with 0.25 mg/L 6-benzylaminopurine (6-BA) and 0.15 mg/L 1-naphthlcetic acid (NAA) under long-day conditions (16 h of light / 8 h of dark) at 22°C. Subculture was conducted every 14 days. Callus samples at 30 days were harvested and immediately frozen in liquid nitrogen for RNA-seq and MethylC-seq analyses.

To generate *35S:GFP* transgenic lettuce, the entry vector of pENTR-35S-GFP [92] was introduce into the binary vector pHGW to generate pHGW-35S-GFP through the Gateway LR recombination reaction (Invitrogen). For plant transformation, cotyledons excised from seedlings 5 DAP were cut into small sections and immersed in a suspension of *Agrobacterium tumefaciens* strain GV3101 carrying the pHGW-35S-GFP vector for 30 min, followed by co-cultivation on the MS medium supplemented with 0.25 mg/L 6-BA and 0.15 mg/L NAA in the dark for 2 days. The transformed cotyledons were then transferred to a selection and callus/shoot induction media (MS, 0.25 mg/L 6-BA, 0.15 mg/L NAA, 10 mg/L Hygromycin B) and grown under long-day conditions (16 h of light / 8 h of dark) at 22°C. Subculture was conducted every 14 days. The emerging young shoots were excised for GFP signal observation under a Leica fluorescence stereoscope.

Identification of m^6A sites with Nanopore direct RNA sequencing

Nanopore dierct RNA sequencing of lettuce was performed as described previously [93]. Total RNA was extracted from leaves of seedling at 30 DAP using Trizol reagent (Invitrogen). mRNA was subsequently isolated using the Dynabeads mRNA purification kit (Invitrogen) and assessed by an Agilent Bioanalyzer system. About 750 ng of mRNA were used for library

prepartion with the Nanopore direct RNA sequencing kit (SQK-RNA002, Oxford Nanopore Technologies). The prepared libraries were loaded onto FLO-MIN106 flow cells and sequenced with the GridION sequencer.

The raw fast5 data were basecalled by Guppy (v4.2.3) (RRID:SCR_022353) with the high accuracy mode to generate FASTQ files. The FASTQ reads were mapped to the reference transcriptome CutV01a01 of the gapless genome CutV01 using Minimap2 (RRID:SCR_018550) [52]. Alignment was converted to BAM file by Samtools (RRID:SCR_002105) [94], which was then used for calling signal segmentations by Nanopolish Eventalign (v0.13.2) (RRID:SCR_016157) [95]. The obtained signal segmentations were processed with m6anet (v2.1.0) (RRID:SCR_025234) [54] to detect m^6A modification sites. The detected m^6A modification sites were annotated onto the reference annotation dataset CutV01a01 using Perl scripts.

MethylC-seq library construction and analysis

Genomic DNA was isolated using the cetyltrimethylammonium bromide (CTAB) method [96]. After removing RNA with RNase A (NEB), genomic DNA (about 3 μg) was fragmented into 300-500 bp long, end-repaired, and 3'-end adenylated followed by ligation of the methylated adapter (AITbiotech) according to the protocol of NEBNext® Ultra™ II DNA Library Prep Kit for Illumina® (NEB). Subsequently, around 1 μg of adapter-ligated DNA fragments was treated with bisulfite using the Zymo EZ DNA Methylation-Gold™ kit (Zymo Research), followed by a 10-cycles PCR amplification with Q5U® HiFi Hot Start DNA Polymerase (NEB). After purification with VAHTSTM DNA Clean Beads (Vazyme), the MethylC-seq libraries were sequenced on a NovaSeq platform (Illumina) (RRID:SCR_024569), generating 150 bp paired-end reads.

MethylC-seq reads were subjected to quality control by fastp (RRID:SCR_016962) [76],

and the clean reads then were mapped onto the gapless genome CutV01 using Bismark (v0.15.0) (RRID:SCR_005604) with options (-score_min L,0,-0.2 -X 1000) [97]. DMRs were identified using 200-bp sliding windows. The mean methylation level was calculated for each window. Within these candidate regions, DMRs were determined for each comparison by applying cut-off values for average methylation level differences (>= 0.5 for CG and CHG, and >= 0.1 for CHH) along with a corrected false discovery rate (FDR < 0.05). The FDR was calculated by adjusting *P*-values (obtained from ANOVA tests) of pairwise comparisons using the Benjamini-Hochberg method.

Additional Files

Supplementary Fig. S1. Genome assembly for cutting lettuce.

Supplementary Fig. S2. BUSCO assessments for different versions of assembled genomes of cutting lettuce.

Supplementary Fig. S3. Genomic features of the cutting lettuce genome CutV01.

Supplementary Fig. S4. Characterization of CG DMR-associated genes.

Supplementary Fig. S5. Characterization of CHG DMR-associated genes.

Supplementary Fig. S6. Characterization of CHH DMR-associated genes.

Supplementary Fig. S7. Expression level of genes involved in callus formation.

Supplementary Table S1. Summary of sequencing data used for genome assembly.

Supplementary Table S2. Lettuce genome sequence assembly organized into pseudochromosomes.

Supplementary Table S3. Organization of repetitive sequences in the lettuce genome CutV01.

Supplementary Table S4. Statistics of protein-coding genes.

Supplementary Table S5. Nanopore direct RNA sequencing reads for cutting lettuce.

Supplementary Table S6. Gene expression of m^6A modified genes.

Supplementary Table S7. Transcriptomic data used for genome annotation.

Authors' Contributions

S.C. and L.S. conceived the research. S.C. and N.S. performed experiments. S.C. analyzed the data. S.C. and L.S. wrote the manuscript. All authors read and approved the paper.

Funding

This work was supported by the National Research Foundation Competitive Research Programme (NRF-CRP22-2019-0001), and the intramural research support from Temasek Life Sciences Laboratory.

Data Availability

All high-throughput sequencing data of genome, transcriptomes, DNA methylomes and sequence assembly (Accession number: CP145959-CP145967) in this study are available in the Short Read Archive (SRA) under NCBI BioProject accession number PRJNA1077738. These raw data and genome assembly have also been deposited in Genome Sequence Archive (GSA) and Genome Warehouse (GWH) in BIG Data Center under the accession numbers: PRJCA021111 and WGS086709. All additional supporting data are available in the *GigaScience* repository, GigaDB [98].

Competing Interests

The authors declare that they have no competing interests.

Acknowledgments

We thank the Centre for Bioimaging Sciences of National University of Singapore for providing the computing facility for data analysis. We thank Genome Institute of Singapore, A*STAR for the Nanopore sequencing services.

References

1. Shatilov MV, Razin AF and Ivanova MI. Analysis of the world lettuce market. IOP Conference Series: Earth and Environmental Science 2019;395(1):012053. https://doi.org/10.1088/1755-1315/395/1/012053.
2. Lebeda A, Ryder EJ, Grube R, Dolezˇalova´ I and Krˇ´ıstkova E. Lettuce (Asteraceae; Lactuca spp.). Genetic Resources, Chromosome Engineering, and Crop Improvement 2006;3:377-472. https://doi.org/10.1201/9781420009569.
3. Wei T, van Treuren R, Liu X, Zhang Z, Chen J, Liu Y, et al. Whole-genome resequencing of 445 Lactuca accessions reveals the domestication history of cultivated lettuce. *Nat Genet*. 2021;53(5):752-60. https://doi.org/10.1038/s41588-021-00831-0.
4. Zhang L, Su W, Tao R, Zhang W, Chen J, Wu P, et al. RNA sequencing provides insights into the evolution of lettuce and the regulation of flavonoid biosynthesis. *Nat Commun*. 2017;8(1):2264. https://doi.org/10.1038/s41467-017-02445-9.
5. Cutler KD. Salad gardens: gourmet greens and beyond. Brooklyn Botanic Garden; 1995.
6. Reyes-Chin-Wo S, Wang Z, Yang X, Kozik A, Arikit S, Song C, et al. Genome assembly with in vitro proximity ligation data and whole-genome triplication in lettuce. *Nat Commun*. 2017;8:14953. https://doi.org/10.1038/ncomms14953.
7. W.J.M. Koopman and Jong JHd. A numerical analysis of karyotypes and DNA amounts in lettuce cultivars and species (Lactuca subsect. Lactuca, Compositae). *Acta Bot Neerl.* 1996;45(2):12. https://doi.org/10.1111/j.1438-8677.1996.tb00510.x.
8. Matoba H, Mizutani T, Nagano K, Hoshi Y and Uchiyama H. Chromosomal study of lettuce and its allied species (Lactuca spp., Asteraceae) by means of karyotype analysis and fluorescence in situ hybridization. *Hereditas.* 2007;144(6):235-43. https://doi.org/10.1111/j.2007.0018-0661.02012x.
9. Shen F, Qin Y, Wang R, Huang X, Wang Y, Gao T, et al. Comparative genomics reveals a unique nitrogen-carbon balance system in Asteraceae. *Nat Commun*. 2023;14(1):4334. https://doi.org/10.1038/s41467-023-40002-9.
10. Badouin H, Gouzy J, Grassa CJ, Murat F, Staton SE, Cottret L, et al. The sunflower genome provides insights into oil metabolism, flowering and Asterid evolution. *Nature.* 2017;546(7656):148-52. https://doi.org/10.1038/nature22380.
11. Barker MS, Kane NC, Matvienko M, Kozik A, Michelmore RW, Knapp SJ, et al. Multiple paleopolyploidizations during the evolution of the Compositae reveal parallel patterns of duplicate gene retention after millions of years. *Mol Biol Evol*. 2008;25(11):2445-55. https://doi.org/10.1093/molbev/msn187.
12. Chen X, Xu H, Shu X and Song CX. Mapping epigenetic modifications by sequencing technologies. *Cell Death Differ*. 2023; https://doi.org/10.1038/s41418-023-01213-1.
13. Yi SV and Goodisman MAD. The impact of epigenetic information on genome evolution. P*hilos Trans R Soc Lond B Biol Sci.* 2021;376(1826):20200114. https://doi.org/10.1098/rstb.2020.0114.
14. Zhang H, Lang Z and Zhu JK. Dynamics and function of DNA methylation in plants. *Nat Rev Mol Cell Biol.* 2018;19(8):489-506. https://doi.org/10.1038/s41580-018-0016-

z.

15. Cao S, Chen K, Lu K, Chen S, Zhang X, Shen C, et al. Asymmetric variation in DNA methylation during domestication and de-domestication of rice. *Plant Cell.* 2023;35(9):3429-43. https://doi.org/10.1093/plcell/koad160.
16. Cao S, Wang L, Han T, Ye W, Liu Y, Sun Y, et al. Small RNAs mediate transgenerational inheritance of genome-wide trans-acting epialleles in maize. *Genome Biol*. 2022;23(1):53. https://doi.org/10.1186/s13059-022-02614-0.
17. Zhang Y, Yang L, Kucherlapati M, Hadjipanayis A, Pantazi A, Bristow CA, et al. Global impact of somatic structural variation on the DNA methylome of human cancers. *Genome Biol.* 2019;20(1):209. https://doi.org/10.1186/s13059-019-1818-9.
18. An YC, Goettel W, Han Q, Bartels A, Liu Z and Xiao W. Dynamic changes of genome-wide DNA methylation during soybean seed development. *Sci Rep*. 2017;7(1):12263. https://doi.org/10.1038/s41598-017-12510-4.
19. Bouyer D, Kramdi A, Kassam M, Heese M, Schnittger A, Roudier F, et al. DNA methylation dynamics during early plant life. *Genome Biol.* 2017;18(1):179. https://doi.org/10.1186/s13059-017-1313-0.
20. Crisp PA, Marand AP, Noshay JM, Zhou P, Lu Z, Schmitz RJ, et al. Stable unmethylated DNA demarcates expressed genes and their cis-regulatory space in plant genomes. *Proc Natl Acad Sci USA*. 2020;117(38):23991-4000. https://doi.org/10.1073/pnas.2010250117.
21. Kawakatsu T, Nery JR, Castanon R and Ecker JR. Dynamic DNA methylation reconfiguration during seed development and germination. *Genome Biol*. 2017;18(1):171. https://doi.org/10.1186/s13059-017-1251-x.
22. Lin JY, Le BH, Chen M, Henry KF, Hur J, Hsieh TF, et al. Similarity between soybean and Arabidopsis seed methylomes and loss of non-CG methylation does not affect seed development. *Proc Natl Acad Sci USA*. 2017;114(45):E9730-E9. https://doi.org/10.1073/pnas.1716758114.
23. Narsai R, Gouil Q, Secco D, Srivastava A, Karpievitch YV, Liew LC, et al. Extensive transcriptomic and epigenomic remodelling occurs during Arabidopsis thaliana germination. *Genome Biol.* 2017;18(1):172. https://doi.org/10.1186/s13059-017-1302-3.
24. Wang L, Cao S, Wang P, Lu K, Song Q, Zhao FJ, et al. DNA hypomethylation in tetraploid rice potentiates stress-responsive gene expression for salt tolerance. *Proc Natl Acad Sci USA*. 2021;118(13) https://doi.org/10.1073/pnas.2023981118.
25. Sharma B, Prall W, Bhatia G and Gregory BD. The diversity and functions of plant RNA modifications: what we know and where we go from here. *Annu Rev Plant Biol*. 2023;74:53-85. https://doi.org/10.1146/annurev-arplant-071122-085813.
26. Shen L, Ma J, Li P, Wu Y and Yu H. Recent advances in the plant epitranscriptome. *Genome Biol*. 2023;24(1):43. https://doi.org/10.1186/s13059-023-02872-6.
27. Yue H, Nie X, Yan Z and Weining S. N6-methyladenosine regulatory machinery in plants: composition, function and evolution. *Plant Biotechnol J*. 2019;17(7):1194-208. https://doi.org/10.1111/pbi.13149.
28. Růžička K, Zhang M, Campilho A, Bodi Z, Kashif M, Saleh M, et al. Identification of factors required for m^6A mRNA methylation in *Arabidopsis* reveals a role for the conserved E3 ubiquitin ligase HAKAI. *New Phytol*. 2017;215(1):157-72. https://doi.org/10.1111/nph.14586.
29. Shao Y, Wong CE, Shen L and Yu H. N6-methyladenosine modification underlies messenger RNA metabolism and plant development. *Curr Opin Plant Biol*. 2021;63:102047. https://doi.org/10.1016/j.pbi.2021.102047.
30. Shen L, Liang Z, Gu X, Chen Y, Teo ZW, Hou X, et al. N6-methyladenosine RNA

modification regulates shoot stem sell fate in Arabidopsis. *Dev Cell*. 2016;38(2):186-200. https://doi.org/10.1016/j.devcel.2016.06.008.

31. Shen L, Liang Z, Wong CE and Yu H. Messenger RNA modifications in plants. *Trends Plant Sci.* 2019;24(4):328-41. https://doi.org/10.1016/j.tplants.2019.01.005.
32. Shuck AL. A Growth-Inhibiting Substance in Lettuce Seeds. *Science*. 1935;81(2096):236. https://doi.org/10.1126/science.81.2096.236.
33. Lazof D and Cheeseman JM. Sodium and potassium compartmentation and transport in the roots of intact lettuce plants. *Plant Physiol*. 1988;88(4):1279-84. https://doi.org/10.1104/pp.88.4.1279.
34. Miller A, Adhikari R and Nemali K. Recycling nutrient solution can reduce growth due to nutrient deficiencies in hydroponic production. *Front Plant Sci*. 2020;11:607643. https://doi.org/10.3389/fpls.2020.607643.
35. He C, Lin G, Wei H, Tang H, White FF, Valent B, et al. Factorial estimating assembly base errors using k-mer abundance difference (KAD) between short reads and genome assembled sequences. *NAR Genom Bioinform*. 2020;2(3):lqaa075. https://doi.org/10.1093/nargab/lqaa075.
36. Simao FA, Waterhouse RM, Ioannidis P, Kriventseva EV and Zdobnov EM. BUSCO: assessing genome assembly and annotation completeness with single-copy orthologs. *Bioinformatics*. 2015;31(19):3210-2. https://doi.org/10.1093/bioinformatics/btv351.
37. Xiong W, van Workum DM, Berke L, Bakker LV, Schijlen E, Becker FFM, et al. Genome assembly and analysis of Lactuca virosa: implications for lettuce breeding. *G3*. 2023;13(11). https://doi.org/10.1093/g3journal/jkad204
38. Shi J, Tian Z, Lai J and Huang X. Plant pan-genomics and its applications. *Mol Plant*. 2023;16(1):168-86. https://doi.org/10.1016/j.molp.2022.12.009.
39. Marroni F, Pinosio S and Morgante M. Structural variation and genome complexity: is dispensable really dispensable? *Curr Opin Plant Biol*. 2014;18:31-6. https://doi.org/10.1016/j.pbi.2014.01.003.
40. Zhang L, Yu H, Ma B, Liu G, Wang J, Wang J, et al. A natural tandem array alleviates epigenetic repression of IPA1 and leads to superior yielding rice. *Nat Commun*. 2017;8:14789. https://doi.org/10.1038/ncomms14789.
41. Xu G, Lyu J, Li Q, Liu H, Wang D, Zhang M, et al. Evolutionary and functional genomics of DNA methylation in maize domestication and improvement. *Nat Commun*. 2020;11(1):5539. https://doi.org/10.1038/s41467-020-19333-4.
42. Huang CH, Zhang C, Liu M, Hu Y, Gao T, Qi J, et al. Multiple polyploidization ievents across asteraceae with two nested events in the early history revealed by nuclear phylogenomics. *Mol Biol Evol*. 2016;33(11):2820-35. https://doi.org/10.1093/molbev/msw157.
43. Zhao M, Zhang B, Lisch D and Ma J. Patterns and Consequences of Subgenome Differentiation Provide Insights into the Nature of Paleopolyploidy in Plants. *Plant Cell*. 2017;29(12):2974-94. https://doi.org/10.1105/tpc.17.00595.
44. Wang L, Jia G, Jiang X, Cao S, Chen ZJ and Song Q. Altered chromatin architecture and gene expression during polyploidization and domestication of soybean. *Plant Cell*. 2021;33(5):1430-46. https://doi.org/10.1093/plcell/koab081.
45. Wang X, Wang H, Wang J, Sun R, Wu J, Liu S, et al. The genome of the mesopolyploid crop species Brassica rapa. *Nat Genet*. 2011;43 10:1035-9. https://doi.org/10.1038/ng.919
46. Du J, Tian Z, Sui Y, Zhao M, Song Q, Cannon SB, et al. Pericentromeric effects shape the patterns of divergence, retention, and expression of duplicated genes in the paleopolyploid soybean. *Plant Cell*. 2012;24(1):21-32. https://doi.org/10.1105/tpc.111.092759.

47. Furlan M, de Pretis S and Pelizzola M. Dynamics of transcriptional and post-transcriptional regulation. *Brief Bioinform*. 2021;22(4) https://doi.org/10.1093/bib/bbaa389.
48. Xu L, Yang L and Huang H. Transcriptional, post-transcriptional and post-translational regulations of gene expression during leaf polarity formation. *Cell Res*. 2007;17(6):512-9. https://doi.org/10.1038/cr.2007.45.
49. Vyse K, Faivre L, Romich M, Pagter M, Schubert D, Hincha DK, et al. Transcriptional and post-transcriptional regulation and transcriptional memory of chromatin regulators in response to low temperature. *Front Plant Sci*. 2020;11:39. https://doi.org/10.3389/fpls.2020.00039.
50. Xu T, Wu X, Wong CE, Fan S, Zhang Y, Zhang S, et al. FIONA1-mediated m6A modification regulates the floral transition in Arabidopsis. *Adv Sci.* 2022;9(6):e2103628. https://doi.org/10.1002/advs.202103628.
51. Zhang S, Li R, Zhang L, Chen S, Xie M, Yang L, et al. New insights into Arabidopsis transcriptome complexity revealed by direct sequencing of native RNAs. *Nucleic Acids Res*. 2020;48(14):7700-11. https://doi.org/10.1093/nar/gkaa588.
52. Li H. Minimap2: pairwise alignment for nucleotide sequences. *Bioinformatics*. 2018;34(18):3094-100. https://doi.org/10.1093/bioinformatics/bty191.
53. Hu J, Fan J, Sun Z and Liu S. NextPolish: a fast and efficient genome polishing tool for long-read assembly. *Bioinformatics*. 2020;36(7):2253-5. https://doi.org/10.1093/bioinformatics/btz891.
54. Hendra C, Pratanwanich PN, Wan YK, Goh WSS, Thiery A and Goke J. Detection of m6A from direct RNA sequencing using a multiple instance learning framework. *Nature Methods*. 2022;19(12):1590-8. https://doi.org/10.1038/s41592-022-01666-1.
55. Heinz S, Benner C, Spann N, Bertolino E, Lin YC, Laslo P, et al. Simple combinations of lineage-determining transcription factors prime cis-regulatory elements required for macrophage and B cell identities. *Mol Cell*. 2010;38(4):576-89. https://doi.org/10.1016/j.molcel.2010.05.004.
56. Zhao BS, Roundtree IA and He C. Post-transcriptional gene regulation by mRNA modifications. *Nat Rev Mol Cell Biol*. 2017;18(1):31-42. https://doi.org/10.1038/nrm.2016.132.
57. Ikeuchi M, Favero DS, Sakamoto Y, Iwase A, Coleman D, Rymen B, et al. Molecular mechanisms of plant regeneration. *Annu Rev Plant Biol*. 2019;70:377-406. https://doi.org/10.1146/annurev-arplant-050718-100434.
58. Dai X, Liu Z, Qiao M, Li J, Li S and Xiang F. ARR12 promotes de novo shoot regeneration in Arabidopsis thaliana via activation of WUSCHEL expression. *J Integr Plant Biol*. 2017;59(10):747-58. https://doi.org/10.1111/jipb.12567.
59. Carvalho CM and Lupski JR. Mechanisms underlying structural variant formation in genomic disorders. *Nat Rev Genet*. 2016;17(4):224-38. https://doi.org/10.1038/nrg.2015.25.
60. Zhang L, Hu J, Han X, Li J, Gao Y, Richards CM, et al. A high-quality apple genome assembly reveals the association of a retrotransposon and red fruit colour. *Nat Commun*. 2019;10(1):1494. https://doi.org/10.1038/s41467-019-09518-x.
61. Li N, He Q, Wang J, Wang B, Zhao J, Huang S, et al. Super-pangenome analyses highlight genomic diversity and structural variation across wild and cultivated tomato species. *Nat Genet*. 2023;55(5):852-60. https://doi.org/10.1038/s41588-023-01340-y.
62. Ruggieri AA, Livraghi L, Lewis JJ, Evans E, Cicconardi F, Hebberecht L, et al. A butterfly pan-genome reveals that a large amount of structural variation underlies the evolution of chromatin accessibility. *Genome Res*. 2022;32(10):1862-75. https://doi.org/10.1101/gr.276839.122.

63. Hubner S, Bercovich N, Todesco M, Mandel JR, Odenheimer J, Ziegler E, et al. Sunflower pan-genome analysis shows that hybridization altered gene content and disease resistance. *Nat Plants*. 2019;5(1):54-62. https://doi.org/10.1038/s41477-018-0329-0.
64. Yan H, Sun M, Zhang Z, Jin Y, Zhang A, Lin C, et al. Pangenomic analysis identifies structural variation associated with heat tolerance in pearl millet. *Nat Genet*. 2023;55(3):507-18. https://doi.org/10.1038/s41588-023-01302-4.
65. Ohta T. Slightly deleterious mutant substitutions in evolution. *Nature*. 1973;246(5428):96-8. https://doi.org/10.1038/246096a0.
66. Kimura M. Evolutionary rate at the molecular level. *Nature*. 1968;217(5129):624-6. https://doi.org/10.1038/217624a0.
67. Lin G, He C, Zheng J, Koo DH, Le H, Zheng H, et al. Chromosome-level genome assembly of a regenerable maize inbred line A188. *Genome Biol*. 2021;22(1):175. https://doi.org/10.1186/s13059-021-02396-x.
68. Stroud H, Ding B, Simon SA, Feng S, Bellizzi M, Pellegrini M, et al. Plants regenerated from tissue culture contain stable epigenome changes in rice. *Elife*. 2013;2:e00354. https://doi.org/10.7554/eLife.00354.
69. Cheng H, Concepcion GT, Feng X, Zhang H and Li H. Haplotype-resolved de novo assembly using phased assembly graphs with hifiasm. *Nat Methods*. 2021;18(2):170-5. https://doi.org/10.1038/s41592-020-01056-5.
70. Durand NC, Shamim MS, Machol I, Rao SS, Huntley MH, Lander ES, et al. Juicer provides a one-click system for analyzing loop-resolution Hi-C experiments. *Cell Syst*. 2016;3(1):95-8. https://doi.org/10.1016/j.cels.2016.07.002.
71. Dudchenko O, Batra SS, Omer AD, Nyquist SK, Hoeger M, Durand NC, et al. De novo assembly of the Aedes aegypti genome using Hi-C yields chromosome-length scaffolds. *Science*. 2017;356(6333):92-5. https://doi.org/10.1126/science.aal3327.
72. Durand NC, Robinson JT, Shamim MS, Machol I, Mesirov JP, Lander ES, et al. Juicebox provides a visualization system for Hi-C contact maps with unlimited zoom. *Cell Syst*. 2016;3(1):99-101. https://doi.org/10.1016/j.cels.2015.07.012.
73. Kolmogorov M, Bickhart DM, Behsaz B, Gurevich A, Rayko M, Shin SB, et al. metaFlye: scalable long-read metagenome assembly using repeat graphs. *Nat Methods*. 2020;17(11):1103-10. https://doi.org/10.1038/s41592-020-00971-x.
74. Xu M, Guo L, Gu S, Wang O, Zhang R, Peters BA, et al. TGS-GapCloser: A fast and accurate gap closer for large genomes with low coverage of error-prone long reads. *Gigascience*. 2020;9(9) https://doi.org/10.1093/gigascience/giaa094.
75. Lin Y, Ye C, Li X, Chen Q, Wu Y, Zhang F, et al. quarTeT: a telomere-to-telomere toolkit for gap-free genome assembly and centromeric repeat identification. *Hortic Res*. 2023;10(8):uhad127. https://doi.org/10.1093/hr/uhad127.
76. Chen S, Zhou Y, Chen Y and Gu J. fastp: an ultra-fast all-in-one FASTQ preprocessor. *Bioinformatics*. 2018;34(17):i884-i90. https://doi.org/10.1093/bioinformatics/bty560.
77. Kim D, Langmead B and Salzberg SL. HISAT: a fast spliced aligner with low memory requirements. *Nat Methods*. 2015;12(4):357-60. https://doi.org/10.1038/nmeth.3317.
78. Pertea M, Pertea GM, Antonescu CM, Chang TC, Mendell JT and Salzberg SL. StringTie enables improved reconstruction of a transcriptome from RNA-seq reads. *Nat Biotechnol*. 2015;33(3):290-5. https://doi.org/10.1038/nbt.3122.
79. Xu Z and Wang H. LTR_FINDER: an efficient tool for the prediction of full-length LTR retrotransposons. *Nucleic Acids Res*. 2007;35(Web Server issue):W265-8. https://doi.org/10.1093/nar/gkm286.
80. Ou S and Jiang N. LTR_retriever: a highly accurate and sensitive program for identification of long terminal repeat retrotransposons. *Plant Physiol*.

2018;176(2):1410-22. https://doi.org/10.1104/pp.17.01310.

81. Grabherr MG, Haas BJ, Yassour M, Levin JZ, Thompson DA, Amit I, et al. Full-length transcriptome assembly from RNA-Seq data without a reference genome. *Nat Biotechnol.* 2011;29(7):644-52. https://doi.org/10.1038/nbt.1883.
82. Haas BJ, Delcher AL, Mount SM, Wortman JR, Smith RK, Jr., Hannick LI, et al. Improving the Arabidopsis genome annotation using maximal transcript alignment assemblies. *Nucleic Acids Res*. 2003;31(19):5654-66. https://doi.org/10.1093/nar/gkg770.
83. Stanke M, Diekhans M, Baertsch R and Haussler D. Using native and syntenically mapped cDNA alignments to improve de novo gene finding. *Bioinformatics*. 2008;24(5):637-44. https://doi.org/10.1093/bioinformatics/btn013.
84. Slater GS and Birney E. Automated generation of heuristics for biological sequence comparison. *BMC Bioinformatics*. 2005;6:31. https://doi.org/10.1186/1471-2105-6-31.
85. Haas BJ, Salzberg SL, Zhu W, Pertea M, Allen JE, Orvis J, et al. Automated eukaryotic gene structure annotation using EVidenceModeler and the Program to Assemble Spliced Alignments. *Genome Biol*. 2008;9(1):R7. https://doi.org/10.1186/gb-2008-9-1-r7.
86. Cantalapiedra CP, Hernandez-Plaza A, Letunic I, Bork P and Huerta-Cepas J. eggNOG-mapper v2: functional annotation, orthology assignments, and domain prediction at the metagenomic scale. *Mol Biol Evol*. 2021;38(12):5825-9. https://doi.org/10.1093/molbev/msab293.
87. Buchfink B, Reuter K and Drost HG. Sensitive protein alignments at tree-of-life scale using DIAMOND. *Nat Methods*. 2021;18(4):366-8. https://doi.org/10.1038/s41592-021-01101-x.
88. Cao S, Zhang H, Liu Y, Sun Y and Chen ZJ. Cytoplasmic genome contributions to domestication and improvement of modern maize. *BMC Biol.* 2024;22(1):64. https://doi.org/10.1186/s12915-024-01859-4.
89. Jiang T, Liu Y, Jiang Y, Li J, Gao Y, Cui Z, et al. Long-read-based human genomic structural variation detection with cuteSV. *Genome Biol*. 2020;21(1):189. https://doi.org/10.1186/s13059-020-02107-y.
90. Marcais G, Delcher AL, Phillippy AM, Coston R, Salzberg SL and Zimin A. MUMmer4: A fast and versatile genome alignment system. *PLoS Comput Biol*. 2018;14(1):e1005944. https://doi.org/10.1371/journal.pcbi.1005944.
91. Goel M, Sun H, Jiao WB and Schneeberger K. SyRI: finding genomic rearrangements and local sequence differences from whole-genome assemblies. *Genome Biol*. 2019;20(1):277. https://doi.org/10.1186/s13059-019-1911-0.
92. Shen L, Zhang Y and Sawettalake N. A Molecular switch for FLOWERING LOCUS C activation determines flowering time in Arabidopsis. *Plant Cell.* 2022;34(2):818-33. https://doi.org/10.1093/plcell/koab286.
93. Wong CE, Zhang S, Xu T, Zhang Y, Teo ZWN, Yan A, et al. Shaping the landscape of N6-methyladenosine RNA methylation in Arabidopsis. *Plant Physiol*. 2023;191(3):2045-63. https://doi.org/10.1093/plphys/kiad010.
94. Danecek P, Bonfield JK, Liddle J, Marshall J, Ohan V, Pollard MO, et al. Twelve years of SAMs and BCFtools. Gigascience. 2021,10(2):giab008. doi: 10.1093/gigascience/giab008
95. Loman NJ, Quick J and Simpson JT. A complete bacterial genome assembled de novo using only nanopore sequencing data. *Nat Methods*. 2015;12(8):733-5. https://doi.org/10.1038/nmeth.3444.
96. Allen GC, Flores-Vergara MA, Krasynanski S, Kumar S and Thompson WF. A modified protocol for rapid DNA isolation from plant tissues using cetyltrimethylammonium bromide. *Nat Protoc.* 2006;1(5):2320-5. https://doi.org/10.1038/nprot.2006.384.

97. Krueger F and Andrews SR. Bismark: a flexible aligner and methylation caller for Bisulfite-Seq applications. *Bioinformatics.* 2011;27(11):1571-2. https://doi.org/10.1093/bioinformatics/btr167.
98. Cao S, Sawettalake N and Shen L. Supporting data for “Gapless genome assembly and epigenetic profiles reveal gene regulation of whole-genome triplication in lettuce". GigaScience Database 2024. https://doi.org/10.5524/102531

Table 1: Assembly statistics of the gapless genome of cutting lettuce CutV01.

Assembly metrics	PacBio-V1	PacBio-V2	CutV01
Technology	PacBio	PacBio+Hi-C	PacBio+ONT+Hi-C
Contig number	471	471	10
Contig sequence (bp)	2,597,591,538	2,597,591,538	2,580,598,114
Contig N/L50 (bp)	21,537,486	21,537,486	320,995,264
Contig N/L90 (bp)	8,533,704	8,533,704	230,643,374
Max contig length (bp)	73,015,125	73,015,125	408,427,630
Scaffold number (bp)	471	214	9
Scaffold sequence (bp)	2,597,591,538	2,597,720,038	2,580,598,614
Scaffold N/L50 (bp)	21,537,486	320,952,210	320,995,264
Scaffold N/L90 (bp)	8,533,704	230,490,360	230,643,374
Max scaffold length (bp)	73,015,125	408,496,717	408,463,181
Complete BUSCOs (C)	2,275	2,275	2,275
Complete and single-copy BUSCOs (S)	2,189	2,189	2,189
Complete and duplicated BUSCOs (D)	86	86	86
Fragmented BUSCOs (F)	13	13	13
Missing BUSCOs (M)	38	38	38

Table 2: Summary of CutV01 assembly and annotation.

Chromosome	Length (bp)	No. of Protein-coding genes	No. of Transcripts	Length of repeats
Chr1	252,054,568	4,176	6,885	236,866,928
Chr2	238,860,586	4,292	7,143	208,225,176
Chr3	320,995,264	4,578	7,165	287,668,740
Chr4	408,463,181	5,937	9,238	363,351,330
Chr5	372,789,172	5,594	9,102	330,269,961
Chr6	206,590,668	3,637	5,852	180,287,259
Chr7	208,637,244	4,033	6,061	182,159,041
Chr8	341,564,557	5,591	8,573	303,186,170
Chr9	230,643,374	4,568	7,104	200,914,520
**Total**	2,580,598,614	42,406	67,123	2,292,929,125

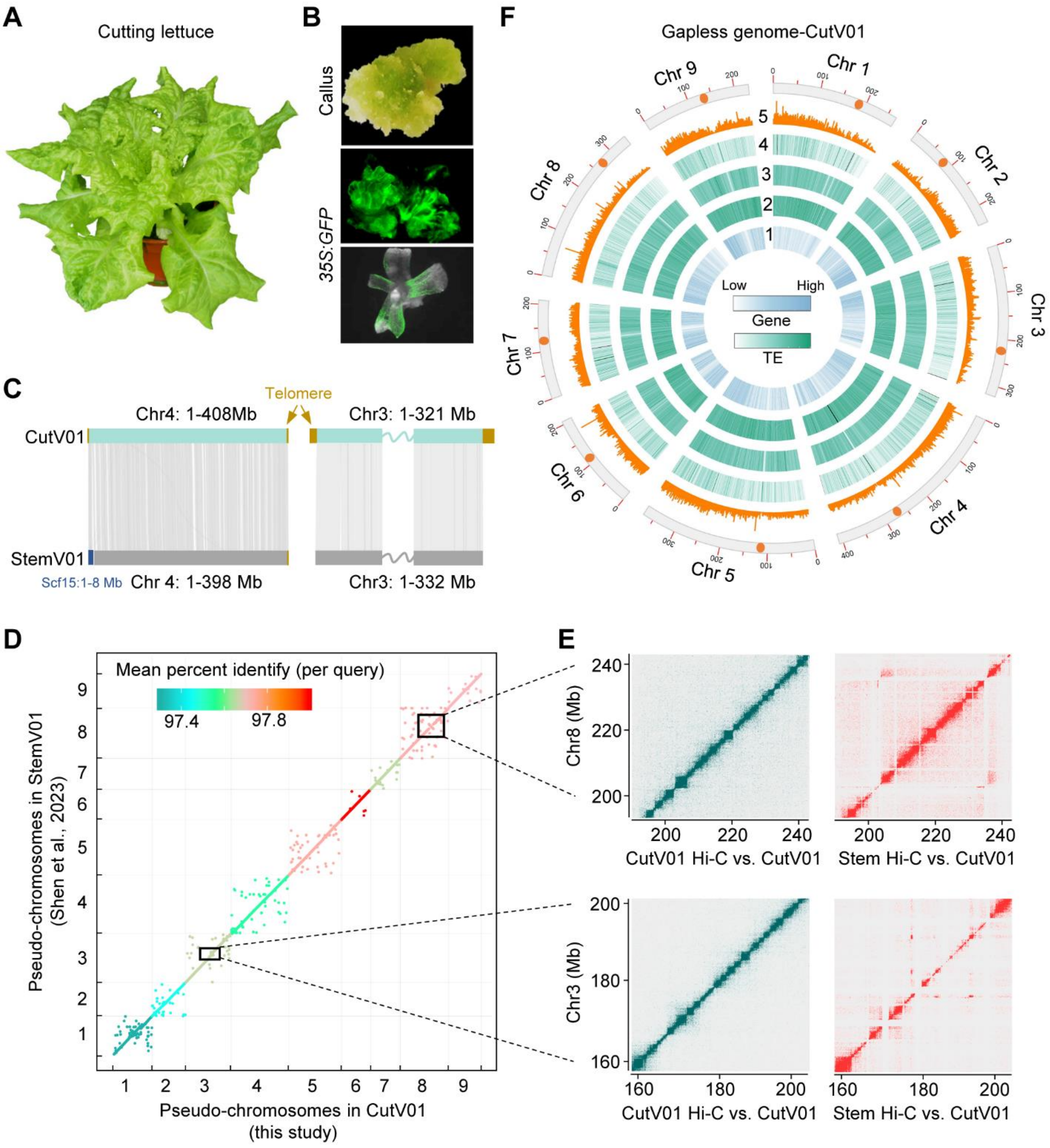

Figure 1: Gapless genome assembly of the cutting lettuce with high transformability. (A) A six-week-old cutting lettuce cultivar ‘Black Seeded Simpson’. (B) Regeneration and transformation of the cutting lettuce. Callus (upper panel) was induced from the excised cotyledon, and GFP fluorescence was exhibited by regenerated callus (middle panel) and shoot (low panel) transformed with *35S:GFP*. (C) The collinearity of the longest chromosome, Chr4 (left), and peri-telomeric regions of Chr3 (right) between cutting lettuce (CutV01) and stem lettuce (StemV01). Left peri-telomeric regions of Chr3: 1 - 1,024,348 of CutV01 and Chr3: 1 - 1,027,513 of StemV01 are shown, while right peri-telomeric regions of Chr3: 319,140,633 - 320,995,264 of CutV01 and Chr3: 330,587,218 – 332,303,623 of StemV01 are illustrated. (D) Dot plots of nucleotide alignment comparing the collinearity and similarity between the genomes of StemV01 and CutV01. Minimum nucleotide alignment length = 1 kb. Boxed regions represent inversions and rearrangements assessed using Hi-C data shown in (E). (E) The chromatin contact Hi-C maps validating the two large inversions

(20 - 30 Mb) in Chr8 and Chr3 present between the genomes of CutV01 and StemV01. (F) Circos plot depicting the features of chromosomes in CutV01 assembly. 1, gene density per Mb; 2, Gypsy density per Mb; 3, Copia density per Mb; 4; Density of LINEs per Mb; 5, Density of DNA Tes. All tracks are intensity-coded, with the color intensity indicating the frequency of each element. Pericentromere, represented by orange color, are depicted on the outmost track of chromosomes, with numbers indicating coordinates in Mb.

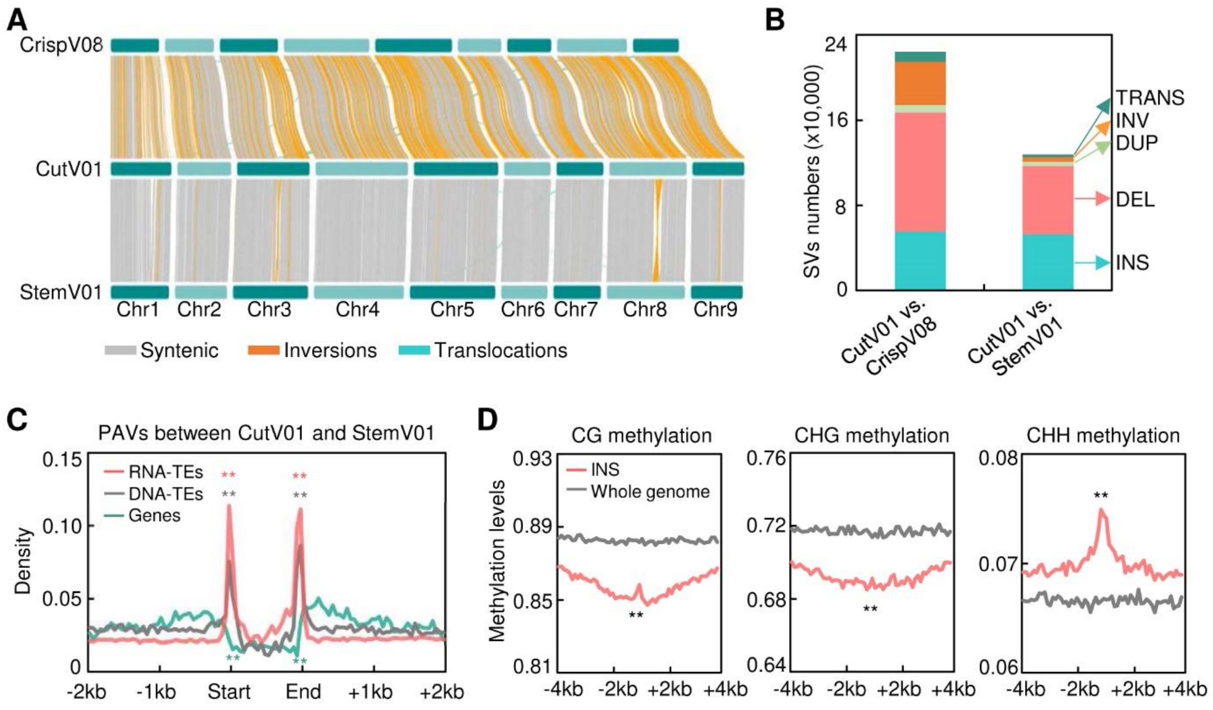

Figure 2: SVs in lettuce genome associated with DNA methylation. (A) Collinearity between the genomes of cutting lettuce (CutV01), crisp lettuce (CrispV08), and stem lettuce (StemV01). The yellow, blue, and grey linking blocks indicate inversions, translocations, and syntenic regions, respectively. (B) SVs identification in CutV01 in comparisons to CrispV08 and StemV01. The identified SVs include insertion (INS), deletion (DEL), duplication (DUP), inversion (INV), and translocation (TRANS). (C) Density of PAVs including insertion (INS) and deletion (DEL) between CutV01 and StemV01 in the gene regions, retrotransposons (RNA-TEs), and DNA-TEs. Asterisks indicate significance differences (***P* < 0.01, Wilcoxon signed-rank test) between the boundaries and flanking regions. (D) Average methylation levels of CG (left), CHG (middle), CHH (right) around insertions (INS) as compared to the whole genome. Asterisks indicate significance differences (***P* < 0.01, Wilcoxon signed-rank test).

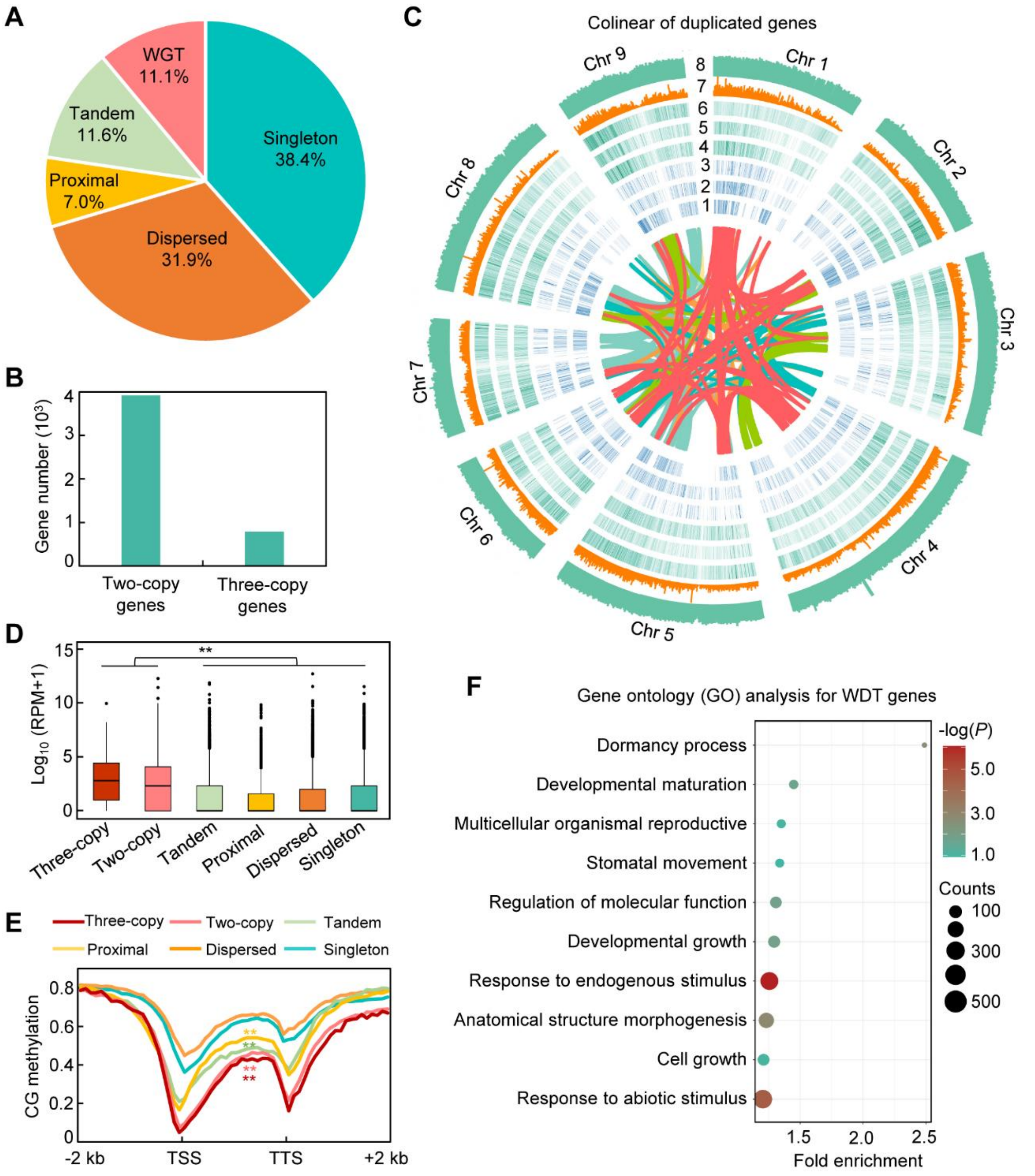

Figure 3: Features of whole-genome duplicated genes. (A) Percentage of WGT, tandem, proximal, dispersed, and single-copy (singleton) genes in annotated genes in CutV01a01. (B) The number of WGT genes retained with two copies and three copies from whole-genome triplication. (C) Collinearity of duplicated genes cross nine chromosomes. 1, three-copy genes density per Mb; 2, two-copy genes density per Mb; 3, Tandem genes density per Mb; 4, Proximal genes density per Mb; 5; Dispersed genes density per Mb; 6, Density of total genes; 7, Density of DNA TEs; 8, Density of RNA TEs. (D) Expression levels of three-copy orthologs, two-copy orthologs, tandem, proximal, dispersed, and singleton genes. The asterisk indicates a significant difference (***P* < 0.01, Wilcoxon signed-rank test). (E) Average CG methylation levels around three-copy genes, two-copy genes, small-scale duplicated (tandem, proximal, and dispersed), and single-copy genes. TSS, transcription start site; TTS, transcription termination site. Asterisks indicate significance differences between DNA methylation of indicated duplicated genes and singleton genes (***P* < 0.01, Wilcoxon signed-rank test). (F) GO enrichment of WGT genes. The plot shows the 10 top-scoring biological processes.

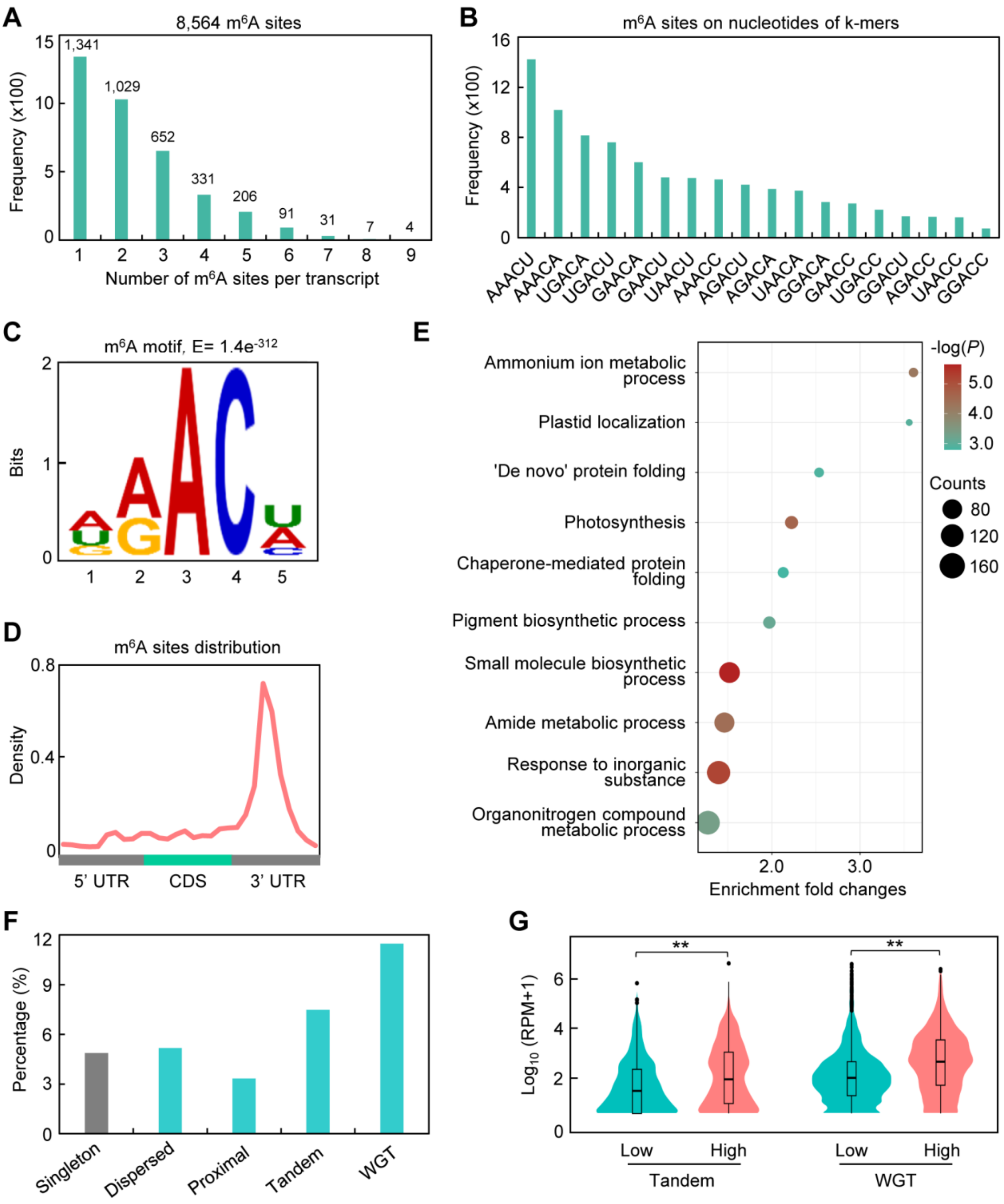

Figure 4: The expression of WGT genes modulated by m^{6}A modifications. (A) Frequency of numbers of m^{6}A sites per transcript. (B) Frequency of the top 5-bp k-mers at the positions with m^{6}A sites. (C) Sequence logo representing the consensus motif (DRACH) found in the m^{6}A sites. (D) Density of m^{6}A sites along the genic region, 5′ UTR, and 3′ UTR of transcripts. (E) GO enrichment of genes containing m^{6}A sites. The plot shows the 10 top-scoring biological processes. (F) Percentage of genes containing m^{6}A sites in each repeat type. (G) Expression levels of the tandem and WGT genes with low m^{6}A levels (Low) relative to all homoeologous genes with high m^{6}A (High). Asterisks indicate significance differences (**$P <$ 0.01, Wilcoxon signed-rank test).

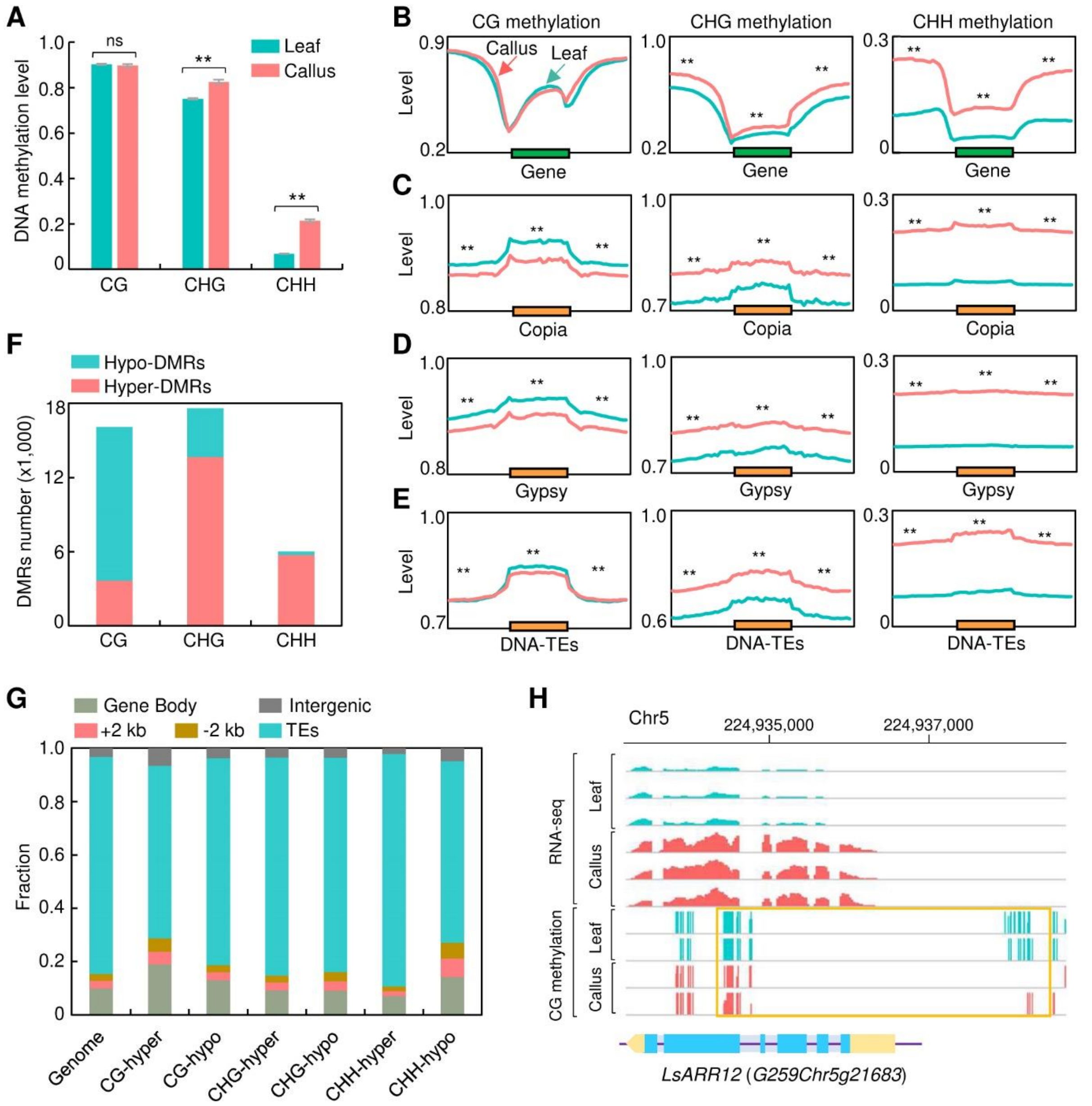

Figure 5: DNA methylation changes during callus formation. (A) Average DNA methylation level of CG, CHG, and CHH in lettuce calli and seedlings. Asterisks and ns indicate significance differences (**$P < 0.01$, Student's *t*-test) and no statistical differences ($P \geq 0.05$, Student's *t*-test), respectively. (B-E) DNA methylation levels of CG, CHG, and CHH on different genomic features including gene regions (B), Copia (C), Gypsy (D), and DNA-TEs (E). Asterisks indicate significance differences (**$P < 0.01$, Wilcoxon signed-rank test). (F) Number of hyper- and hypo-DMRs of CG, CHG, and CHH in calli compared to seedlings. (G) Distribution of DMRs in different genomic regions divided into gene body, +2 kb flanking region (2 kb upstream of TSS), -2 kb flanking region (2 kb downstream of TTS), TEs, and intergenic regions excluding TEs. (H) An example showing tissue-culture-induced low methylation states of the loci on Chr5: 224,934,400 - 224,938,500 (yellow box, upper panel) associated with changes in the expression of *LsARR12* (G259Chr5g21683). The gene structure of *LsARR12* was shown below, in which blue and yellow boxes indicate exons and untranslated regions, respectively, and the purple line indicates introns and other genomic regions.

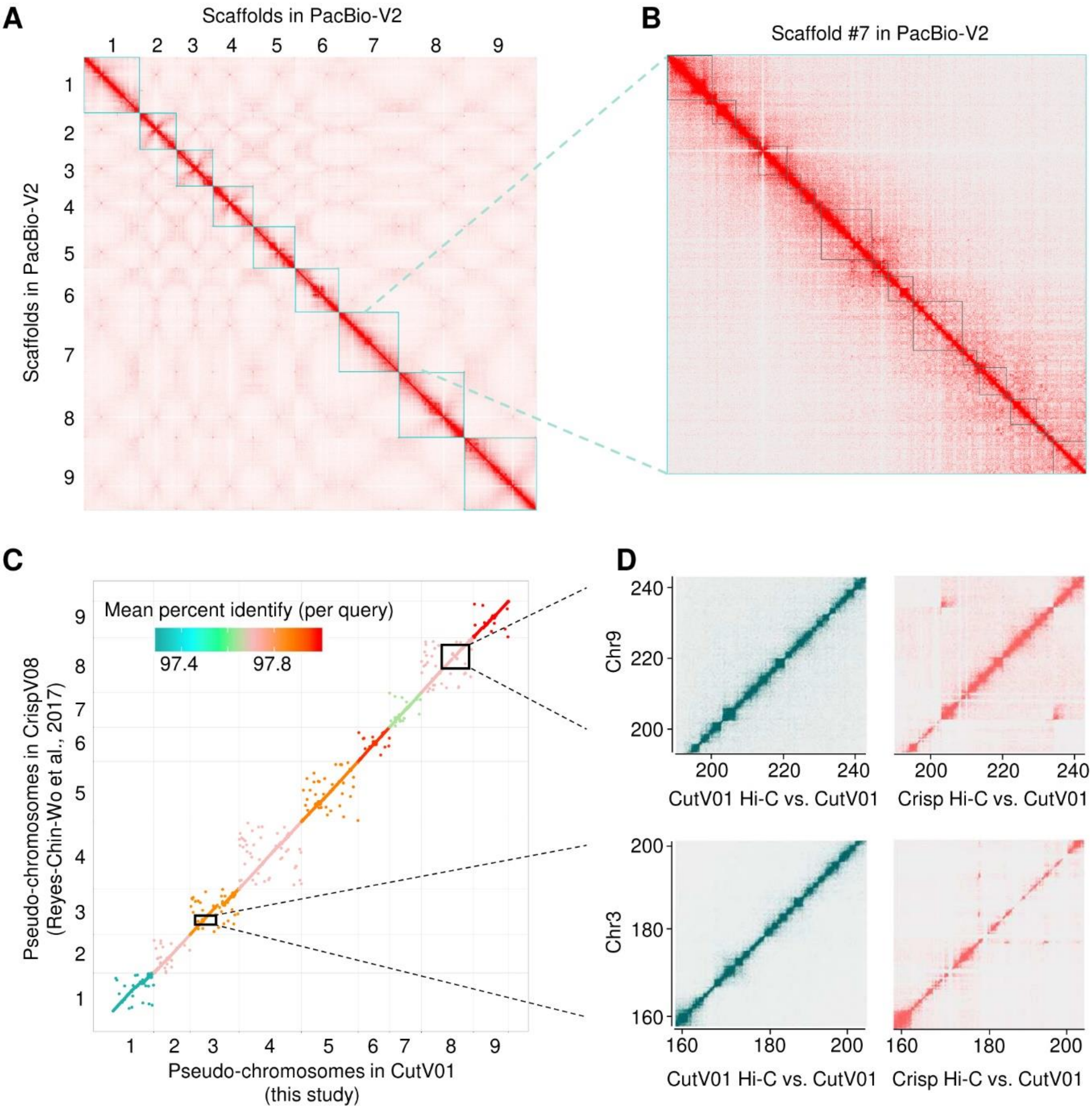

Supplementary Figure S1: Genome assembly for cutting lettuce. (A and B) Heat map displaying Hi-C interactions of pseudomolecules of all scaffolds (A) and scaffold #7 (B) in cutting lettuce. (C) Dot plots of nucleotide alignment comparing the collinearity and similarity between the genomes of CrispV08 and CutV01. Minimum nucleotide alignment length = 1 kb. Boxed regions represent inversions and rearrangements assessed using Hi-C data. (D) Chromatin contact Hi-C maps showing two large (20 - 30 Mb) inversions in Chr8 and Chr3 present in genomes of CutV01 compared to CrispV08.

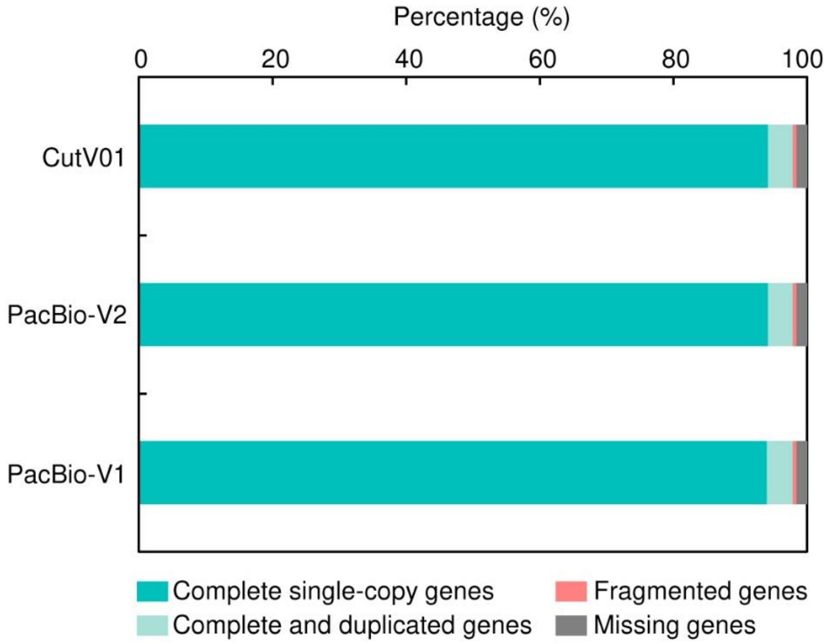

Supplementary Figure S2: BUSCO assessments for different versions of assembled genomes of cutting lettuce. BUSCO assessments reveal the percentages of genes classified into four categories: Complete and single-copy, Complete and duplicated, Fragmented, and Missing categories.

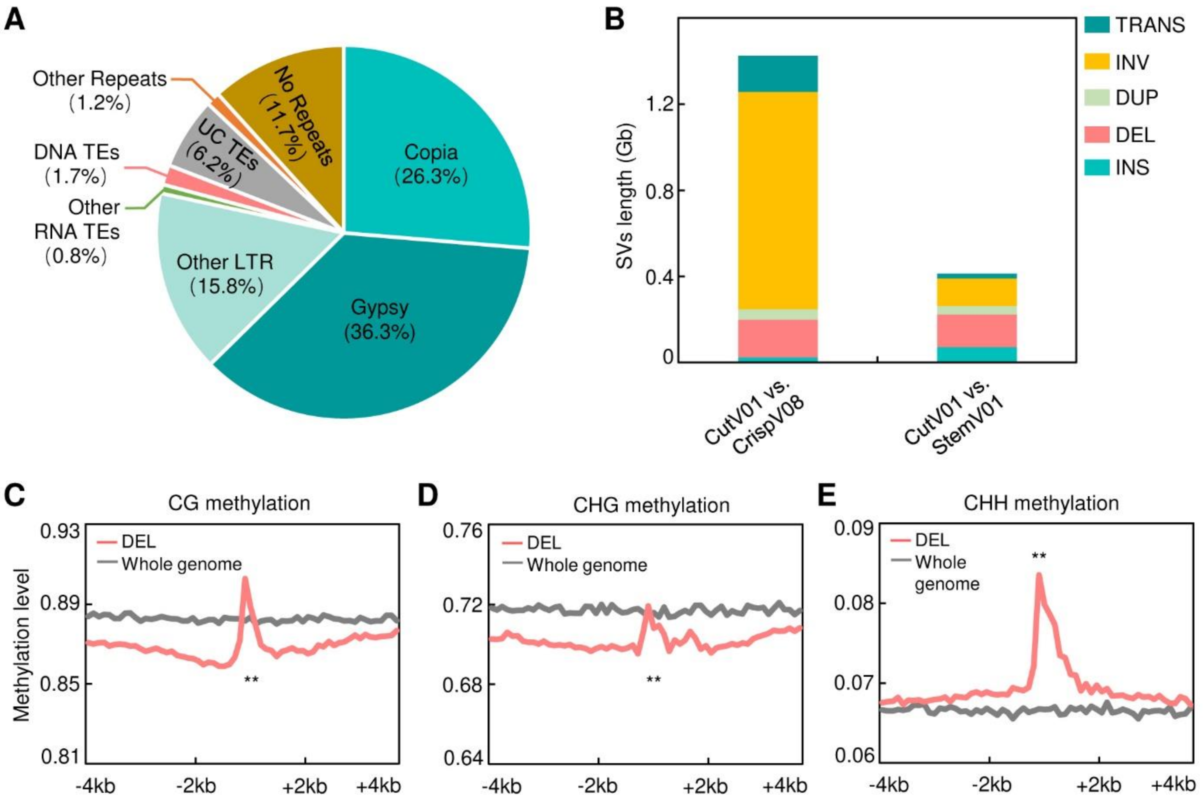

Supplementary Figure S3: Genomic features of the cutting lettuce genome CutV01. (A) Organization of repetitive sequences in CutV01, inducing RNA-TEs such as LTR retrotransposons (Copia, Gypsy, and others), other RNA TEs, as well as DNA TEs, unclassified TEs (UC TEs), and other repeats. (B) The length of SVs identified in CutV01 in comparison to CrispV08 and StemV01. SVs include insertion (INS), deletion (DEL), duplication (DUP), inversion (INV), and translocation (TRANS). (C-E) Average methylation levels of CG (C), CHG (D), CHH (E) around deletions (DEL) compared to the whole genome. Asterisks indicate significance differences (**$P < 0.01$, Wilcoxon signed-rank test).

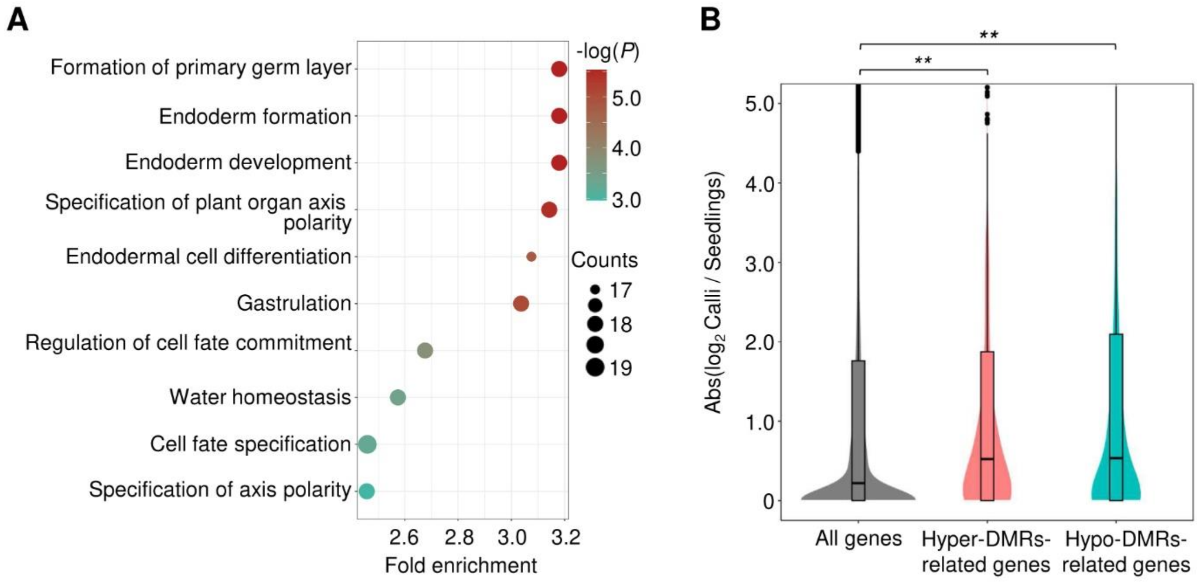

Supplementary Figure S4: Characterization of CG DMR-associated genes. (A) GO enrichment of genes associated with CG DMRs. The plot shows the 10 top-scoring biological processes. (B) Increased gene expression changes between calli and seedlings in CG hyper-DMR- and CG hypo-DMR-associated genes compared to all genes. Asterisks indicate significant differences (***P* < 0.01, Wilcoxon signed-rank test).

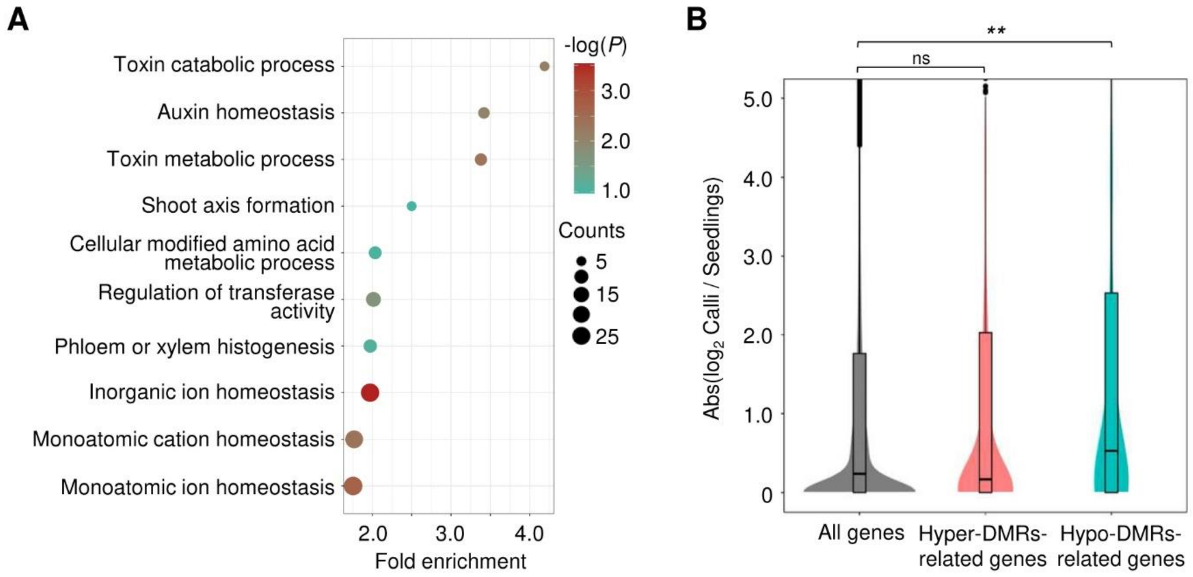

Supplementary Figure S5: Characterization of CHG DMR-associated genes. (A) GO enrichment of genes associated with CHG DMRs. The plot shows the 10 top-scoring biological processes. (B) Increased gene expression changes between calli and seedlings in CHG hypo-DMR-associated genes compared to all genes. An asterisk indicates a significant difference (**$P < 0.01$, Wilcoxon signed-rank test), and ns indicates no significant difference ($P \geq 0.05$, Wilcoxon signed-rank test).

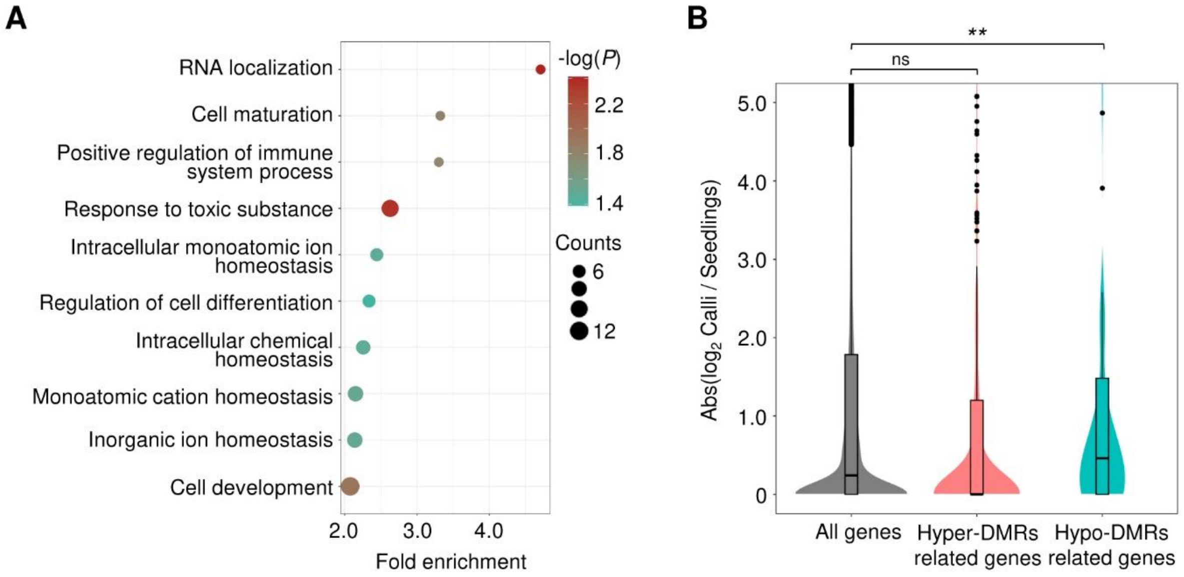

Supplementary Figure S6: Characterization of CHH DMR-associated genes. (A) GO enrichment of genes associated with CHH DMRs. The plot shows the 10 top-scoring biological processes. (B) Increased gene expression changes between calli and seedlings in CHH hypo-DMR-associated genes compared to all genes. The asterisk indicates a significant difference (***P* < 0.01, Wilcoxon signed-rank test), and ns indicates no significant difference ($P \geq 0.05$, Wilcoxon signed-rank test).

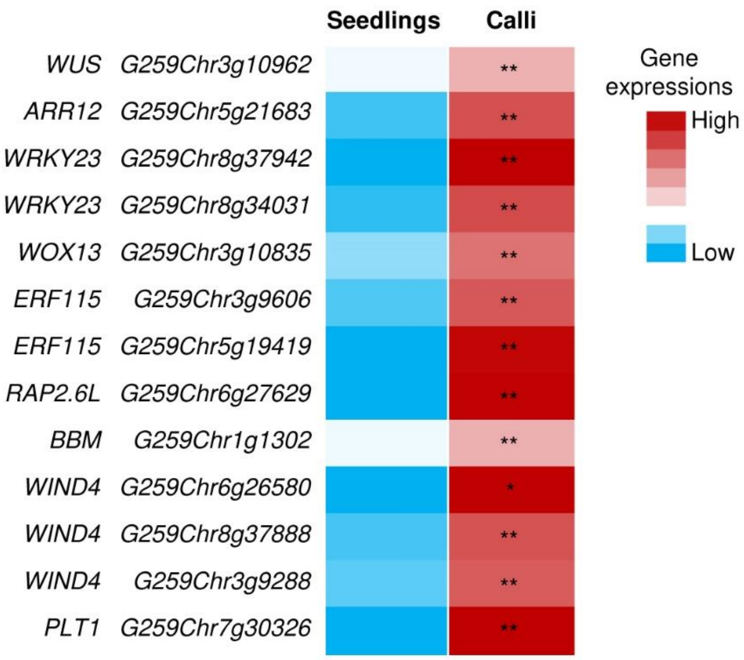

Supplementary Figure S7: Expression level of genes involved in callus formation. Asterisks indicate significance differences (*$P < 0.05$, **$P < 0.01$, two-tailed paired Student's *t*-test).

Click here to access/download
Supplementary Material
Lettuce gapless genome-SupplTable-1st rev.xlsx

Personal Cover
Click here to access/download;Personal Cover;Author response-1st rev.pdf

MS No.: GIGA-D-24-00037
MS Title: Gapless genome assembly and epigenetic profiles reveal gene regulation of whole-genome triplication in lettuce

We would like to thank the Editor and two reviewers for the time committed to reviewing this manuscript, and for the detailed suggestions on improving this manuscript. We have revised the manuscript to fully address Reviewer 1's comments as follows.

Reviewer #1

This works provides a high-quality and nearly telomere to telomere assembly of stem lettuce with is highly transformable. This is a valuable resource for the lettuce community. In particular, the analysis of CNVs and methylation (for example of transposons) is very informative.
>Author:
We appreciate the positive comments from the reviewer and suggestions on improving this manuscript.

A few points to consider for revision.

Major suggestion:
>Reviewer:
1. The comparisons to Salinas are all done to V08. However, on Genbank V11 is available. It would be extremely valuable to make some comparisons (such as synteny) to this V11 reference since it is already known that V08 has many errors and thus is no longer seen as the reference for lettuce.
>Author:
We thank the reviewer for this suggestion. We have used CrispV11 for SVs calling. As expected, we observed fewer SVs compared to those identified using CrispV08 in our current manuscript (see Figure R1 below). However, given the unpublished status of the paper reporting the CrispV11 genome, we are hesitant to incorporate the CrispV11 results into our manuscript. Nevertheless, to address the reviewer's comment, we have included the collinearity between the genomes of CrispV11 and CutV01 in this response file (Figure R1).

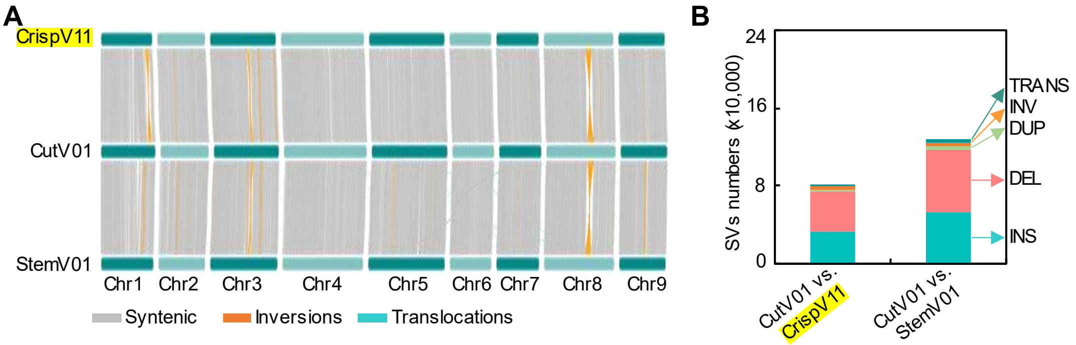

Figure R1: SVs in lettuce genome, related to Figure 2A,B. (A) Collinearity between the genomes of cutting lettuce (CutV01), crisp lettuce (CrispV11), and stem lettuce (StemV01). The yellow, blue, and grey linking blocks indicate inversions, translocations, and syntenic regions, respectively. (B) SVs identification in CutV01 in comparisons to CrispV11 and StemV01. The identified SVs include insertion (INS), deletion (DEL), duplication (DUP), inversion (INV), and translocation (TRANS).

>Reviewer:

2. The telomere analysis is convincing, however less is said about pericentromeres. Potentially considering this and its completeness would be interesting.

>Author:

We thank the reviewer for this suggestion. In the revised manuscript, we have identified the pericentromere regions of Cutv01 (the last two columns of new Supplementary Table S2) and found that these pericentromere regions (orange dots depicted on the outmost track of chromosomes; new Figure 1F) displayed a low density of genes. New Supplementary Table S2 and Figure 1F have been included in the revised manuscript.

ID of chromosomes	Length of chromosomes	Number of gaps	Number of shortarm telomers*	Direction of shortarm telomers	Number of longarm telomers	Direction of longarm telomers	Pericentromere start position	Pericentromere end position
Chr1	252,054,568	0	3,390	+	790	-	182,370,000	183,730,000
Chr2	238,860,586	0	766	+	1,080	-	91,950,000	93,870,000
Chr3	320,995,264	0	2,162	+	900	-	219,430,000	219,670,000
Chr4	408,463,181	1	1,090	+	453	-	275,140,000	277,340,000
Chr5	372,789,172	0	1,601	+	136	-	117,410,000	117,820,000
Chr6	206,590,668	0	979	+	2,986	-	106,990,000	107,850,000
Chr7	208,637,244	0	3,239	+	1,907	-	114,700,251	115,890,729
Chr8	341,564,557	0	865	+	0	NA	263,000,000	264,710,000
Chr9	230,643,374	0	1,324	+	1,966	-	132,240,000	134,930,000

New Supplementary Table 2. Lettuce genome sequence assembly organized into pseudochromosomes.

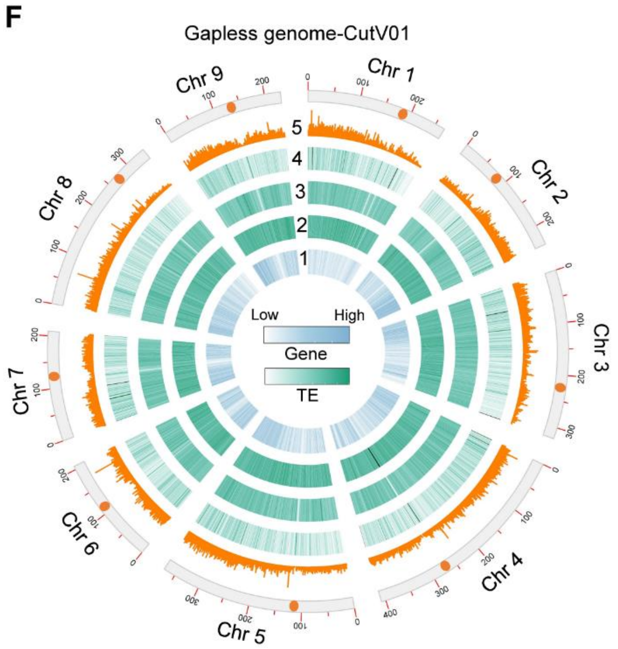

New Figure 1F. Circos plot depicting the features of chromosomes in CutV01 assembly. 1, gene density per Mb; 2, Gypsy density per Mb; 3, Copia density per Mb; 4; Density of LINEs per Mb; 5, Density of DNA Tes. All tracks are intensity-coded, with the color intensity indicating the frequency of each element. Pericentromere, represented by orange color, are depicted on the outmost track of chromosomes, with numbers indicating coordinates in Mb.

>Reviewer:

3. For transposon analysis, consider looking at reports of repeat content and types in recent paper on Lactuca virosa genome.

>Author:

As suggested, we have analyzed transposons based on the repeat content and types as recently reported in the *Lactuca virosa* genome (Xiong et al., 2023), and we have also included the representative LTR elements of Copia and Gypsy (new Supplementary Table S3). New Supplementary Table S3 has been included in the revised manuscript.

Classification	Length (bp)	Percent of assembled genome
SINEs	310,709	0.01%
LINEs	19,456,527	0.75%
LTR elements	1,881,310,407	72.90%
DNA transposons	44,634,312	1.73%
Unclassified	319,237,570	12.37%
Total TEs	2,264,949,525	87.77%
Other repeats:		
Small RNA	1,296,673	0.05%
Satellites	335,404	0.01%
Simple repeats	24,814,577	0.96%
Low complexity	1584352	0.06%
Representative LTR:		
LTR elements: Copia/Ty1	683,526,825	26.49%
LTR elements: Gypsy/DIRS1	942,585,779	36.53%

New Supplemental Table 3. Organization of repetitive sequences in the lettuce genome CutV01.

Relevant reference

Xiong W, van Workum DM, Berke L, Bakker LV, Schijlen E, Becker FFM, et al. Genome assembly and analysis of Lactuca virosa: implications for lettuce breeding. G3. 2023;13 11 doi:10.1093/g3journal/jkad204.

>Reviewer:

4. Line 208: Genes are said to be "4,706 whole-genome duplicated (WGD)". However, the genome is triplicated (as said). Thus, use Whole genome triplication (WGT) and break this category down into those in two copies or those in three. Perhaps looking at Brassica diploids (rapa or oleracea) also with WGT would be helpful. You do try to do this, but confusing to call WGD-T...this is not typical.

>Author:

We thank the reviewer for this suggestion. As suggested, in the revised text and new Figures 3 and 4, we have used WGT to replace WGD, and changed WGD-D/WGD-T to two-copy/three-copy genes as reported in the *Brassica rapa* genome (Wang et al., 2011).

Relevant reference

Wang X, Wang H, Wang J, Sun R, Wu J, Liu S, et al. The genome of the mesopolyploid crop species Brassica rapa. Nat Genet. 2011;43 10:1035-9.

>Reviewer:

5. For the analysis of WGT and duplicated genes (one copy lost) please also compare to earlier analyses (such as reference 11)

>Author:

As suggested, we have compared our analysis of WGT genes with the previous publication (ref 11: Barker et al., 2008). Barker et al. identified a total of 3,013 duplicated genes probably with two or three copies (namely WGT genes in our study) in 27,907 unigenes of lettuce, while in our study, we have identified 4,612 WGT genes in 42,406 annotated genes in the Cutv01 genome. Both numbers of WGT genes and total annotated genes are more than those reported in Barker et al. This is likely due to different gene annotation approaches. For gene annotation, we merged the annotation results from three approaches (ab initio gene

predication, homology-based gene predication, and RNA-seq-based transcriptomics data), but Barker et al., only used expressed sequence tag (EST) data. However, since the names or sequences of WGT genes are not provided in Baker et al., 2008, we are unable to further compare the identities of WGT genes in these two studies. We have added the relevant information in the Discussion part as follows: "Interestingly, we have identified 4,612 retained WGT genes, which are more than 3,013 genes reported in a previous study based on expressed sequence tag data [11].".

Relevant reference

Barker MS, Kane NC, Matvienko M, Kozik A, Michelmore RW, Knapp SJ, et al. Multiple paleopolyploidizations during the evolution of the Compositae reveal parallel patterns of duplicate gene retention after millions of years. Mol Biol Evol 2008;25(11):2445-55.

>Reviewer:

6. Many of the callus related genes also link to apomixis related factors, potentially check if Taraxacum Par gene is also unregulated?

>Author:

We thank the reviewer for this suggestion. PAR is a single-copy gene in lettuce (*Ls_8X112340*, *Lssex*) (Underwood et al., 2022). We performed a BLAST analysis using the protein sequence of Lssex in CrispV08 (no Lssex annotated in CrispV11) and identified Lssex encoded by *G259Chr8g36267* in Cutv01 with an identical protein sequence (see Figure R2 below). However, *G259Chr8g36267* is not detected in callus or leaf in our RNA-seq data.

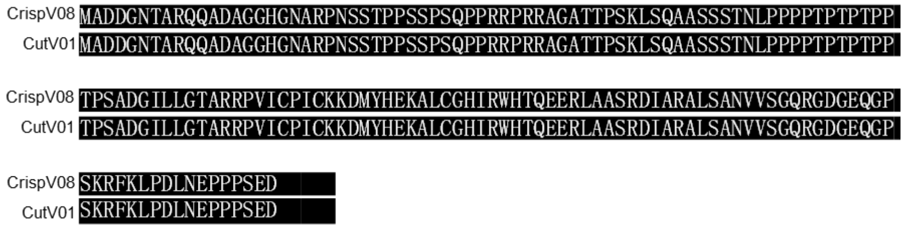

Figure R2. Protein sequence alignment of Lssex from CrispV08 (Ls_8X112340) and Cutv01 (G259Chr8g36267).

Relevant reference

Underwood CJ, Vijverberg K, Rigola D, Okamoto S, Oplaat C, Camp R, et al. A PARTHENOGENESIS allele from apomictic dandelion can induce egg cell division without fertilization in lettuce. Nat Genet. 2022;54 1:84-93.

Minor comments:

>Reviewer:

7. On lines 163 -164 it is said "Surprisingly, genomic collinearity and synteny analysis revealed that CutV01 has significantly more inversions in comparison to CrispV08 than to StemV0" But no statistical support or underlying numbers for this are given. Can this be improved?

>Author:

We thank the reviewer for this suggestion. To our knowledge, there is no appropriate statistical approach for this test. Therefore, in the revised manuscript,

we have changed this sentence to "Surprisingly, genomic collinearity and synteny analysis revealed that CutV01 has 1.74-fold more inversions in comparison to CrispV08 than to StemV01 (Fig. 2A).".

>Reviewer:
8. The number of significant digits (such as for % is sometimes one and sometimes two). Please choose one and be consistent
>Author:
As suggested, we have changed the significant digits of the results to consistently include two numbers after the decimal point in the revised manuscript.

<u>Reviewer #2</u>
This study aims to generate a new genome assembly for cutting lettuce. A near-complete telomere-to-telomere (T2T) genome assembly was generated for cutting lettuce (cultivar 'Black Seeded Simpson') using PacBio HiFi long reads, Hi-C data, and Oxford Nanopore ultra-long reads. The assembly spans 2.6 Gb and includes 7 complete T2T pseudo-chromosomes and 2 near-complete chromosomes, representing the highest quality lettuce genome assembly to date. This is one of the highest quality studies of complex plant genomes that I know of. The data analysis in this study also demonstrates the advantages of the new version of the genome. Therefore, I believe that in terms of the importance of the species, data accessibility, and data quality, this study deserves sufficient attention.
>Author:
We appreciate the supportive comments from this reviewer.

Personal Cover Click here to access/download;Personal Cover;Cover letter-1st rev.pdf

MS No.: GIGA-D-24-00037
MS title: Gapless genome assembly and epigenetic profiles reveal gene regulation of whole-genome triplication in lettuce

Dear Dr. Hongfang Zhang,

We would like to thank you and two reviewers for the time committed to reviewing our manuscript, and for the comments on improving this manuscript. We have revised the manuscript to fully address the comments from Reviewer 1.

As suggested by Reviewer 1, we have analyzed the pericentromere regions (new Figure 1F and Supplementary Table S2). We have also performed transposon analysis based on the repeat content and types as recently reported in the *Lactuca virosa* genome (new Supplementary Table S3) as suggested.

In addition to the above revisions, we have addressed all the other comments raised by Reviewer 1. Our point-to-point responses to Reviewer 1's comments are provided. Meanwhile, the RRIDs from SciCrunch.org were added for the new software applications in this manuscript. These changes have been highlighted in yellow in the revised manuscript. Moreover, we have formatted the manuscript according to the provided instructions, including citing reference #98 that represents "Cao S, Sawettalake N and Shen L. Supporting data for "Gapless genome assembly and epigenetic profiles reveal gene regulation of whole-genome triplication in lettuce". GigaScience Database 2024.".

With these revisions, we hope that the manuscript is now acceptable for publication.

Thank you for your consideration and help.

Sincerely yours,

Lisha Shen

Temasek Life Science Laboratory
National University of Singapore
Singapore 117604
Email: lisha@tll.org.sg
Website: http://www.tll.org.sg/group-leaders/shen-lisha/
