# Supplementary material for: Gapless genome assembly and epigenetic profiles reveal gene regulation of whole-genome triplication in lettuce
Source: Gigascience. 2024 Jul 11;13:giae043. doi: 10.1093/gigascience/giae043 (PMC11238431; doi:10.1093/gigascience/giae043)
Supplement: giae043_Response_to_Reviewer_Comments_Original_Submission [file giae043_response_to_reviewer_comments_original_submission.pdf]

Author's Response To Reviewer Comments

MS No.: GIGA-D-24-00037
MS Title: Gapless genome assembly and epigenetic profiles reveal gene regulation of whole-genome triplication in lettuce

We would like to thank the Editor and two reviewers for the time committed to reviewing this manuscript, and for the detailed suggestions on improving this manuscript. We have revised the manuscript to fully address Reviewer 1’s comments as follows.

Reviewer #1

This works provides a high-quality and nearly telomere to telomere assembly of stem lettuce with is highly transformable. This is a valuable resource for the lettuce community. In particular, the analysis of CNVs and methylation (for example of transposons) is very informative.
>Author:
We appreciate the positive comments from the reviewer and suggestions on improving this manuscript.

A few points to consider for revision.

Major suggestion:
>Reviewer:
1. The comparisons to Salinas are all done to V08. However, on Genbank V11 is available. It would be extremely valuable to make some comparisons (such as synteny) to this V11 reference since it is already known that V08 has many errors and thus is no longer seen as the reference for lettuce.
>Author:
We thank the reviewer for this suggestion. We have used CrispV11 for SVs calling. As expected, we observed fewer SVs compared to those identified using CrispV08 in our current manuscript (see Figure R1 in the attached Author Response file). However, given the unpublished status of the paper reporting the CrispV11 genome, we are hesitant to incorporate the CrispV11 results into our manuscript. Nevertheless, to address the reviewer’s comment, we have included the collinearity between the genomes of CrispV11 and CutV01 in the attached Author Response file (Figure R1).

>Reviewer:
2. The telomere analysis is convincing, however less is said about pericentromeres. Potentially considering this and its completeness would be interesting.
>Author:
We thank the reviewer for this suggestion. In the revised manuscript, we have identified the pericentromere regions of Cutv01 (the last two columns of new Supplementary Table S2) and found that these pericentromere regions (orange dots depicted on the outmost track of chromosomes; new Figure 1F) displayed a low density of genes. New Supplementary Table S2 and Figure 1F have been included in the revised manuscript.

>Reviewer:
3. For transposon analysis, consider looking at reports of repeat content and types in recent paper on Lactuca virosa genome.
>Author:
As suggested, we have analyzed transposons based on the repeat content and types as recently reported in the Lactuca virosa genome (Xiong et al., 2023), and we have also included the representative LTR elements of Copia and Gypsy (new Supplementary Table S3). New Supplementary Table S3 has been included in the revised manuscript.

Relevant reference
Xiong W, van Workum DM, Berke L, Bakker LV, Schijlen E, Becker FFM, et al. Genome assembly and analysis of Lactuca virosa: implications for lettuce breeding. G3. 2023;13 11

doi:10.1093/g3journal/jkad204.

>Reviewer:
4. Line 208: Genes are said to be "4,706 whole-genome duplicated (WGD)". However, the genome is triplicated (as said). Thus, use Whole genome triplication (WGT) and break this category down into those in two copies or those in three. Perhaps looking at Brassica diploids (rapa or oleracea) also with WGT would be helpful. You do try to do this, but confusing to call WGD-T...this is not typical.
>Author:
We thank the reviewer for this suggestion. As suggested, in the revised text and new Figures 3 and 4, we have used WGT to replace WGD, and changed WGD-D/WGD-T to two-copy/three-copy genes as reported in the Brassica rapa genome (Wang et al., 2011).

Relevant reference
Wang X, Wang H, Wang J, Sun R, Wu J, Liu S, et al. The genome of the mesopolyploid crop species Brassica rapa. Nat Genet. 2011;43 10:1035-9.

>Reviewer:
5. For the analysis of WGT and duplicated genes (one copy lost) please also compare to earlier analyses (such as reference 11)
>Author:
As suggested, we have compared our analysis of WGT genes with the previous publication (ref 11: Barker et al., 2008). Barker et al. identified a total of 3,013 duplicated genes probably with two or three copies (namely WGT genes in our study) in 27,907 unigenes of lettuce, while in our study, we have identified 4,612 WGT genes in 42,406 annotated genes in the Cutv01 genome. Both numbers of WGT genes and total annotated genes are more than those reported in Barker et al. This is likely due to different gene annotation approaches. For gene annotation, we merged the annotation results from three approaches (ab initio gene predication, homology-based gene predication, and RNA-seq-based transcriptomics data), but Barker et al., only used expressed sequence tag (EST) data. However, since the names or sequences of WGT genes are not provided in Baker et al., 2008, we are unable to further compare the identities of WGT genes in these two studies. We have added the relevant information in the Discussion part as follows: “Interestingly, we have identified 4,612 retained WGT genes, which are more than 3,013 genes reported in a previous study based on expressed sequence tag data [11].”.

Relevant reference
Barker MS, Kane NC, Matvienko M, Kozik A, Michelmore RW, Knapp SJ, et al. Multiple paleopolyploidizations during the evolution of the Compositae reveal parallel patterns of duplicate gene retention after millions of years. Mol Biol Evol 2008;25(11):2445-55.

>Reviewer:
6. Many of the callus related genes also link to apomixis related factors, potentially check if Taraxacum Par gene is also unregulated?
>Author:
We thank the reviewer for this suggestion. PAR is a single-copy gene in lettuce (Ls_8X112340, Lssex) (Underwood et al., 2022). We performed a BLAST analysis using the protein sequence of Lssex in CrispV08 (no Lssex annotated in CrispV11) and identified Lssex encoded by G259Chr8g36267 in Cutv01 with an identical protein sequence (see Figure R2 in the attached Author Response file). However, G259Chr8g36267 is not detected in callus or leaf in our RNA-seq data.

Relevant reference
Underwood CJ, Vijverberg K, Rigola D, Okamoto S, Oplaat C, Camp R, et al. A PARTHENOGENESIS allele from apomictic dandelion can induce egg cell division without fertilization in lettuce. Nat Genet. 2022;54 1:84-93.

Minor comments:
>Reviewer:
7. On lines 163 -164 it is said "Surprisingly, genomic collinearity and synteny analysis revealed that CutV01 has significantly more inversions in comparison to CrispV08 than to StemV0" But no statistical support or

underlying numbers for this are given. Can this be improved?
>Author:
We thank the reviewer for this suggestion. To our knowledge, there is no appropriate statistical approach for this test. Therefore, in the revised manuscript, we have changed this sentence to "Surprisingly, genomic collinearity and synteny analysis revealed that CutV01 has 1.74-fold more inversions in comparison to CrispV08 than to StemV01 (Fig. 2A).".

>Reviewer:
8. The number of significant digits (such as for % is sometimes one and sometimes two). Please choose one and be consistent
>Author:
As suggested, we have changed the significant digits of the results to consistently include two numbers after the decimal point in the revised manuscript.

Reviewer #2
This study aims to generate a new genome assembly for cutting lettuce. A near-complete telomere-to-telomere (T2T) genome assembly was generated for cutting lettuce (cultivar 'Black Seeded Simpson') using PacBio HiFi long reads, Hi-C data, and Oxford Nanopore ultra-long reads. The assembly spans 2.6 Gb and includes 7 complete T2T pseudo-chromosomes and 2 near-complete chromosomes, representing the highest quality lettuce genome assembly to date. This is one of the highest quality studies of complex plant genomes that I know of. The data analysis in this study also demonstrates the advantages of the new version of the genome. Therefore, I believe that in terms of the importance of the species, data accessibility, and data quality, this study deserves sufficient attention.
>Author:
We appreciate the supportive comments from this reviewer.
