# Supplementary material for: Gapless genome assembly and epigenetic profiles reveal gene regulation of whole-genome triplication in lettuce
Source: Gigascience. 2024 Jul 11;13:giae043. doi: 10.1093/gigascience/giae043 (PMC11238431; doi:10.1093/gigascience/giae043)
Supplement: giae043_Reviewer_1_Report_Original_Submission — Eric Schranz -- 3/31/2024 Reviewed [file giae043_reviewer_1_report_original_submission.pdf]

Reviewer Report

Title: Gapless genome assembly and epigenetic profiles reveal gene regulation of whole-genome triplication in lettuce

Version: Original Submission **Date:** 3/31/2024

Reviewer name: Eric Schranz

Reviewer Comments to Author:

This works provides a high-quality and nearly telomere to telomere assembly of stem lettuce with is highly transformable. This is a valuable resource for the lettuce community. In particular, the analysis of CNVs and methylation (for example of transposons) is very informative.
A few points to consider for revision.
Major suggestion: The comparisons to Salinas are all done to V08. However, on Genbank V11 is available. It would be extremely valuable to make some comparisons (such as synteny) to this V11 reference since it is already known that V08 has many errors and thus is no longer seen as the reference for lettuce.
The telomere analysis is convincing, however less is said about pericentromeres. Potentially considering this and its completeness would be interesting.
For transposon analysis, consider looking at reports of repeat content and types in recent paper on Lactuca virosa genome.
Line 208: Genes are said to be "4,706 whole-genome duplicated (WGD)". However, the genome is triplicated (as said). Thus, use Whole genome triplication (WGT) and break this category down into those in two copies or those in three. Perhaps looking at Brassica diploids (rapa or oleracea) also with WGT would be helpful. You do try to do this, but confusing to call WGD-T...this is not typical.
For the analysis of WGT and duplicated genes (one copy lost) please also compare to earlier analyses (such as reference 11)
Many of the callus related genes also link to apomixis related factors, potentially check if Taraxacum Par gene is also unregulated?
Minor comments:
On lines 163 -164 it is said "Surprisingly, genomic collinearity and synteny analysis revealed that CutV01 has significantly more inversions in comparison to CrispV08 than to StemV0" But no statistical support or underlying numbers for this are given. Can this be improved?
The number of significant digits (such as for % is sometimes one and sometimes two). Please choose one and be consistent

Methods

Are the methods appropriate to the aims of the study, are they well described, and are necessary controls included? Choose an item.

Conclusions

Are the conclusions adequately supported by the data shown? Choose an item.

Reporting Standards

Does the manuscript adhere to the journal's guidelines on minimum standards of reporting? Choose an item.

Choose an item.

Statistics

Are you able to assess all statistics in the manuscript, including the appropriateness of statistical tests used? Choose an item.

Quality of Written English

Please indicate the quality of language in the manuscript: Choose an item.

Declaration of Competing Interests

Please complete a declaration of competing interests, considering the following questions:

- Have you in the past five years received reimbursements, fees, funding, or salary from an organisation that may in any way gain or lose financially from the publication of this manuscript, either now or in the future?
- Do you hold any stocks or shares in an organisation that may in any way gain or lose financially from the publication of this manuscript, either now or in the future?
- Do you hold or are you currently applying for any patents relating to the content of the manuscript?
- Have you received reimbursements, fees, funding, or salary from an organization that holds or has applied for patents relating to the content of the manuscript?
- Do you have any other financial competing interests?
- Do you have any non-financial competing interests in relation to this paper?

If you can answer no to all of the above, write 'I declare that I have no competing interests' below. If your reply is yes to any, please give details below.

'I declare that I have no competing interests'

I agree to the open peer review policy of the journal. I understand that my name will be included on my report to the authors and, if the manuscript is accepted for publication, my named report including any attachments I upload will be posted on the website along with the authors' responses. I agree for my report to be made available under an Open Access Creative Commons CC-BY license (http://creativecommons.org/licenses/by/4.0/). I understand that any comments which I do not wish to

be included in my named report can be included as confidential comments to the editors, which will not be published.

Choose an item.

To further support our reviewers, we have joined with Publons, where you can gain additional credit to further highlight your hard work (see: https://publons.com/journal/530/gigascience). On publication of this paper, your review will be automatically added to Publons, you can then choose whether or not to claim your Publons credit. I understand this statement.

Yes Choose an item.
